# A super minigene with a short promoter and truncated introns recapitulates essential features of transcription and splicing regulation of the *SMN1* and *SMN2* genes

**DOI:** 10.1093/nar/gkad1259

**Published:** 2024-01-12

**Authors:** Eric W Ottesen, Joonbae Seo, Diou Luo, Natalia N Singh, Ravindra N Singh

**Affiliations:** Department of Biomedical Sciences, Iowa State University, Ames, IA 50011, USA; Department of Biomedical Sciences, Iowa State University, Ames, IA 50011, USA; Department of Biomedical Sciences, Iowa State University, Ames, IA 50011, USA; Department of Biomedical Sciences, Iowa State University, Ames, IA 50011, USA; Department of Biomedical Sciences, Iowa State University, Ames, IA 50011, USA

## Abstract

Here we report a *Survival Motor Neuron 2* (*SMN2*) super minigene, *SMN2^Sup^*, encompassing its own promoter, all exons, their flanking intronic sequences and the entire 3′-untranslated region. We confirm that the pre-mRNA generated from *SMN2^Sup^* undergoes splicing to produce a translation-competent mRNA. We demonstrate that mRNA generated from *SMN2^Sup^* produces more SMN than an identical mRNA generated from a cDNA clone. We uncover that overexpression of SMN triggers skipping of exon 3 of *SMN1*/*SMN2*. We define the minimal promoter and regulatory elements associated with the initiation and elongation of transcription of *SMN2*. The shortened introns within *SMN2^Sup^* preserved the ability of camptothecin, a transcription elongation inhibitor, to induce skipping of exons 3 and 7 of *SMN2*. We show that intron 1-retained transcripts undergo nonsense-mediated decay. We demonstrate that splicing factor SRSF3 and DNA/RNA helicase DHX9 regulate splicing of multiple exons in the context of both *SMN2^Sup^* and endogenous *SMN1*/*SMN2*. Prevention of *SMN2* exon 7 skipping has implications for the treatment of spinal muscular atrophy (SMA). We validate the utility of the super minigene in monitoring SMN levels upon splicing correction. Finally, we demonstrate how the super minigene could be employed to capture the cell type-specific effects of a pathogenic *SMN1* mutation.

## Graphical Abstract

**Graphical Abstract:**
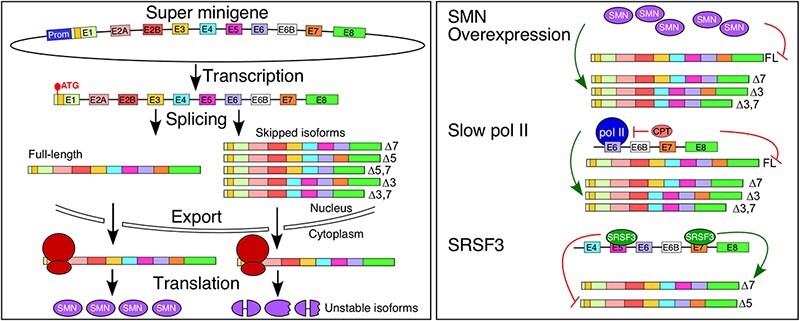

## Introduction

Humans carry two nearly identical copies of *Survival Motor Neuron* genes, *SMN1* and *SMN2* (1). Low levels of SMN protein due to deletions or mutations of *SMN1* lead to spinal muscular atrophy (SMA), a major genetic disease linked to infant mortality (2–5). SMN is a multifunctional protein involved in essential cellular processes, such as DNA repair and replication, transcription, pre-mRNA splicing, translation, mRNA trafficking, signaling mechanisms and cytoskeletal dynamics (6). *SMN2* is generally unable to compensate for the loss of *SMN1* due to predominant exon 7 skipping that results in production of SMNΔ7, a truncated protein that is highly unstable (7–9). Although the specific functions of *SMN2* remain unknown, a splicing switch from skipping to inclusion of *SMN2* exon 7 is attributed, at least in part, to maintaining high SMN levels in adult testis (10). Elevated levels of SMN and/or SMNΔ7 due to high *SMN2* copy numbers reduce the severity of SMA (4). Nusinersen, the first approved therapy for SMA, is an antisense oligonucleotide (ASO) that restores *SMN2* exon 7 inclusion upon binding to the intronic splicing silencer N1 (ISS-N1) located immediately downstream of the 5′ splice site (5′ss) of exon 7 (11,12). Risdiplam, an orally administered small molecule recently approved for SMA therapy, also works through restoration of *SMN2* exon 7 inclusion (13). Several additional small molecules capable of modulating *SMN2* transcription and splicing are currently at different stages of pre-clinical and clinical trials (4,14).

Both *SMN1* and *SMN2* genes contain nine exons, i.e. exons 1, 2A, 2B, 3, 4, 5, 6, 7 and 8 (15). Exons 1–7 code for SMN and exon 8 constitutes the 3′-untranslated region (3′UTR) (15). Critical domains associated with SMN functions are distributed throughout the protein. These include a nucleic acid-binding domain towards the N-terminus, Tudor and proline-rich domains in the middle and a self-oligomerization domain at the C-terminus (6). Skipping of exon 7 results in replacement of the 16 C-terminal amino acids encoded by this exon with four amino acids encoded by exon 8 (EMLA), which in turn leads to disruption of the self-oligomerization domain and creation of a degradation signal responsible for the high instability of SMNΔ7 (9). A shorter protein, a-SMN, comprised of the N-terminal half of SMN, is coded by the intron 3-retained transcripts of *SMN* (16). Some *SMN* transcripts harbor an Alu-derived exon 6B that changes the critical C-terminus of SMN (17). Difficulties in detection of the intron 3-retained and exon 6B-containing transcripts of *SMN* are likely to be due to their degradation by nonsense-mediated decay (NMD) (17). The *SMN* genes generate a vast repertoire of circular RNAs (circRNAs) through backsplicing assisted by abundant Alu repeats (18–20). Currently it is not known if Alu repeats also regulate forward splicing of internal *SMN* exons. DHX9 is a DExD/H-box helicase that unwinds RNA:RNA, DNA:RNA and other types of helices (21–23). DHX9 causes transcriptome-wide suppression of circRNA generation through disruption of RNA:RNA helices formed by inverted Alu repeats (24). Consistently, depletion of DHX9 has been shown to enhance circRNA generation from the Alu-rich *SMN* genes (19,20). Supporting the role of DHX9-sensitive secondary structures in splicing regulation of *SMN* exons, depletion of DHX9 promotes skipping of multiple *SMN* exons (19). Currently it is not known if DHX9 can also modulate *SMN* splicing or expression through secondary structures formed by sequences other than Alu elements. Several *cis-*elements within *SMN* exon 7 have been implicated in the regulation of its splicing (25). However, limited attention has been paid to splicing regulation of *SMN1/SMN2* exons 3 and 5 that also undergo skipping and co-skipping in different combinations (26).

Two seminal studies performed in 1999 independently concluded that the promoters of *SMN1* and *SMN2* are structurally and functionally equivalent (27,28). However, these studies did not critically analyze the core promoter and the promoter-proximal regions. In addition, there is no study on how mutations within the *SMN* promoter might affect the transcription start site (TSS) and consequently the levels of SMN. Splicing is generally coupled to transcription, as the rate of transcription elongation by RNA polymerase II (pol II) determines the fate of splicing of specific exons (29). Innovative techniques, including native elongating transcript sequencing (NET-seq) and precision nuclear run-on and sequencing (PRO-seq), capture pause sites of pol II and have potential to provide a direct link between transcription elongation at specific genomic locations and pre-mRNA splicing (30). Slow-moving pol II promotes inclusion and skipping of class I and class II exons, respectively (31). A recent report suggests that *SMN2* exon 7 belongs to the class II category because its skipping is promoted by a slow pol II (32). However, it is not known if slow elongation rates could trigger skipping of other *SMN1* or *SMN2* exons. It is also not known if the intronic Alu elements can modulate the effect of the slow poll II on splicing of *SMN1/SMN2* exons.

Here we report the generation of an *SMN2* super minigene comprised of all exons and their flanking intronic sequences. The *SMN2* super minigene recapitulates the splicing pattern of endogenous *SMN2*. Using this construct, we identify novel regulatory elements associated with transcription regulation and TSS selection. We demonstrate that more protein is expressed from *SMN* transcripts that undergo pre-mRNA splicing than from a cDNA clone. We show that overexpression of SMN or SMNΔ7 has a negative effect on splicing of exon 3 of *SMN1*/*SMN2*. We examine the consequences of intron 1 retention on splicing and translation of *SMN2* transcripts. We uncover the susceptibility of multiple *SMN1*/*SMN2* exons to skipping when transcription elongation is inhibited. We show the effect of SRSF3 on transcription, splicing and translation of transcripts generated from the super minigene. We examine the effect of DHX9 on splicing of *SMN2* exons in the context of shortened introns lacking Alu elements. We show the utility of the super minigene as a reporter for screening of splicing-modulating compounds. We also demonstrate how the super minigene could be employed to monitor the effect of a pathogenic mutation of *SMN1* on transcription, splicing and translation.

## Materials and methods

### Construction of the *SMN2* super minigene

All minigenes used in this study had a backbone of a pCI or pCI-Neo mammalian expression vector (Promega). Restriction enzymes, Quick Ligase and Phusion High-Fidelity DNA polymerase were purchased from New England Biolabs. To generate the first *SMN2* super minigene, *SMN2^Sup3445^*, we used a multi-step polymerase chain reaction (PCR)-based approach (Supplementary Figure S1). Three copies of the FLAG epitope (33) were inserted in-frame immediately downstream of the ATG start codon to monitor for protein expressed from *SMN2^Sup^*. Templates for PCR were either the *SMN2ΔI6* minigene (34) or genomic DNA from *Homo sapiens* chromosome 5 clone CTC-566F17 (GenBank: AC022119.4). Each PCR fragment was generated stepwise in the order indicated (Supplementary Figure S1) using Phusion High-Fidelity DNA polymerase. The individual fragments were then joined together by PCR to generate intermediate products, which were subsequently cloned into pGEM-T-easy vector, and then subcloned into the *SMN2ΔI6* minigene cut with BglII and XhoI to generate a final product. We validated all clones by Sanger sequencing.

To reduce ‘leaky’ transcription that we observed for our promoterless super minigenes, four copies of the transcriptional pause site (4×TPS) were cloned into *SMN2^Sup3445^* using BglII and SalI sites. TPS sequences were amplified by PCR using pGL3-Basic vector as a template. All deletions and mutations were generated by two-step PCR using the appropriate super minigene as a template. PCR products were then digested with appropriate restriction enzymes and inserted into the appropriate super minigene treated with the same enzymes. Primers used for PCR were purchased from Integrated DNA Technologies (IDT) or the DNA facility at Iowa State University. All primers used in this study are given in Supplementary Table S1. Sequences of key super minigenes were submitted to NCBI Genbank. Accession numbers of super minigene sequences are given in Supplementary Table S2.

### Cell culture and transfection

All cell culture reagents and media were obtained from Life Technologies unless otherwise specified. Human cervical adenocarcinoma (HeLa), NSC34 and human embryonic kidney HEK-293 (HEK) cells were obtained from the American Type Culture Collection (ATCC) and were grown in Dulbecco's modified Eagle's medium (DMEM) supplemented with 10% fetal bovine serum (FBS). GM03813 SMA patient fibroblasts were obtained from Coriell cell repositories and were grown in minimum essential medium (MEM) supplemented with 1× GlutaMAX and 15% FBS. SH-SY5Y cells were obtained from the ATCC and grown in a 1:1 mix of MEM and F12 growth medium supplemented with 10% FBS. To transfect pre-plated cells, 16–20 h prior to transfection, HeLa cells were counted and seeded at a density of 1 × 10^5^ cells per well of 24-well plates or 4.8 × 10^5^ cells per well of 6-well plates. HEK, SH-SY5Y and NSC34 cells were pre-plated in 24-well plates at densities of 2 × 10^5^, 5 × 10^5^ and 2 × 10^5^ cells per well, respectively. GM03813 cells were plated at a density of 2.8 × 10^5^ cells per well of 6-well plates. Cells were transfected with Lipofectamine 2000 (Life Technologies) following the manufacturer's instructions. Unless otherwise stated, cells were transfected with 0.25 μg of *SMN2^Sup^* for 24-well plates or 1.0 μg for 6-well plates. All transfections were adjusted to 0.5 μg or 2.0 μg total DNA for 24-well plates and 6-well plates, respectively, using empty pCI-Neo vector. For ASO co-transfection with the super minigene, pCI-Neo was replaced with 125 pmol ASO, for a final concentration of 50 nM in the plate. ASOs were synthesized by IDT and contained 2′ methoxyethyl modifications at every position and phosphorothioate backbones. Their sequences are listed in Supplementary Table S1. At 6 h after transfection, medium containing transfection complexes was replaced with fresh medium. To perform reverse transfection, cell suspension containing ∼3 × 10^5^ cells was mixed with DNA:Lipofectamine 2000 complexes formed following the manufacturer's instructions and plated in a well of a 24-well plate. Total DNA for reverse transfections was maintained at 0.8 μg across all samples using pCI-Neo empty vector when needed. For camptothecin (CPT) and cycloheximide (CHX) treatment, medium was replaced with fresh medium containing the indicated concentrations of chemicals at 16 or 18 h after transfection, respectively. At 24 h after transfection, cells were rinsed with 1× phosphate-buffered saline (PBS) and collected for downstream analyses. Cells grown in 6-well plates were collected by scraping in 1 ml of ice-cold 1× PBS, divided based on downstream usage (∼2/3 of cells used for DNA or protein isolation, ∼1/3 for RNA) and collected by centrifuging at 2500 *g* for 2 min at 4°C and removing the supernatant. Cells grown in 24-well plates were lysed directly in TRIzol reagent (Life Technologies) for RNA isolation.

For knockdown of SRSF3 or DHX9 by small interfering RNA (siRNA), 1 × 10^6^ HeLa cells were reverse transfected in 6-well plates with 125 pmol siRNA for a final concentration of 50 nM targeted against the protein of interest or a non-targeting control siRNA using Lipofectamine 2000 following the manufacturer's suggestions. For SRSF3, targeting and control siRNA were obtained from IDT, while for DHX9, siRNAs were obtained from Dharmacon. At 6 h after transfection, cell growth medium containing transfection complexes was replaced with fresh medium. For SRSF3 knockdown, cells were re-plated 24 h after transfection and transfected with 1 μg of the indicated super minigene and 1 μg of pCI-Neo 48 h after initial transfection. For DHX9 knockdown, 48 h after the initial transfection, reverse transfection was repeated under the same conditions and, 24 h later (72 h after initial transfection), cells were reverse transfected with 1 μg of the indicated super minigene and 1 μg of pCI-Neo. Cells were collected by scraping 24 h after the final super minigene transfection as described above.

### DNA/RNA isolation and multi exon skipping detection assay (MESDA)

DNA was isolated from cell pellets using a Qiagen DNeasy blood and tissue kit following the manufacturer's instructions. RNA was isolated using TRIzol reagent (Life Technologies) following the manufacturer's instructions. After isolation, RNA was treated with RQ1 RNase-free DNase (Promega) to remove contaminating DNA, following the manufacturer's instructions. RNA was then re-purified by phenol:chloroform extraction and ethanol precipitation. To generate cDNA, reverse transcription was carried out in 5 μl reactions containing 0.5–1.0 μg of RNA using Superscript III reverse transcriptase (Life Technologies) following the manufacturer's instructions. To generate cDNA for quantitative PCR (qPCR) and MESDA, reverse transcription reactions were primed with random primers (Promega) and the gene-specific primers (E8-Dde and E8-25), respectively. For PCR, reverse transcription reactions were primed with oligo(dT) or 3′-Ex4 gene-specific primer.

MESDA was carried out as previously described (35) using 5′ end-^32^P-labeled reverse primer. For *SMN2^Sup^*MESDA, the forward primer was located in the FLAG tag and the reverse primer was annealed to the first 25 bases of exon 8, while for endogenous MESDA, the forward primer was located in exon 1 across the ATG start codon, which is interrupted by the FLAG tag in *SMN2^Sup^*. For *SMN2^2BM4^*MESDA, the forward primer was the same as in endogenous MESDA, while the reverse primer targeted a specially mutated region of exon 8. Splice isoforms were quantified by densitometric quantification using ImageJ software. For each isoform, percentage inclusion was calculated by dividing the intensity of its band by the total signal in each lane. Accession numbers for major splice isoforms identified by MESDA are listed in Supplementary Table S3.

### Quantitative PCR

qPCR was carried out using PowerUp SYBR green master mix (Life Technologies). Each 20 μl reaction contained 3 μl of 1:40 diluted cDNA (equivalent to 7.5 ng of RNA) or 15 ng of DNeasy-isolated DNA and 0.6 μM of each primer. Templates for standard curves were generated as follows: 10 μg of plasmid containing the sequence of interest was linearized by restriction enzyme, gel purified using Qiaquick gel purification kits (Qiagen) then concentrated by ethanol precipitation. The concentration of the plasmid was then adjusted to 10^10^ molecules per 1.5 μl. A standard curve of 10^2^–10^7^ molecules per 3 μl was then prepared by 10-fold serial dilutions. Copies/cell were estimated using the assumption that each HeLa cell contains ∼30 pg of RNA. For each experiment, *SMN2^Sup^* and endogenous *SMN1/SMN2* transcripts were normalized against hypoxanthine–guanine phosphoribosyltransferase (HPRT) by calculating the relative expression of HPRT in each individual sample relative to the average across a given experiment, then dividing each *SMN2^Sup^* or *SMN* measurement by that value.

### 5′-Rapid amplification of cDNA ends (RACE)

Transcription start sites were identified by RACE using a modified version of the protocol outlined by Scotto-Lavino and colleagues (36). Total RNA was isolated by TRIzol from cells transfected with the super minigene, then treated with RQ1 RNase-free DNase as described above. cDNA was synthesized from 5 μg of total RNA using Superscript III reverse transcriptase (Life Technologies). cDNA was then poly(A) tailed using terminal transferase (New England Biolabs) in the presence of dATP. PCR was carried out using Phusion High-Fidelity DNA polymerase (New England Biolabs) for 30 cycles in the presence of three primers: 5′UTR-RACE, 5RaceUNIV-1 and a gene-specific 3′ primer. Amplified products were separated on native polyacrylamide gels, bands of interest were cut out and DNA was eluted by the ‘crush and soak’ method. The recovered DNA fragments were then cloned into pGEM-T Easy (Promega) vector for sequencing.

### Protein lysate preparation and western blotting

Cells collected by scraping and subsequent centrifugation were resuspended in 60 μl of RIPA buffer (Boston Bioproducts) supplemented with 1× HALT protease inhibitor cocktail (Thermo Scientific). Cells were lysed for 30 min on ice. Samples were centrifuged for 15 min at 12 000 *g* at 4°C to remove cellular debris and the supernatant was transferred to a fresh tube. Protein concentration was measured using Bio-Rad Protein Assay against a standard curve prepared using bovine serum album (Bio-Rad). A 5 μg (for 3×FLAG-SMN) or 20 μg (for all other proteins) aliquot of protein was loaded and run on sodium dodecylsulfate–polyacrylamide gel electrophoresis (SDS–PAGE) gels (6.5% for visualization of DHX9, 10% for all other proteins). Separated proteins were transferred to polyvinylidene difluoride (PVDF) membranes using the Bio-Rad Trans-Blot Turbo transfer system. Membranes were blocked with 5% non-fat milk in 1× TBST (Tris-buffered saline with 0.1% Tween-20) and probed with 1:5000 monoclonal anti-SMN (Clone 8, BD transduction product #610646), 1:2000 polyclonal anti-Actin (Sigma product #A2066), 1:1000 recombinant anti-SRSF3 (abcam #ab198291), 1:100 monoclonal mouse anti-NDHII/DHX9 (Clone E-10, Santa Cruz product #sc-137183), 1:4000 mouse monoclonal anti-Tubulin (Clone DM1A, Sigma product #T6199) or 1:5000 monoclonal anti-FLAG:horseradish peroxidase (HRP) conjugate (Clone M2, Sigma product #A8592). Secondary antibody was 1:5000 goat anti-mouse:HRP conjugate (Jackson ImmunoResearch) for SMN, DHX9 and α-Tubulin, and 1:2000 goat anti-rabbit:HRP conjugate (GE Healthcare) for β-Actin. No secondary antibody was used for 3×FLAG detection since the primary antibody was directly conjugated to HRP. Membranes were developed using Clarity ECL substrate (Bio-Rad) or Femto high sensitivity substrate (Thermo Scientific), and imaged by CCD camera using a UVP ChemStudio Plus (AnalytikJena).

### Analysis of PRO-seq and ChIP-seq data

PRO-seq data (GEO accession no. GSM2692352) and ChIP-seq data (GEO accession nos GSM733682, GSM733684 and GSM733711) generated from HeLa cells (37,38) were downloaded from the NCBI. Reads were visualized against the hg19 human genome annotation using Integrative Genomics Viewer.

## Results

### Construction of an *SMN2* super minigene

**Figure 1.**
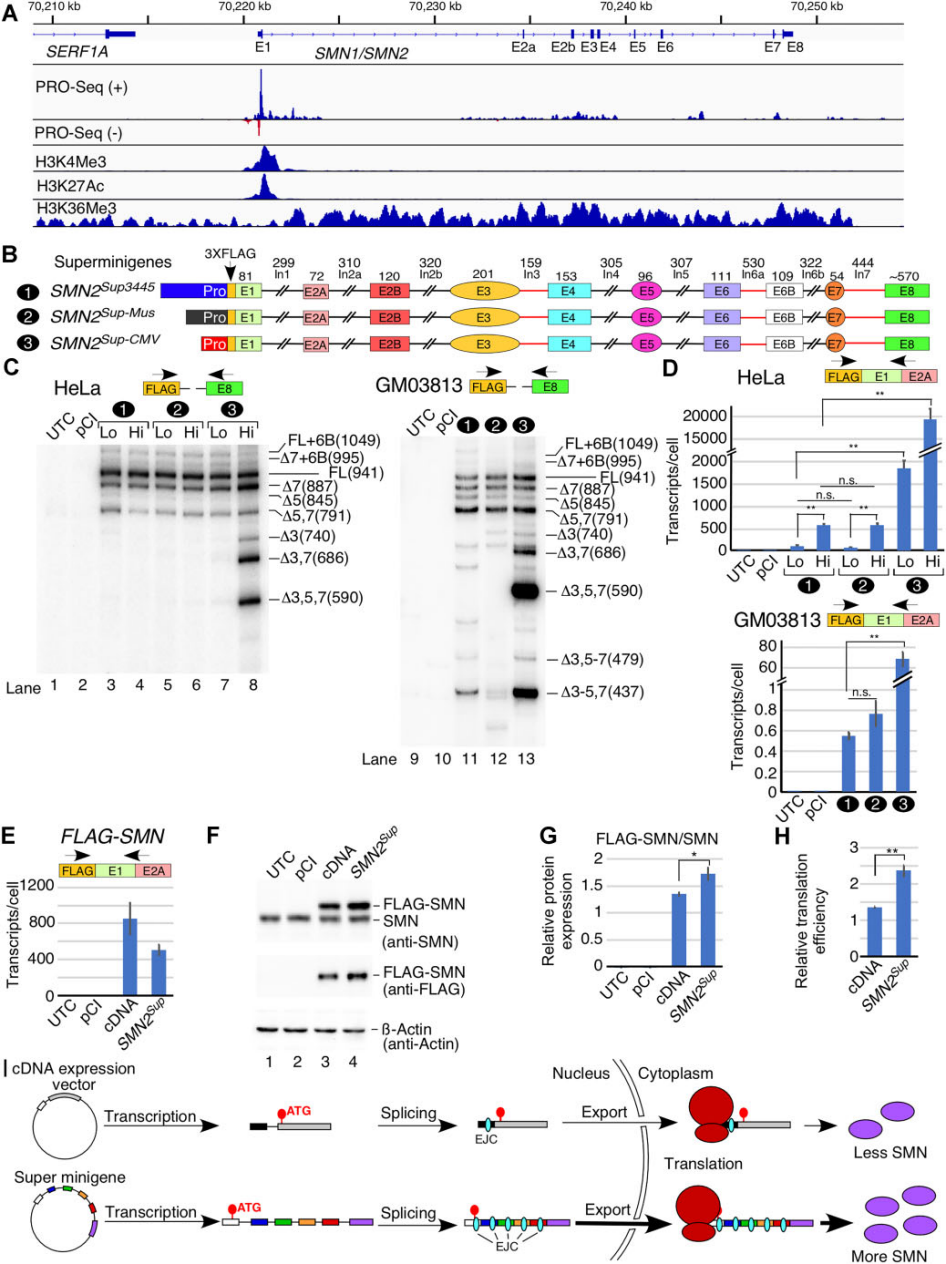
The *SMN2* super minigene is transcribed and spliced efficiently in human cells. (**A**) PRO-seq of the *SMN1/SMN2* locus and ChIP-seq for three histone modifications associated with gene bodies and/or TSSs. The genomic location on chromosome 5 is given at the very top, followed by *SMN* exon and intron locations. PRO-seq is split into positive (blue) and negative (red) strand reads. Bar height indicates the read density at a given genomic location. (**B**) Diagrammatic representation of *SMN2^Sup^* with three different promoters. Exons and promoters are shown as colored shapes, introns with deletions as broken black lines, and full introns as red lines. The size of each exon and intron is indicated. Each construct is assigned a number (indicated on the left), which is then used throughout the figure. (**C**) Left panel: splicing patterns of *SMN2^Sup^* in HeLa cells as determined by MESDA. HeLa cells grown in 6-well plates were transfected with 0.1 μg (Lo) or 1.0 μg (Hi) of the indicated plasmid. Splice isoform identities are labeled at the right side of the gel. Δ indicates exon skipping; +6B indicates inclusion of the cryptic exon 6B. Right panel: splicing pattern of *SMN2^Sup^* in GM03813 SMA patient fibroblasts as determined by MESDA. For all minigenes, 1.0 μg was used to transfect the cells. Locations of primers employed for MESDA are shown above each gel. Abbreviations: UTC, untransfected control; pCI, control transfected with empty pCI-Neo vector alone. (**D**) Upper panel: estimated copies per HeLa cell of *SMN2^Sup^* transcripts measured by qPCR. Lower panel: estimated copies per GM03813 cell of *SMN2^Sup^* transcripts. Locations of primers used are shown above each panel. (**E**) Estimated copies of *FLAG-SMN* transcripts per HeLa cell transfected with pCI-Neo (2 μg), a cDNA expression vector (0.2 μg) or the *SMN2^Sup757^* super minigene (1 μg) as measured by qPCR. Locations of primers used are shown. Abbreviations: cDNA, 3×FLAG-tagged *SMN* cDNA driven by the CMV promoter. (**F**) Representative western blot showing SMN protein expression in HeLa cells transfected with constructs indicated at the top of the image. Protein band identities and antibodies used for membrane probing are shown on the right. (**G**) Quantification of FLAG-SMN signal from the western blot using anti-SMN antibody. Values were calculated by dividing signal for FLAG-SMN by endogenous SMN. (**H**) Translation efficiency estimated by dividing relative protein expression in (G) by RNA expression in (E). (**I**) Model depicting protein expression from a super minigene versus a cDNA expression vector. For all quantifications, *n* = 3. Error bars represent the standard error of the mean (SEM). n.s., *P >* 0.05, *P < 0.05, **P < 0.01.

To gain insight into transcription elongation along the *SMN1*/*SMN2* genes, we analyzed publicly available PRO-seq data that reveal pol II pause sites. Of note, *SMN1* and *SMN2* are indistinguishable by most next-generation sequencing methods, and thus all results are an aggregate of reads derived from *SMN1* and *SMN2*. We also analyzed ChIP-seq data to compare the histone methylation (H3K4Me3 and H3K36Me3) and acetylation (H3K27Ac) profiles along the *SMN* genes. H3K4Me3 and H3K27Ac modifications are associated with transcription initiation, whereas histones within the gene bodies of the actively transcribed genes often contain H3K36Me3 modifications (39,40). Our results revealed clustering of strong pol II pause sites as well as H3K4Me3 and H3K27Ac modifications in the promoter-proximal region (Figure 1A). We also observed clustering of pol II pause sites between exons 2a and 5. Additional discrete pol II pause sites were located within introns 6 and 7. As expected, H3K36Me3 modification was observed throughout the body of the *SMN* genes (Figure 1A). To uncover the role of transcription and splicing in generation of translation-competent full-length *SMN2* transcript, we constructed an *SMN2* super minigene, *SMN2^Sup3445^*, employing a multistep cloning strategy (Figure 1B; Supplementary Figure S1). *SMN2^Sup3445^* harbored ∼3.4 kb of the *SMN2* promoter, all annotated exons and their flanking intronic sequences, as well as the 3′UTR. In most instances, exons in *SMN2^Sup3445^* were flanked by ∼150 nt of their respective intronic sequences, linked by unique restriction enzyme sites at each fragment junction. Due to their small sizes, the entire introns 3 and 7 were used in *SMN2^Sup3445^*. Intron 6 contains a cryptic exon 6B derived from an Alu element (17); therefore, in addition to the 5′ and 3′ ends of intron 6, we also incorporated sequences corresponding to exon 6B and its flanking intronic sequences. To monitor the transcript and protein generated from *SMN2^Sup3445^*, we inserted an in-frame N-terminal 3×FLAG tag immediately downstream of the translation start codon. For the purposes of comparison, we made two additional super minigenes, *SMN2^Sup-Mus^* and *SMN2^Sup-CMV^*, by replacing the *SMN2* promoter with ∼1.3 kb mouse *Smn* promoter and ∼0.7 kb cytomegalovirus (CMV) promoter, respectively (Figure 1B). Much of the mouse *Smn* promoter is distinct from the equivalent human sequence; however, the region surrounding the predicted TSS is highly similar and contains the majority of predicted transcription factor-binding sites (Supplementary Figure S2).

We examined the expression and splicing pattern of transcripts generated from the *SMN2* super minigenes in HeLa cells transfected with low (0.1 μg) and high (1 μg) concentrations of the super minigenes (Figure 1C). We also examined the effect of the super minigenes on the expression and splicing of endogenous *SMN1/SMN2* transcripts (Supplementary Figure S3B). Of note, using a forward primer annealing to either the FLAG sequence or the unique sequence spanning the TSS, we were able to specifically amplify transcripts produced from the super minigenes and endogenous *SMN1/SMN2*, respectively. We estimated the absolute copy number of transcripts generated from both the *SMN2* super minigene and endogenous *SMN1/SMN2*, employing a standard curve generated using known concentrations of the corresponding DNA sequence. We observed ∼20 and ∼30 copies of endogenous *SMN1/SMN2* transcripts in untransfected cells and cells transfected with the empty vector (pCI-Neo), respectively (Supplementary Figure S3B). Cells transfected with low and high concentrations of *SMN2^Sup3445^* produced ∼3- and ∼15-fold more transcripts compared with endogenous *SMN1/SMN2* combined, respectively. While transcript generated from *SMN2^Sup-Mus^* showed similar expression to that of *SMN2^Sup3445^*, we observed ∼60- and ∼600-fold more transcripts produced from the low and high concentrations of *SMN2^Sup-CMV^*, respectively, as compared with transcripts generated from endogenous *SMN1*/*SMN2* combined (Figure 1D).

Transcripts expressed from *SMN2^Sup3445^* and *SMN2^Sup-Mus^* displayed identical splicing patterns that largely recapitulated the splicing pattern of transcripts produced by endogenous *SMN1/SMN2* in HeLa cells (Figure 1C; Supplementary Figure S3B). Both *SMN2^Sup3445^* and *SMN2^Sup-Mus^* produced full-length transcript as the predominant product, followed by exon 7-skipped transcript. The third abundant transcript had both exons 5 and 7 skipped. Among minor splice variants, we detected exon 6B-included transcripts either with or without exon 7. As shown in Figure 1C, the splicing pattern of transcripts generated from *SMN2^Sup3445^* and *SMN2^Sup-Mus^* remained unaffected by a 10-fold increase in the amount of the transfected plasmids (0.1 μg versus 1 μg). Similarly, this increase had no consequence on splicing of endogenous *SMN1/SMN2*. At 0.1 μg, the splicing pattern of transcripts generated from *SMN2^Sup-CMV^* resembled those of *SMN2^Sup3445^* and *SMN2^Sup-Mus^*. However, the pattern changed dramatically when 1 μg of *SMN2^Sup-CMV^* was used for transfection, showing massive skipping of exon 3, as well as an increase in skipping of exons 7 and 5 in different combinations (Figure 1C, lane 8). Transfection with 1 μg of *SMN2^Sup-CMV^* also triggered noticeable skipping of exon 3 in endogenous *SMN1*/*SMN2* transcripts (Supplementary Figure S3B, D).

Unlike HeLa cells that contain both *SMN1* and *SMN2*, GM03813 SMA patient fibroblasts contain only *SMN2* and produce relatively low levels of SMN. We examined the expression of *SMN2* super minigenes in GM03813 cells to determine if low SMN affects splicing of *SMN2* super minigene-derived transcripts. Due to poor transfection efficiency of GM03813 cells, we performed these experiments using 1.0 μg of super minigenes. Compared with HeLa cells, all three super minigenes had much lower expression levels (less than one estimated copy per cell), probably due to poor transfectability of GM03813 cells (Figure 1D). However, their relative expression was similar to what we observed in HeLa cells. In particular, expression from *SMN2^Sup3445^* and from *SMN2^Sup-Mus^* was comparable, while *SMN2^Sup-CMV^* was expressed at a much higher level. In GM03813 cells, the major splice isoform generated from *SMN2^Sup3445^* had exons 5 and 7 skipped together, followed by full-length transcript and transcript with skipped exon 7 (Figure 1C). We also observed some skipping of exon 5 (Figure 1C). In addition, we detected several smaller splice isoforms with skipped exon 3 in different combinations with other exons (Figure 1C). This pattern mostly matched that of endogenous *SMN2* in GM03813 cells (Supplementary Figure S3C), except that endogenous *SMN2* exhibited more skipping of exon 7 and less co-skipping of exons 5 and 7 than *SMN2^Sup^*. The pattern of *SMN2^Sup-Mus^* splicing was similar, except that fewer minor isoforms were detected. In contrast, *SMN2^Sup-CMV^* transcripts underwent massive co-skipping of exons 3, 5 and 7, as well as co-skipping of exons 3, 4, 5 and 7, even more so than in HeLa cells (Figure 1C). However, unlike in HeLa cells, we did not observe any significant change in endogenous *SMN2* splicing in the presence of *SMN2^Sup-CMV^*. We attribute this to low transfection efficiency of GM03813 fibroblasts, in which any potential changes in splicing of endogenous *SMN2* in cells overexpressing *SMN2^Sup-CMV^* are masked by the background of untransfected cells (Supplementary Figure S3C).

In order to test whether *SMN2^Sup^* transcripts are efficiently translated, we transfected HeLa cells with either *SMN2^Sup^* or a cDNA clone expressing FLAG-tagged SMN. Transient expression of *SMN2^Sup^* was driven by the *SMN2* promoter. When we used the same promoter to drive expression of the cDNA construct, the expression levels of *SMN* mRNA and SMN protein were extremely low. Hence, we used the CMV promoter instead with a single vector-derived chimeric intron placed upstream of the start codon. We optimized transfection conditions so that both constructs generated reasonably comparable levels of mRNA (Figure 1E). We then measured transiently expressed SMN by western blot using antibody against SMN or FLAG. Of note, FLAG-tagged SMN generated from either the super minigene or cDNA clone is distinguishable from endogenous SMN due to a higher molecular weight (Figure 1F). We observed that *SMN2^Sup^* produced significantly more FLAG-SMN than the cDNA construct using either anti-SMN or anti-FLAG antibodies (Figure 1F, G), despite a somewhat lower transcript level (Figure 1E). In fact, when we estimated translation efficiency by correcting the relative protein expression by the transcript level, mRNA generated from the super minigene produced ∼1.7-fold more FLAG-SMN than mRNA generated from the cDNA construct (Figure 1H).

### Role of promoter sequences in transcription and splicing of *SMN2*

**Figure 2.**
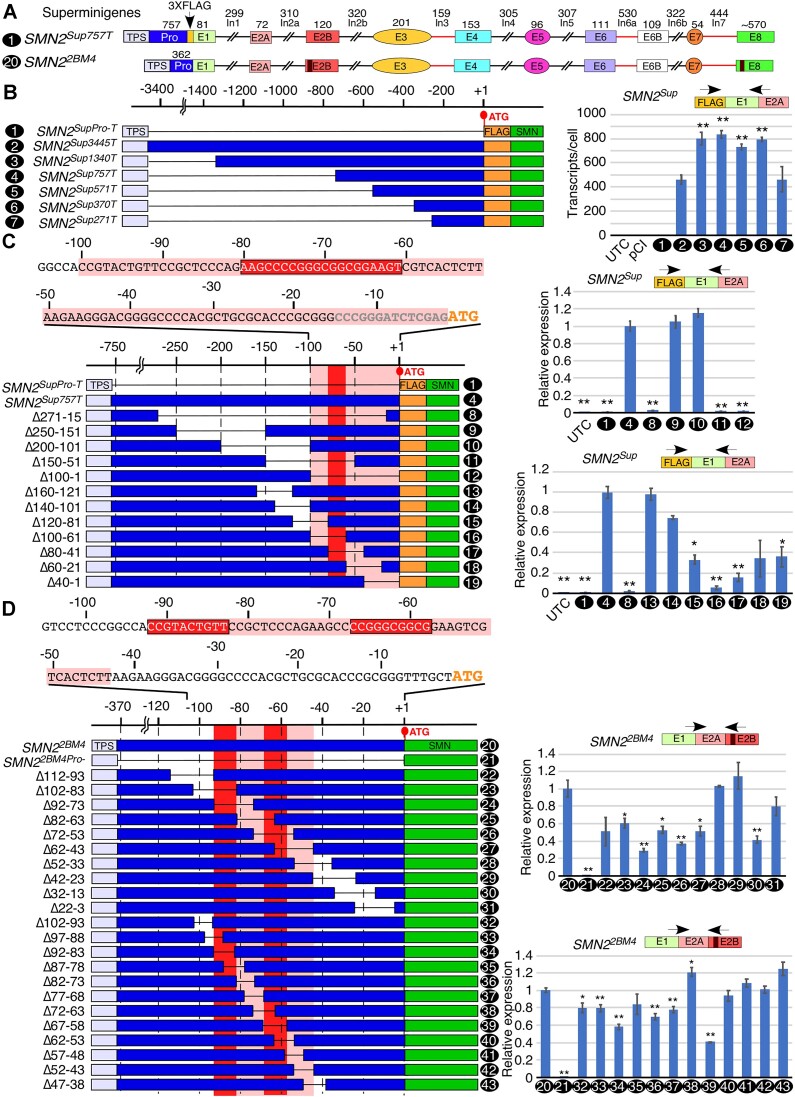
Promoter studies using *SMN2* super minigenes. (**A**) Diagrammatic representation of super minigenes used for this figure. Mutated regions for specific primer binding are indicated with dark red stripes. Other coloring and labeling are the same as in Figure 1B. (**B**) Left panel: diagrammatic representation of promoters in seven super minigene constructs used to determine the minimum size of the *SMN* promoter required for *SMN2^Sup^* transcription. The name/promoter size of each construct are given on the left. Each construct is assigned a number (indicated on the left), which is then used throughout the figure. Important sequence elements are shown as colored boxes. Lines indicate deleted regions within each construct. Numbering is relative to the ATG start codon. Right panel: estimated copies per HeLa cell of *SMN2^Sup^* driven by the indicated promoters. Locations of primers are shown. Abbreviations: UTC, untransfected control; pCI, transfected with empty pCI-Neo vector alone. (**C**) Left panel: overview of deletions made within the 271 nt proximal promoter region of *SMN2^Sup757^*. Pink shaded regions indicate areas where deletions have a significant negative impact on *SMN2^Sup^* expression; the red shaded region indicates the region with the strongest effect. The sequence of the last ∼100 bases of the promoter is shown at the top. Nucleotides in gray indicate restriction enzyme sites inserted for cloning purposes. Numbering is relative to the ATG start codon, which is shown in gold. Right upper panel: estimated copies per HeLa cell of *SMN2^Sup^* driven by promoters with 100 nt deletions. Labeling is the same as in (B). Right lower panel: estimated copies per HeLa cell of *SMN2^Sup^* driven by promoters with 40 nt deletions. (**D**) Left panel: overview of 20 and 10 nt deletions made within the 112 nt proximal promoter region of *SMN2^2BM4^*. Coloring and labeling are the same as in (C). Right panels: estimated copies per HeLa cell of *SMN2^2BM4^* transcripts for deletions indicated in the left panel. For all quantifications, *n* = 3. Error bars represent the SEM. *P < 0.05 compared with *SMN2^Sup3445T^* (B), *SMN2^Sup757T^* (C) or *SMN2^2BM4^* (D), **P < 0.01.

To determine the role of promoter sequences critical for *SMN2* transcription and splicing, we made progressive truncations at the 5′ end of the *SMN2* promoter in the super minigene (Figure 2B). For the sake of clarity, we define sequences upstream of ATG as the promoter. To avoid leaky transcription from an upstream vector-associated promoter or promoter-like sequence, we introduced a TPS consisting of the SV40 poly(A) signal immediately upstream of the promoter insertion site. Of note, the presence of two and four repeats of TPS produced ∼273- and ∼317-fold decreases in the level of transcripts generated from the promoterless super minigene, respectively (Supplementary Figure S4). Hence, we used four repeats of TPS in experiments aimed at examining the effect of deletions of promoter sequences on transcription and splicing. When we tested transcription of *SMN2^Sup^* driven by truncated promoters, we observed an ∼2-fold increase in transcript levels with deletion of ∼2.6 kb of the promoter (Figure 2B). This result is consistent with previous reports showing enhanced transcription upon deletion of this region (27,28). Further truncations that progressively reduced the size of the *SMN2* promoter down to 370 nt had no appreciable effect on transcription (Figure 2B). However, truncation that shortened the *SMN2* promoter to 271 nt reduced the transcription efficiency by half, suggesting the presence of positive regulatory element(s) between 271 and 370 nt upstream of ATG (Figure 2B). Since the larger promoter truncations significantly reduced the size of the super minigene, potentially affecting transfection efficiency, we performed a separate analysis of promoter strength by factoring the amount of the plasmid present within the transfected cells. Towards this, we estimated the concentration of isolated plasmid DNA from the cell using both absolute quantification against a standard curve (Supplementary Figure S5B) and relative quantification against the *SMN* genomic sequence (Supplementary Figure S5C). Adjusting the transcript amount after correcting for the transfection efficiency only moderately changed our results, with the longest and shortest promoters still showing the lowest expression, and the 1340 and 757 nt long promoters showing the highest level of expression (Supplementary Figure S5D). Therefore, we concluded that our promoter deletions did not significantly affect transfection efficiency. MESDA results showed no significant changes in splicing when deletions were introduced in the super minigene promoter (Supplementary Figure S6B).

We introduced a series of deletions in the 757 nt long *SMN2* promoter to examine the role of critical promoter motifs within the 271 nt sequence upstream of ATG (Figure 2C). As expected, a large deletion spanning from 271 to 15 nt upstream of ATG completely abolished transcription (Figure 2C). Overlapping 100 nt deletions in this region delineated the sequence immediately upstream of ATG as indispensable for *SMN2* transcription (Figure 2C). We further narrowed down the location of critical promoter elements by generating a series of 40 nt deletions, each overlapping the last by 20 nt, covering the entire 160 nt immediately upstream of ATG (Figure 2C). Consistent with the results of large deletions, Δ160–121 and Δ140–101 showed no effect on transcription (Figure 2C). In contrast, all deletions within the 100 nt region upstream of ATG reduced transcription by >2-fold, with Δ100–61 and Δ80–41 showing the most severe drop in transcript levels, >5-fold for Δ80–41 and >10-fold for Δ100–61 (Figure 2C). These results support the presence of multiple overlapping positive *cis-*elements within the 100 nt region upstream of ATG. Our results also underscore the role of a strong positive *cis-*element spanning a GC-rich sequence between 61 and 80 nt upstream of ATG (Figure 2C). To capture the effect of promoter deletions on transcription and splicing of super minigene-derived transcripts, we employed MESDA (Supplementary Figure S6D). Overall signal from MESDA varied greatly, consistent with transcript expression levels captured by qPCR. However, splicing of any given exon of *SMN2* was not disproportionately altered by any of the promoter deletions we tested (Supplementary Figure S6D).

Since the majority of promoter elements critical for efficient transcription of *SMN2* appeared to be located in the 100 nt immediately upstream of the ATG start codon, we wanted to eliminate the unintended impact of the FLAG sequence placed next to ATG on transcription of super minigene mutants carrying deletions close to the start codon. Hence, we created a new super minigene without the FLAG tag. In order to distinguish between transcripts produced by the new super minigene and endogenous *SMN1/SMN2* transcripts, we introduced mutations in exon 2B of the *SMN2^Sup370T^* construct, creating a unique site for primer annealing. To avoid affecting the amino acid sequence of a super minigene-derived protein, only the third nucleotide of each codon was changed, in a way that preserved the amino acid sequence. We screened 14 random clones and selected one (*SMN2^2BM4^*) that had a high sequence divergence from the wild type but did not affect splicing of *SMN2^Sup^* (Supplementary Figure S7). In addition, several neutral mutations were introduced in the 3′UTR to create a second unique primer-binding site for MESDA.

We compared the expression of the FLAG-containing construct (*SMN2^Sup757T^*) and *SMN2^2BM4^* carrying 40 nt deletions in the promoter region. Expression of deletion mutants of *SMN2^2BM4^* and of equivalent deletions in *SMN2^Sup757T^* was highly similar (Figure 2C; Supplementary Figure S8). In particular, Δ92–53 and Δ72–33 deletions (equivalent to Δ100–61 and Δ80–41 in *SMN2^Sup757T^*) caused a severe reduction in the transcript level, while Δ112–73 and Δ52–13 deletions reduced the transcript level by about half. We also extended our deletion analysis to the end of exon 1 in order to examine the effect of the promoter-proximal exonic sequences on transcription. Two deletion mutants, Δ32–+8 and Δ12–+28, that eliminated the ATG start codon had ∼40% reduction in transcript levels (Supplementary Figure S8). It is likely that the loss of ATG results in the translation initiation from an alternative start codon, leading to premature termination of translation, which in turn causes transcript degradation by NMD. Alternatively, it is possible that critical *cis-*elements involved in transcription regulation are located in the same region. Supporting the latter hypothesis, Δ+10–+48 mutant encompassing deletion of sequences downstream of the start codon produced ∼30% fewer transcripts (Supplementary Figure S8). Of note, all deletions downstream of ATG maintained the reading frame to generate SMN. Deletions further downstream (mutants Δ+30–+68 and Δ+39–+78) had no significant effect on transcript levels (Supplementary Figure S8C).

In order to localize critical promoter elements spanning the region of interest from 112 to 3 nt upstream of the ATG, we generated a series of super minigenes with 20 nt overlapping deletions (Figure 2D). All deletions from 102 to 43 nt upstream of the ATG significantly reduced expression. In particular, two deletion mutants, Δ92–73 and Δ72–53, showed >60% reductions in transcript levels. These results suggest that the region of interest may encompass multiple promoter elements that are cooperative in nature, since neither deletion was as potent as the larger Δ92–53 deletion. Interestingly, Δ32–13 resulted in a ∼60% reduction in expression, but none of the neighboring deletions significantly affected transcript levels, complicating interpretation. Overlapping 10 nt deletions revealed two mutants, Δ92–83 and Δ67–58, that showed >50% reduction in transcript levels. Several other deletion mutants, including Δ102–93, Δ97–88 and Δ87–78, displayed less drastic reduction in their expression (Figure 2D). Surprisingly, the Δ72–63 mutant showed increased transcription despite the fact that the deletion partially overlapped the positive sequences identified using the Δ67–58 mutant. These results support the existence of overlapping positive and negative regulatory elements spanning the region from 58 to 92 nt upstream of the ATG.

### *SMN2* utilizes an extremely short 5′-untranslated region

**Figure 3.**
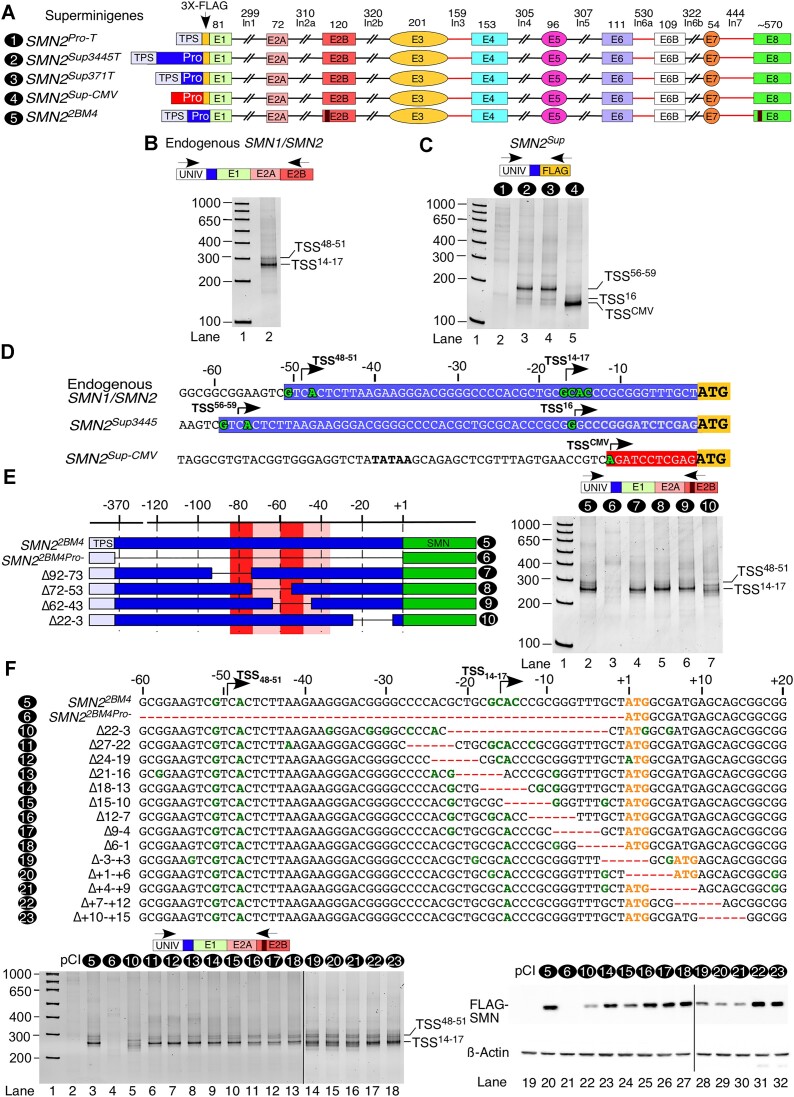
Characterizing the TSSs of *SMN* genes. (**A**) Diagrammatic representation of super minigenes used for this figure. Coloring and labeling are the same as in Figure 2A. (**B**) 5′RACE PCR using a reverse primer specifically targeting endogenous *SMN1/SMN2* in HeLa cells. Locations of primers used for RACE are indicated. UNIV designates a universal adapter sequence added during the first round of PCR. Sizes of the bands in a DNA marker (lane 1) are indicated on the left side. TSSs identified by cloning and sequencing are labeled at the right side. (**C**) 5′RACE PCR using a reverse primer specifically targeting *SMN2^Sup^* expressed from three different super minigenes. Minigene identities are given at the top of the gel. Other labeling is the same as in (B). (**D**) Locations and sequence contexts of the TSSs identified in (B) and (C). Numbering is relative to the ATG start codon highlighted in gold. TSSs are shown in green and labeled with an arrow indicating the direction of transcription. *SMN2* 5′UTRs are boxed in blue; the CMV 5′UTR is boxed in red. In the case of *SMN2^Sup3445^*, nucleotides in gray indicate restriction sites inserted for cloning purposes. (**E**) Characterization of the TSS of the *SMN2^2BM4^* super minigene. Left panel: overview of promoter deletions used for 5′RACE. Right panel: 5′RACE PCR using HeLa cells transfected with the indicated constructs. Labeling is the same as in (B) and (C). (**F**) Characterization of 6 nt overlapping deletions in *SMN2^2BM4^* and *SMN2^2BFLAG^* super minigenes. Top panel: sequences of all deletion mutants in the regions surrounding the TSS. Numbering is relative to the ATG start codon. TSSs identified by sequencing or comparison with known TSS band sizes are shown in green. Deletions are indicated by red dashes. Predicted start codons are shown in gold. Lower left panel: 5′RACE PCR of HeLa cells transfected with the indicated mutants. Labeling is the same as in (B) and (C). Lower right panel: representative western blot showing FLAG-tagged SMN protein expressed in HeLa cells transfected with the super minigenes shown in the top panel.

While multiple TSSs have been reported for *SMN1* and *SMN2*, there is no consensus or independent validations of major TSSs (25). In order to determine the TSS of *SMN1/SMN2* and whether the *SMN2* super minigene recapitulates the properties of the endogenous genes, we performed 5′RACE. We generated cDNA using a reverse primer located in *SMN1/SMN2* exon 2B, followed by poly(A) tailing of the resulting cDNA. We then carried out PCR using an adapter primer that anneals to the poly(A) tail, a universal primer that binds to the adapter sequence and a gene-specific 3′ primer. 5′RACE of endogenous *SMN1/SMN2* using a 3′ primer in exon 2B revealed a major band migrating at ∼270 bases and a minor band migrating at ∼300 bases (Figure 3B). Cloning and sequencing showed that the major TSS is the region 14–17 nt upstream of the ATG start codon, with nucleotides at the 15th and 17th positions being utilized the most (Figure 3B, D; Supplementary Table S4). We refer to it as TSS^14–17^ (Figure 3D). Cloning and sequencing of DNA from the minor band revealed a dual TSS at positions 48 and 51 upstream of ATG, which we refer to as TSS^48–51^ (Figure 3D).

We performed similar 5′RACE using samples prepared from HeLa cells transfected with *SMN2* super minigenes utilizing a 3′ primer located in the FLAG tag to ensure specificity (Figure 3C). As expected, the promoterless control produced no strong bands during amplification, while usage of the CMV promoter to drive *SMN2^Sup^* expression resulted in a very short, 11 nt 5′UTR, consistent with previous reports (Figure 3C) (41). 5′RACE performed on samples transfected with either the full-length *SMN2* super minigene (*SMN2^Sup3445T^*) or a super minigene carrying the minimal promoter (*SMN2^Sup371T^*) generated a primary band revealing transcription initiation at 56 or 59 nt upstream of the ATG (TSS^56–59^) and a minor band corresponding to initiation at 16 nt upstream of ATG (TSS^16^) (Figure 3C, D). These surprising results underscore that the FLAG tag and other sequences introduced for cloning disrupt transcription at TSS^14–17^ and shift the primary transcription initiation site upstream. Hence, we performed all subsequent 5′RACE experiments in the *SMN2^2BM4^* super minigene that lacks the FLAG tag. We used a 3′ primer located in the mutated region of exon 2B to distinguish super minigene transcripts from endogenous transcripts, similar to our approach for qPCR of *SMN2^2BM4^*. To determine whether cell line-specific factors influence TSS choice, we performed 5′RACE in four human cell lines, namely HeLa cells, HEK-293 cells, neuroblastoma SH-SY5Y cells and GM03813 SMA patient fibroblasts. In addition, we also employed mouse motor neuron-like NSC34 cells. As expected, transcription initiation sites used for *SMN2^2BM4^* were similar to that for endogenous *SMN1/SMN2*, with the primary site being TSS^14–17^ and a weaker TSS^48–51^ site (Supplementary Figure S9A). Interestingly, the same TSSs were used when *SMN2^2BM4^* was expressed in mouse cells (NSC34; Supplementary Figure S9A). As shown in Supplementary Figure S9B, *SMN2^2BM4^* largely recapitulated the splicing pattern of endogenous *SMN1/SMN2* in HeLa cells, while in GM03813 cells we observed slightly decreased skipping of exon 7 and increased co-skipping of exons 5 and 7 (Supplementary Figure S9B). *SMN2^2BM4^* expressed in HEK-293 and SH-SY5Y cells showed increased exon 7 skipping compared with the endogenous genes, consistent with the fact that endogenous transcripts are derived from both *SMN1* and *SMN2*.

Having confirmed that the *SMN2^2BM4^* super minigene faithfully recapitulates the TSS of endogenous *SMN1/SMN2*, we next set out to identify promoter regions responsible for TSS selection. We performed 5′RACE using several *SMN2^2BM4^* mutants carrying 20 nt promoter deletions (Figure 3E). These mutants were chosen because the deletion had a significant negative impact on transcription or it overlapped with one of the identified TSSs. Δ92–73 had little to no effect on transcription from TSS^14–17^, although usage of TSS^48–51^ appears to have been reduced or eliminated (Figure 3E). We observed similar results for Δ72–53 and Δ62–43 (Figures 2D and 3E). The Δ22–3 mutant that carried deletion of TSS^14–17^ resulted in selection of multiple weak TSSs without a single primary one, although the level of transcription from this mutant was only moderately reduced (Figures 2D and 3E). Cloning and sequencing of the bands corresponding to these weak TSSs revealed a range of sites both upstream and downstream of the deletion, including some located downstream of the ATG start codon (Figure 3F; Supplementary Table S4).

In order to further narrow down the sequences responsible for initiating transcription at TSS^14–17^, we generated a series of 6 nt deletions covering the region from 27 nt upstream to 15 nt downstream of the ATG start codon (Figure 3F). Notice that two deletions, Δ+1–+6 and Δ+4–+9, created identical sequences (Figure 3F). However, for presentation purposes, we included both clones separately. 5′RACE performed on cells transfected with wild-type *SMN2^2BM4^*, the promoterless control and Δ22–3 replicated previous results. In contrast to Δ22–3, all of the promoter deletions upstream of the ATG had little effect on the TSS selection, even Δ18–13 in which TSS^14–17^ was completely deleted (Figure 3F). The only observable effect was a slight weakening of the TSS^14–17^ band in the last three deletions upstream of the ATG (Δ12–7, Δ9–4 and Δ6–1) (Figure 3F). These deletions also had no discernible effect on *SMN2^Sup^* total transcript level or pre-mRNA splicing (Supplementary Figure S10; data not shown). Deletions that overlapped the first 6 nt of the coding sequence (Δ–3–+3, Δ+1–+6/Δ+4–+9) all caused a broadening of the primary TSS band, indicating usage of additional downstream TSSs, and a moderate reduction in transcript levels (Figure 3F; Supplementary Figure S10). This suggests that factors that bind near the ATG play a critical role in TSS selection, possibly linking transcription and translation regulation.

To identify TSSs utilized in the super minigene mutants, we sequenced 6–10 clones for each cloned band generated by 5′RACE (Supplementary Table S4). Δ27–22 and Δ24–19 had little effect on the TSS selection, with most clones pointing to usage of TSS^14–17^. However, these deletions may result in some loss of fidelity, since we identified one clone (Δ27–22 mutant) in which a nucleotide located at the 12th position upstream of ATG was used as a TSS, and one clone (Δ24–19 mutant) in which A of the ATG start codon was utilized as a TSS. In Δ21–16, Δ18–13 and Δ15–10 mutants, in which the TSS^14–17^ sites were partially or completely lost, transcription occurred at positions –9 and –22. In Δ12–7, Δ9–4 and Δ6–1 mutants, the TSS^14–17^ site was used, with some deviations (Supplementary Table S4). In Δ–3–+3 and Δ+1–+6/Δ+4–+9 mutants, TSS^14–17^ as well as a site located downstream of the ATG were used (Supplementary Table S4). Interestingly, we identified a few clones encompassing promoter deletions in which transcription initiation occurred at different positions within the 5′ portion of intron 1. This is consistent with weakening, but not complete loss, of the primary TSS^14–17^.

To determine whether shortening of the 5′UTR or disruption of the TSS might have an effect on super minigene translation, we generated constructs in which a single copy of the FLAG tag was placed in exon 2B of the wild-type *SMN2^2BM4^* and its promoter deletion mutants shown in Figure 3F. The goal was to move the epitope away from the TSS and use the smallest tag possible in order to avoid disrupting the local sequence context. We chose a region of exon 2B with low sequence conservation in SMN derived from various organisms to reduce the chance of disrupting important protein features. The super minigene produced a robust signal when anti-FLAG antibody was used for probing (Figure 3F, lane 20), while no signal was observed in empty vector and promotorless controls (Figure 3F, lanes 19 and 21). In Δ22–3, mutant protein expression was greatly reduced, as expected, since the 5′UTR sequence is almost completely different as compared with the wild-type construct (Figure 3F). Of the other 5′UTR mutants we tested, Δ18–13 displayed slightly reduced protein expression; in Δ15–10, expression was decreased by about half; and in Δ12–7, Δ9–4 and Δ6–1, protein expression was unchanged (Figure 3F). These results would suggest that the 5′-most portion of the 5′UTR has an impact on SMN translation, while the 10 nt long region immediately upstream of ATG is not important. We also tested translation of mutants with deletions made in the vicinity of ATG. Interestingly, although Δ–3–+3 and Δ+1–+6/Δ+4–+9 mutants all retained ATG at the same position relative to TSS^14–17^, their translation was greatly diminished (Figure 3F). It is not clear how much of this can be attributed to transcription initiation downstream of the ATG start codon and how much to disruption of ATG sequence context. Importantly, the Δ+7–+12 mutant exhibited normal translation, so it is clear that the second ATG codon is not required for efficient translation.

### Skipping of *SMN2* exon 3 in overexpressed transcripts is associated with downstream splicing events

**Figure 4.**
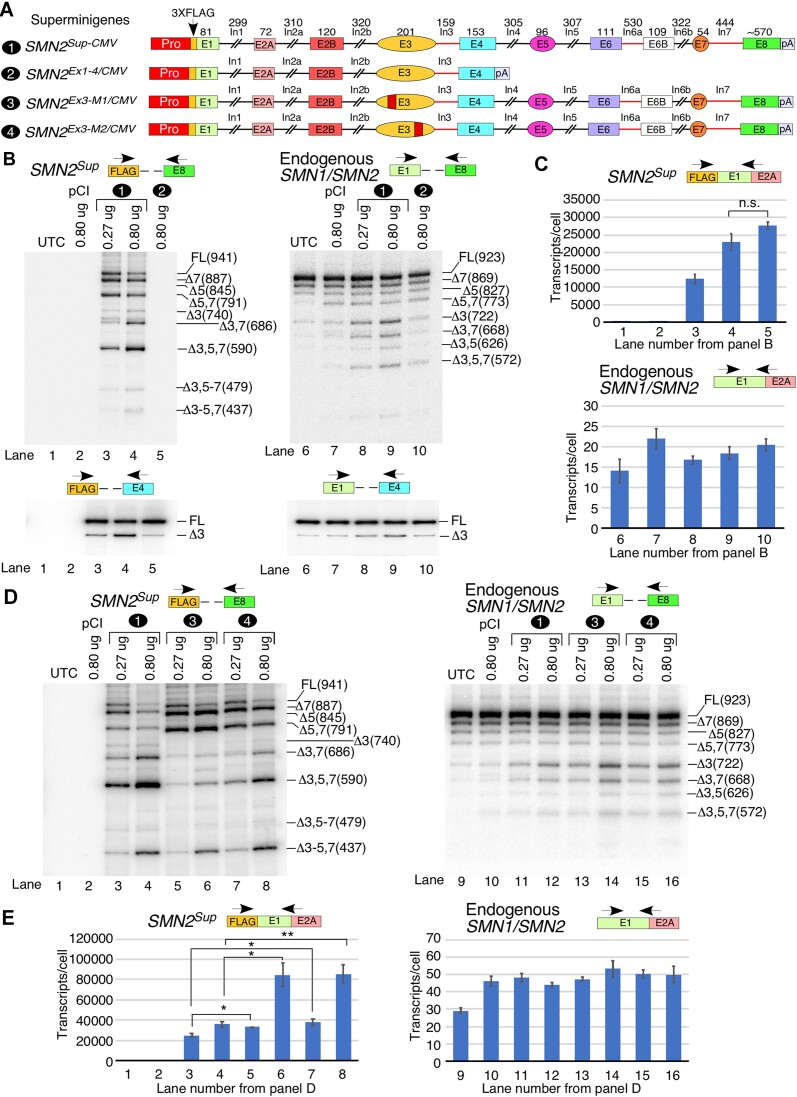
Strain on the transcription and splicing machinery leads to exon 3 skipping. (**A**) Diagrammatic representation of overexpression constructs used for this figure. Mutated portions of exon 3 are indicated with red stripes. Other coloring and labeling are the same as in Figure 1B. (**B**) Splicing of *SMN2^Sup^* (left panels) and endogenous *SMN1/SMN2* (right panels) in HeLa cells transfected with *SMN2^Sup-CMV^* or *SMN2^Ex1-4/CMV^*. Top panels: MESDA examining splicing of all internal exons. The transfected plasmid and quantity are indicated at the top of each gel image. Splice isoforms are indicated at the right. Lower panels: splicing of exon 3 as measured by amplification of the region from exon 1 to exon 4. Locations of primers are indicated above each panel. (**C**) Estimated copies per HeLa cell of *SMN2^Sup^/SMN2^Ex1-4^* (upper panel) and endogenous *SMN1/SMN2* (lower panel) as determined by qPCR. Locations of primers used are shown. (**D**) Splicing patterns of *SMN2^Sup^* (left panel) and endogenous *SMN1/SMN2* (right panel) in HeLa cells transfected with *SMN2^Sup-CMV^* or *SMN2^Sup^* constructs with mutations in exon 3, as determined by MESDA. Labeling is similar to (B). (**E**) Estimated copies of *SMN2^Sup^* (left panel) and endogenous *SMN1/SMN2* (right panel) transcripts per HeLa cell transfected with *SMN2^Sup-CMV^* or *SMN2^Sup^* with mutations in exon 3 that affect pre-mRNA splicing. For all quantifications, *n* = 3. Error bars indicate the SEM. n.s., *P* > 0.05, *P < 0.05, **P < 0.01.

Our analysis of PRO-seq data revealed numerous pol II pause sites in the vicinity of exon 3 (Supplementary Figure S11A). To determine whether the enhanced skipping of *SMN2* exon 3 in the transcripts overexpressed from *SMN2^Sup-CMV^* is primarily associated with *cis-*elements within exon 3 and/or the promoter or whether downstream sequences are required as well, we generated an *SMN2* minigene, *SMN2^Ex1-4/CMV^*, by removing sequences downstream of exon 4 from *SMN2^Sup-CMV^* (Figure 4A). We then compared the splicing patterns of transcripts overexpressed from *SMN2^Sup-CMV^* and *SMN2^Ex1-4/CMV^* (Figure 4B; Supplementary Figure S11). To capture alternative splicing of *SMN2^Ex1-4/CMV^*, in addition to MESDA we performed reverse transcription–PCR (RT–PCR) where the reverse primer was located in exon 4 (Figure 4B, lower panels). As we previously observed (Figure 1B), overexpression of transcripts from a large quantity of *SMN2^Sup-CMV^* promoted skipping of exon 3 and co-skipping of exons 3 and 7 with or without exon 5. The major splice isoform produced from *SMN2^Sup-CMV^* transfected in high amounts (0.8 μg) lacked exons 3, 5 and 7 (Figure 4B, lane 4; Supplementary Figure S11). At the same plasmid concentration, *SMN2^Ex1-4/CMV^* produced a similar level of transcripts to *SMN2^Sup-CMV^* (Figure 4C). Yet, transcripts generated from *SMN2^Ex1-4/CMV^* exhibited only low levels of exon 3 skipping. Considering that transcription from both *SMN2^Sup-CMV^* and *SMN2^Ex1-4/CMV^* was driven by the same CMV promoter, our results ruled out the role of the promoter-associated factors in skipping of *SMN2* exon 3. We next examined the effect of overexpression of transcripts from *SMN2^Sup-CMV^* and *SMN2^Ex1-4/CMV^* on splicing of endogenous *SMN1/SMN2* (Figure 4B; Supplementary Figure S11). We detected no appreciable effect of overexpression of transcripts from *SMN2^Ex1-4/CMV^*. However, overexpression of *SMN2^Sup-CMV^* promoted noticeable skipping of exon 3 in endogenous *SMN1/SMN2*, alone or together with exon 7 and/or exon 5. These results suggested that the overexpression of *SMN2^Sup-CMV^* might deplete common factors associated with the splicing of exons 3, 5 and 7 in the context of the full-length *SMN* transcripts, and that the presence of all exons and introns of *SMN* is necessary for this effect. Alternatively, overexpression of SMN protein may be the cause of exon 3 skipping. Cloning and sequencing of the bands corresponding to exon 5 and 7 co-skipping, 3 and 7 co-skipping and 3, 5 and 7 co-skipping revealed that they were almost entirely comprised of *SMN2* sequences (Supplementary Figure S12), while *SMN1* was the primary source of the isoform in which exon 3 alone was skipped. This would suggest that exon 3 is skipped in both *SMN1* and *SMN2* transcripts in roughly equal proportions, while exon 7, as expected, is skipped primarily in *SMN2*, alone or together with exons 3 and/or 5. Exon 5 appeared to be primarily skipped in *SMN2*, although its overall rate of skipping is much less than that of exons 3 and 7, complicating interpretation.

To test the potential involvement of *cis-*elements within exon 3 in its own skipping under the conditions of high levels of *SMN2^Sup-CMV^* overexpression, we generated super minigenes *SMN2^Ex3-M1/CMV^* and *SMN2^Ex3-M2/CMV^* carrying translationally silent mutations in two different regions of exon 3 (Figure 4A; Supplementary Figure S11B). Despite considerably higher expression levels as compared with *SMN2^Sup-CMV^*, the overexpressed transcripts produced by *SMN2^Ex3-M1/CMV^* and *SMN2^Ex3-M2/CMV^* showed reduced skipping of exon 3 (Figure 4D, E; Supplementary Figure S11E). At the same time, similar to overexpressed transcripts generated from *SMN2^Sup-CMV^*, overexpressed transcripts produced by *SMN2^Ex3-M1/CMV^* and *SMN2^Ex3-M2/CMV^* showed increased skipping of exon 5 and 7 as compared with the wild-type *SMN2* super minigene driven by the *SMN* promoter (compare Figure 4D with Figure 1B). These results suggested that mutations in exon 3 of the super minigenes could stimulate efficient splicing of exon 3 but had no effect on exons 5 and 7. Interestingly, overexpression of *SMN2^Ex3-M1/CMV^* and *SMN2^Ex3-M2/CMV^* showed even more enhanced skipping of exon 3 in transcripts generated from endogenous *SMN1/SMN2* than *SMN2^Sup-CMV^* (Figure 4D; Supplementary Figure S11), consistent with their increased transcript levels. These findings underscored that factor(s) generated from or impacted by the overexpressed *SMN2* transcripts affect splicing of endogenous *SMN1*/*SMN2*.

### Effect of SMN and SMNΔ7 overexpression on splicing of endogenous *SMN1*/*SMN2*

**Figure 5.**
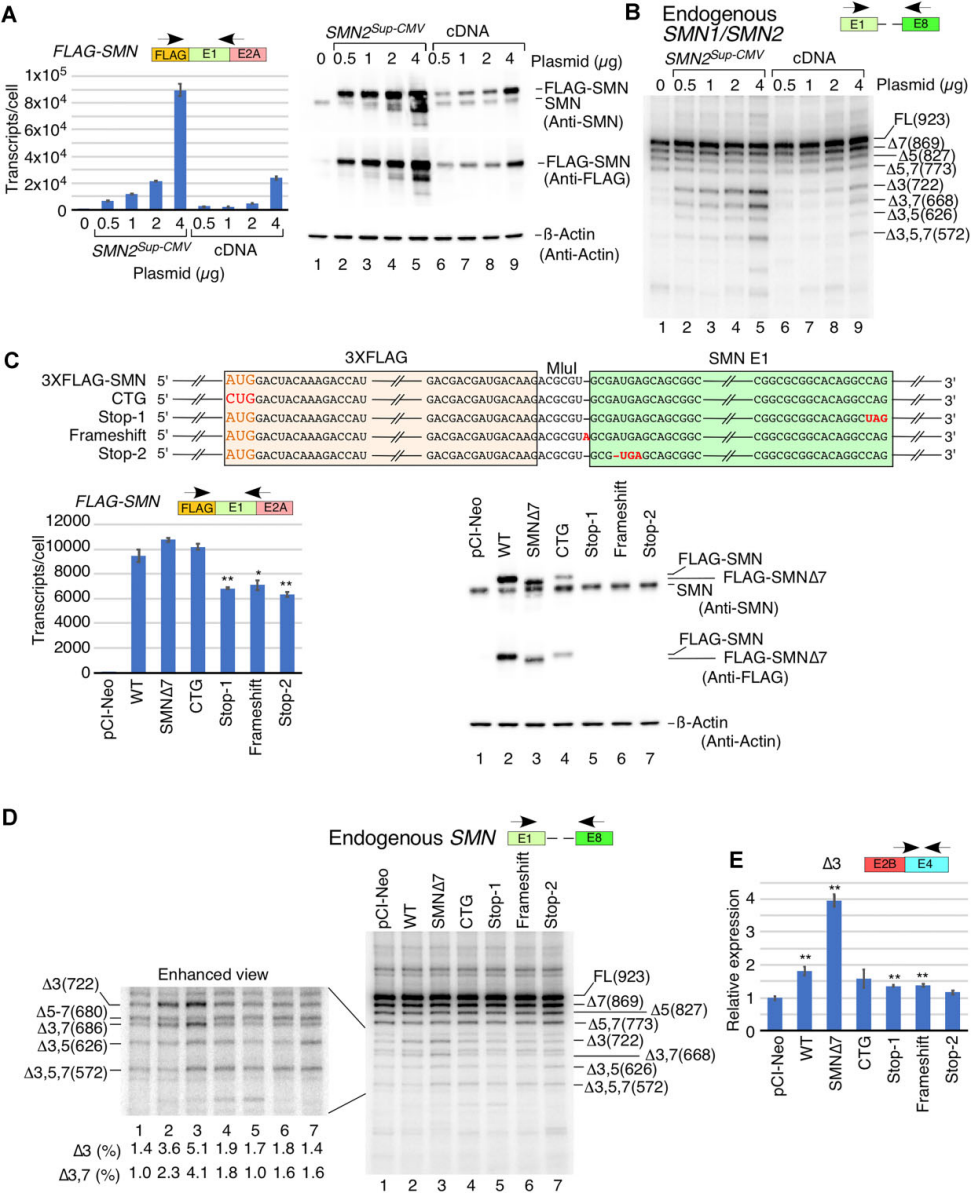
Effect of SMN and SMNΔ7 overexpression on splicing of endogenous *SMN1*/*SMN2*. (**A**) Left panel: estimated copy number of FLAG-tagged transcripts per HeLa cell transfected with different amounts of *SMN2^Sup-CMV^* or SMN cDNA expression vector driven by the CMV promoter. Labeling is the same as in Figure 1D. Right panel: representative western blot showing expression of 3×FLAG-tagged SMN protein in HeLa cells transfected with constructs indicated at the top of the image. Protein band identities and antibodies used for membrane probing are shown on the right. (**B**) Splicing of endogenous *SMN1/SMN2* in transfected HeLa cells as determined by MESDA. Labeling is the same as in Figure 1C. (**C**) Overexpression of different *SMN* constructs. Top panel: overview of expression constructs used, including the wild-type SMN expression vector and four mutants designed to prevent SMN protein expression. The 3×FLAG tag and exon 1 are shown as colored boxes with their partial sequences given. Mutated bases and novel stop codons are shown in red. Lower left panel: estimated copies of FLAG-tagged *SMN* transcripts per HeLa cell transfected with various constructs indicated at the bottom of the panel. Labeling is the same as in Figure 1D. Lower right panel: representative western blots showing protein expression. Labeling is the same as in (A). (**D**) Splicing of endogenous *SMN1/SMN2* in HeLa cells transfected with the indicated expression vectors, as determined by MESDA. An enlargement (with longer exposure) of the image portion showing *SMN* splice isoforms from Δ3 to Δ3,5,7 is presented on the left. The percentage of exon 3 skipping and exon 3 and 7 co-skipping is indicated below. Other labeling is the same as in Figure 1C. (**E**) Relative expression of endogenous *SMN1/SMN2* splice isoforms that lack exon 3 in HeLa cells transfected with the indicated plasmids as determined by qPCR using an exon 2B/4 junction primer. Labeling is the same as in Figure 1D. For all quantifications, *n* = 3. Error bars indicate the SEM. *P < 0.05, **P < 0.01.

We interrogated whether the effect of CMV-driven *SMN2* super minigene overexpression on splicing of endogenous *SMN1*/*SMN2* is due to overexpressed proteins (SMN and SMNΔ7). We began by comparing the effects of different amounts of the CMV promoter-driven *SMN2* super minigene (*SMN2*^Sup-CMV^) and a cDNA-based SMN expression vector on endogenous *SMN1*/*SMN2* splicing. At any given plasmid amount, we observed more transcripts generated from the super minigene than from the cDNA clone (Figure 5A), supporting the hypothesis that mRNAs generated from intron-containing genes are more stable than those generated from intronless genes (42). Consistently, more SMN was expressed from the *SMN2* super minigene than from the cDNA clone at any given plasmid amount (Figure 5A). Noticeably, we captured enhanced skipping of exon 3 in endogenous *SMN1*/*SMN2* transcripts in samples transfected with the highest amount of cDNA-based SMN expression vector (Figure 5B; Supplementary Figure S13A). The effect was similar to the one produced by *SMN2^Sup-CMV^* under the condition of comparable protein expression (Figure 5A, B, lanes 2 and 9). We next examined if SMNΔ7 generated from a cDNA-based expression vector could trigger skipping of endogenous *SMN1*/*SMN2* exon 3 as well. Notice that despite comparable transcript levels, the amount of SMNΔ7 produced was lower compared with SMN (Figure 5C). This is likely to be due to the poor stability of SMNΔ7 (9). Nonetheless, SMNΔ7 had an even stronger effect on skipping of endogenous exon 3 than SMN (Figure 5D, E).

To determine whether overexpressed transcripts or their corresponding translated products are the cause of enhanced skipping of exon 3 of endogenous *SMN1*/*SMN2*, we generated four additional cDNA-based SMN expression vectors. These vectors carried mutations that prevented SMN production by mutating the start codon to CTG (CTG mutant), by introducing a stop codon at the end of exon 1 (Stop-1 mutant), by causing a frameshift due to an extra nucleotide (Frameshift mutant) and by introducing a stop codon at the start of exon 1 (Stop-2 mutant) (Figure 5C). As shown in Figure 5C, transcript levels produced from these mutated expression vectors were somewhat comparable, granted Stop-1, Frameshift and Stop-2 constructs generated less mRNA (Figure 5C). Except for the CTG mutant that produced very low levels of SMN, no FLAG-tagged SMN protein was detected in cells transfected with the mutated expression vectors (Figure 5C). Overexpression of any of these mutated constructs did not cause changes in the splicing of endogenous *SMN1*/*SMN2*. (Figure 5D; Supplementary Figure S13B). These results confirmed that overexpression of SMN and/or its truncated variant(s) is the cause of the enhanced skipping of exon 3 in endogenous *SMN1*/*SMN2* transcripts.

### Effect of transcription elongation inhibition on splicing of *SMN2*

**Figure 6.**
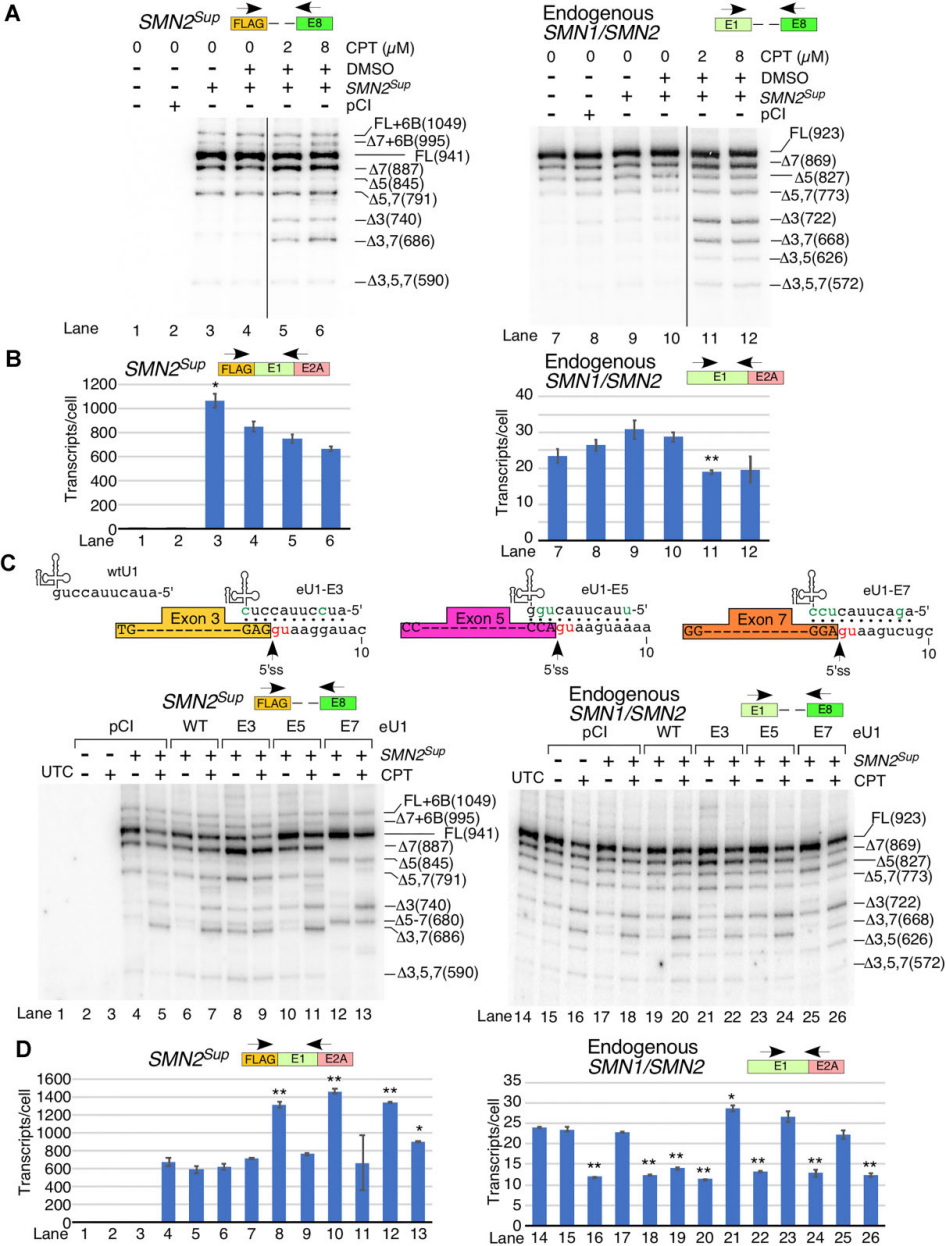
Use of *SMN2^Sup^* to study transcription-coupled splicing regulation. (**A**) Splicing patterns of *SMN2^Sup^* (left panel) and endogenous *SMN1/SMN2* (right panel) in HeLa cells treated with two different concentrations of CPT, as determined by MESDA. Treatments are indicated at the top of each gel; splice isoforms are labeled at the right. Locations of primers used for MESDA are shown. (**B**) Estimated copy number of *SMN2^Sup^* (left panel) and endogenous *SMN1/SMN2* (right panel) per transfected and CPT-treated HeLa cell. Samples are the same as in (A). Locations of primers used for qPCR are shown. (**C**) Top panel: diagrammatic representation of base pairing between engineered U1 snRNAs (eU1s) and 5′ss. Sequence contexts of each GU (in red) are shown. Base pairing is marked by black dots; mutated nucleotides within U1 snRNAs are in green. The 5′ss are indicated by arrows. Lower panels: splicing patterns of *SMN2^Sup^* (left panel) and endogenous *SMN1/SMN2* (right panel) in HeLa cells transfected with eU1 overexpression vectors and treated with CPT, as determined by MESDA. The types of transfections and CPT treatments are specified at the top of each gel. Splice isoforms are indicated on the right side of each gel. Locations of primers used for MESDA are shown. (**D**) Estimated copy number of *SMN2^Sup^* (left panel) and endogenous *SMN1/SMN2* (right panel) transcripts per transfected and CPT-treated HeLa cell. Samples are the same as in (C). Locations of primers used for qPCR are shown. For all quantifications, *n* = 3. Error bars indicate the SEM. *P < 0.05, **P < 0.01.

CPT inhibits DNA topoisomerase I activity, which interferes with the ability of pol II to unwind and transcribe DNA, and serves as a valuable reagent for transcription elongation studies (43,44). We examined the effect of CPT on splicing of transcripts generated from *SMN2^Sup^* and endogenous *SMN1/SMN2*. The goal was to determine if the super minigene recapitulates the transcription-coupled splicing regulation of endogenous *SMN1/SMN2*. It has been recently shown that *SMN2* exon 7 is a class II exon, meaning its inclusion is stimulated by fast pol II and inhibited by slow pol II (32,44). To independently validate this finding and also uncover how *SMN1/SMN2* exons 3 and 5 respond to the manipulation of pol II elongation rates, we transfected HeLa cells with *SMN2^Sup^* and treated cells at 24 h after transfection with low and high concentrations of CPT for 8 h. We selected the dosages and duration of treatment based on titration results generated using a range of CPT concentrations reported in the literature (Supplementary Figure S14A). Importantly, the ‘high’ concentration of CPT we subsequently selected for our experiments did not stress the cells. Cloning and sequencing revealed that all isoforms that incorporated exon 7 skipping (Δ7, Δ5,7, Δ3,7 and Δ3,5,7) were composed of only *SMN2* sequences, while isoforms with exon 7 inclusion (Δ5, Δ3 and Δ3,5) were composed of only *SMN1* sequences (Supplementary Figure S14B). This implies that only *SMN2* exon 7 is sensitive to CPT treatment, while exon 3 from both *SMN1* and *SMN2* is sensitive. Both CPT doses caused a decrease in levels of the full-length transcripts and an increase in exon 7 skipping in the case of *SMN2^Sup^* as well as endogenous *SMN1/SMN2* (Figure 6A; Supplementary Figure S15A). In addition, CPT increased skipping of exon 3, alone or together with exon 7, for both *SMN2^Sup^* and endogenous *SMN1/SMN2* (Figure 6A; Supplementary Figure S15A). However, CPT-induced skipping of exon 3 was more pronounced in transcripts generated from endogenous *SMN1/SMN2*. Aside from co-skipping with other exons, exon 5 skipping appears unaffected by CPT treatment in both endogenous *SMN1/SMN2* and *SMN2^Sup^*. Transcript levels of endogenous *SMN1/SMN2* and *SMN2^Sup^* appeared slightly lower but did not reach statistical significance (Figure 6B).

U1 snRNP defines the 5′ss, largely through base pairing of U1 snRNA with the 5′ss of the pre-mRNA. Depletion of U1 snRNA has been found to promote skipping of internal *SMN* exons in different combinations (45). Engineered U1 snRNAs (eU1s) that target the 5′ss of *SMN2* exon 7 or downstream intronic sequences prevent skipping of *SMN2* exon 7 (45–47). It has also been observed that a strong RNA:RNA duplex formed between eU1s targeting the 5′ss of exon 7 as well as downstream sequences can promote intron 7 retention (45). To determine if skipping of exons caused by CPT could be prevented by overexpression of eU1s, we transfected HeLa cells with *SMN2^Sup^* along with eU1s targeting the 5′ss of the skipped exons (exons 3, 5 or 7) (Figure 6C). Twenty-four hours later, we treated the cells with 8 μM CPT for 8 h and then analyzed transcription and splicing. Expression of all three eU1s triggered an increase in transcript levels of *SMN2^Sup^*, which was reversed by CPT treatment (Figure 6D). In the case of endogenous *SMN1/SMN2*, the transcript level increase in response to eU1 overexpression was more modest; here too transcript levels were decreased by CPT (Figure 6D). eU1s targeting exons 3, 5 and 7 elicited varied responses on splicing of their respective exons in the presence of CPT (Figure 6C; Supplementary Figure S15B). For instance, eU1 targeting the 5′ss of exon 3 partially suppressed exon 3 skipping induced by CPT, while also enhancing CPT-induced skipping of exon 7. We observed a noticeable decrease in co-skipping of exons 5 and 7 in samples in which eU1 targeting the 5′ss of exon 5 was overexpressed, although the effect was subtle due to a very low level of exon 5 skipping. We captured a strong stimulatory effect of eU1 targeting the 5′ss of exon 7 on its splicing. This eU1 also fully suppressed the ability of CPT to promote exon 7 skipping in transcripts generated from both *SMN2^Sup^* and endogenous *SMN2*. At the same time, this eU1 was unable to prevent the adverse effect of CPT on upstream splicing events such as skipping of exon 3 (Figure 6C; Supplementary Figure S15B).

### Role of the first intron in the splicing regulation of downstream exons

**Figure 7.**
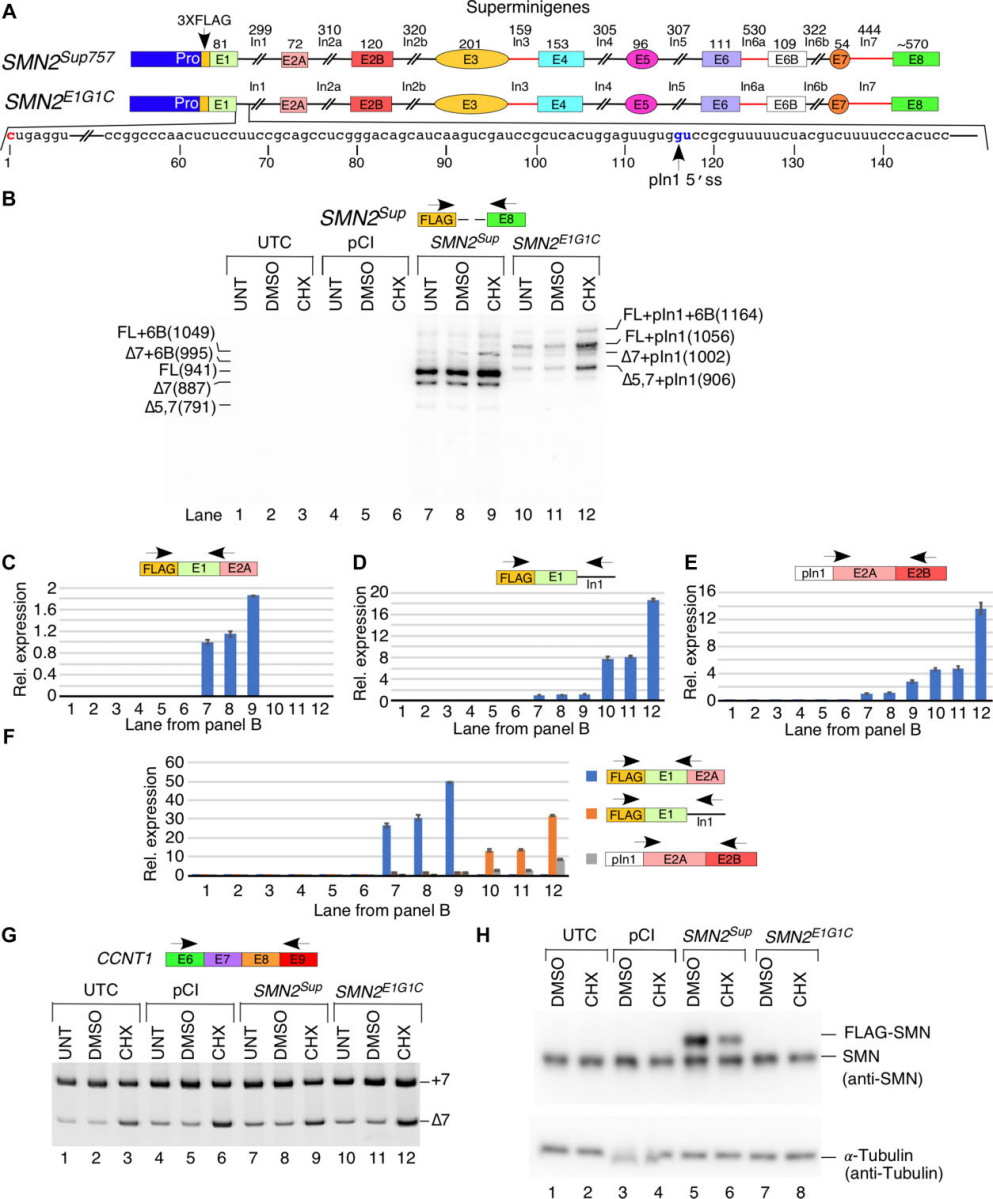
Effect of intron 1 removal or retention on downstream splicing events. (**A**) Diagrammatic representation of *SMN2^Sup757^* and the *SMN2^E1G1C^* mutant. Coloring and labeling are the same as in Figure 1B. The sequence of the 5′-most region of intron 1 is shown. The G1C mutation is shown in red and a cryptic 5′ss activated in the presence of this mutation is indicated in blue. Numbering is relative to the start of intron 1. Abbreviations: pIn1, partial intron 1. (**B**) Splicing pattern of *SMN2^Sup^* in HeLa cells transfected with *SMN2^Sup757^* or *SMN2^E1G1C^* followed by treatment with CHX for 6 h, as determined by MESDA. Plasmids used for transfection and the type of drug treatments are indicated at the top of the gel. Splice isoforms are marked on both sides of the gel. The locations of primers used for MESDA are shown. Abbreviations: UTC, untransfected control; UNT, untreated control; CHX, 20 μg/ml cycloheximide. Labeling is the same as Figure 1C. (**C**) Relative expression of *SMN2^Sup^* transcripts with canonical splicing of intron 1 as determined by qPCR. The location of primers used is indicated. (**D**) Relative expression of *SMN2^Sup^* transcripts containing at least some sequences from intron 1 as determined by qPCR. (**E**) Relative expression of transcripts that incorporate the pI1 5′ss as determined by qPCR. (**F**) Relative expression of all transcripts from (C–E) compared directly with each other. Locations of primers and color-coding for bars are given at the right side of the graph. (**G**) RT–PCR results for *Cyclin T1* exon 7 splicing to confirm effects of CHX are shown. Annealing positions of primers used for PCR are given in the top panel. Other labeling is the same as in (B). (**H**) Representative western blot showing expression of endogenous and *SMN2^Sup^*-derived SMN protein in transfected and CHX-treated HeLa cells. The identity of constructs used for transfection as well as the type of drug treatments are indicated at the top of the gel. Protein band identities and antibodies used for membrane probing are shown at the right.

The first intron of *SMN* is the longest intron and harbors multiple repeat elements, including 22 Alu-like sequences. Shortening of intron 1 to 299 nt, leading to the loss of all intronic repeat elements, did not have an adverse effect on its removal. As a proof-of-concept that we can alter *SMN2^Sup^* splicing by targeted mutations, we generated a mutant *SMN2* super minigene, *SMN2^SupG1C^*, in which the invariant G residue at the first position of intron 1 was substituted with a C residue (G1C substitution) (Figure 7A). As expected, G1C substitution completely eliminated the usage of the canonical 5′ss of intron 1 (Figure 7B, C). Instead, we detected the activation of a downstream cryptic 5′ss at intronic position 117. Transcripts generated using this splice site contain part of intron 1, so we dubbed them partial intron 1, or pIn1 for short. The usage of the cryptic 5′ss in intron 1 was often paired with co-skipping of exons 5 and 7 (Figure 7B, lanes 10–12; Supplementary Figure S16B), but did not drastically affect splicing of other exons. Due to the presence of a premature stop codon (PTC), intron 1-retained transcripts are likely to be subjected to NMD. Consistently, the overall intensities of bands corresponding to *SMN2^Sup^* splice isoforms were much lower in the case of *SMN2^SupG1C^*-transfected samples (Figure 7B). This is despite the fact that 6-fold more samples were loaded for *SMN2^SupG1C^*-transfected cells compared with the wild-type *SMN2^Sup^*-transfected cells (Figure 7B). We also determined the splicing pattern of the *SMN2^SupG1C^* super minigene in the presence of CHX that suppresses NMD by inhibiting translation. Enhanced skipping of *CCNT1* exon 7 (Figure 7G) as well as enhanced inclusion of exon 6B of endogenous *SMN1*/*SMN2* and *SMN2^Sup^* confirmed that the CHX treatment was effective (Figure 7B; Supplementary Figure S16). We observed a general increase in the signal intensity of MESDA of *SMN2^SupG1C^* in the presence of CHX, which validated that the splicing products generated from the *SMN2^SupG1C^* super minigene are indeed substrates of NMD, but the only appreciable change in splicing pattern was an increase in inclusion of exon 6B (Figure 7B, compare lanes 11 and 12).

To better characterize the transcripts coming from *SMN2^SupG1C^*, we performed qPCR using a variety of primer pairs designed to amplify the major isoforms based on the results of MESDA. Amplification using primers annealing to the FLAG tag and the junction of exons 1 and 2A resulted in robust amplification only in wild-type *SMN2^Sup^*. Expression was increased by ∼80% in the presence of CHX (Figure 7C). There was no amplification at all in the *SMN2 ^SupG1C^*-transfected samples, indicating that this mutation completely prevents splicing from the canonical 5′ss (Figure 7C). Next, we performed qPCR using primers annealing to the FLAG tag and the 5′ portion of intron 1. These primers should amplify several types of super minigene-derived transcripts: (i) nascent transcripts that have not completed transcription and splicing; (ii) intron 1-retained transcripts (both full length and stalled/prematurely terminated); and (iii) transcripts generated by using the cryptic 5′ss in intron 1. According to our results, intron 1-containing transcripts were expressed only at low levels in cells transfected with *SMN2^Sup^* (Figure 7D). In cells transfected with *SMN2^SupG1C^*, levels of these transcripts increased 8-fold (Figure 7D). Interestingly, although in *SMN2^Sup^*-transfected cells intron 1-containing transcripts were largely unaffected by CHX treatment, the treatment resulted in a >2-fold increase in expression of these transcripts in cells transfected with *SMN2^SupG1C^* (Figure 7D). We captured the activation of the cryptic 5′ss in intron 1 using a forward primer annealing to the pIn1–E2A junction and a reverse primer annealing within exon 2B (Figure 7E). In *SMN2^Sup^*-transfected cells, we observed low levels of the cryptic 5′ss usage; the levels increased ∼3-fold by CHX treatment. The In1G1C mutation resulted in a >4-fold increase in the cryptic 5′ss usage, with which was further increased by >3-fold in the presence of CHX (Figure 7E).

To achieve a rough estimate of the relative expression of *SMN2^Sup^* isoforms, we compared each isoform's expression level with that of *HPRT*. Although this calculation does not take into account the differences in amplification efficiency between different primer pairs, it is still useful for general relative expression estimates. The vast majority of transcript produced from *SMN2^Sup^* utilized the canonical 5′ss of exon 1, with very low levels of intron 1 retention or cryptic 5′ss usage (Figure 7F). In *SMN2^supG1C^*-derived transcripts, the cryptic 5′ss usage appeared to be a minor event compared with intron 1 retention (Figure 7F). This is surprising, since MESDA results showed that all amplifiable full-length transcript utilizes the cryptic splice site (Figure 7B). This discrepancy might indicate that the intron 1-containing transcripts that do not use the cryptic 5′ss are prematurely terminated or that pol II is stalled near the start of initiation.

We monitored the expression of SMN by western blot using antibodies against SMN. We observed a reduction in SMN expression from *SMN2^Sup^* in CHX-treated samples (Figure 7H). At the same time, CHX had no appreciable effect on the levels of SMN produced from the endogenous *SMN1/SMN2* genes. Due to the presence of a PTC within intron 1, transcripts generated from the *SMN2^SupG1C^* super minigene were not translated into SMN.

### Effect of SRSF3 and DHX9 on splicing of *SMN2* exons

**Figure 8.**
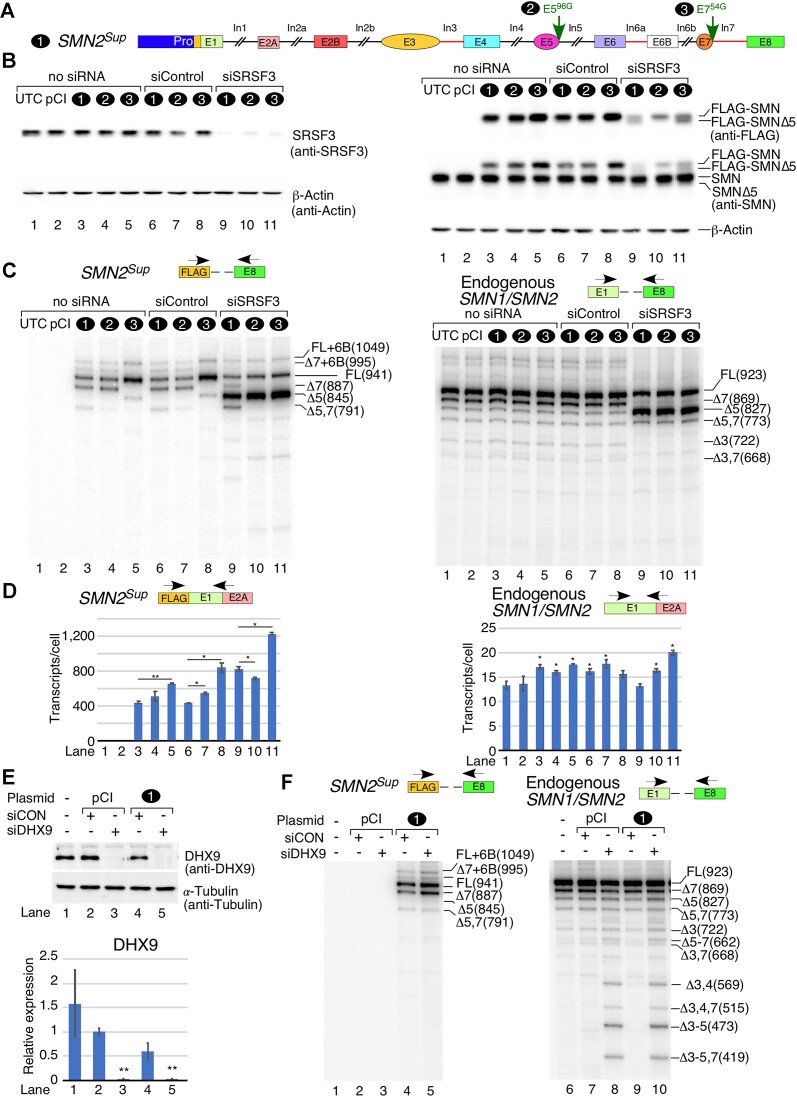
Effects of RNA processing factors on super minigene splicing. (**A**) Diagrammatic representation of the *SMN2* super minigene and mutants used in this figure. The locations of E5^96G^ and E7^54G^ mutations are indicated with green arrows. Each of the three constructs is assigned a number, which is then used in the figure as a construct identifier. (**B**) Representative western blot showing the expression levels of SRSF3 and SMN proteins in HeLa cells transfected with constructs shown in (A) and treated with siRNAs for SRSF3 knockdown. Transfected plasmids and siRNA treatments are indicated at the top of the image. (**C**) Splicing pattern of *SMN2^Sup^* (left panel) and endogenous *SMN1/SMN2* (right panel) in HeLa cells under conditions of SRSF3 knockdown, as determined by MESDA. Labeling is the same as in Figure 1C. (**D**) Estimated copies of *SMN2^Sup^* (left panel) and endogenous *SMN1/SMN2* (right panel) in HeLa cells under conditions of SRSF3 knockdown, as determined by qPCR. Samples are the same as in (C). Locations of primers used for qPCR are shown. (**E**) Upper panel: representative western blot showing expression of DHX9 after 96 h of knockdown using siRNA. Transfected plasmids and siRNA treatments are indicated at the top of the gel. Protein band identities and antibodies used for membrane probing are shown on the left side of the gel. Lower panel: densitometric quantification of protein expression from western blot. Abbreviations: siCON, non-targeting control siRNA; siDHX9, siRNA against DHX9. (**F**) Splicing pattern of *SMN2^Sup^* (left panel) and endogenous *SMN1/SMN2* (right panel) in HeLa cells transfected with *SMN2^Sup^* and siRNA against DHX9. Types of transfections are indicated at the top of the gel, and splice isoforms on the right side of the gel. The locations of primers used for MESDA are shown. For all quantifications, *n* = 3. Error bars indicate the SEM. *P < 0.05, **P < 0.01.

SRSF3 promotes inclusion of exon 5 from *SMN1*/*SMN2* (48). However, *cis-*elements associated with the regulation of exon 5 splicing have not yet been examined. To fill this gap, we started with testing whether depletion of SRSF3 would have a similar effect on splicing of transcripts generated from the *SMN2* super minigene and endogenous *SMN1/SMN2* genes. We used two additional super minigene mutants, *SMN2^E5-96G^* and *SMN2^E7-54G^*, in which the 5′ss of exons 5 and 7, respectively, were strengthened by A-to-G substitution at the last exonic position (Figure 8A). Of note, A-to-G substitution at the last position of exon 7 fully restores inclusion of *SMN2* exon 7 (49). Consistently, transcripts generated from *SMN2^E7-54G^* showed complete loss of splice variants lacking exon 7 (Figure 8C). Transcripts produced by *SMN2^E5-96G^* showed reduction in isoforms with co-skipped exons 5 and 7 (Figure 8C). We observed a moderate increase in levels of transcripts generated from *SMN2^E5-96G^* and *SMN2^E7-54G^*, suggesting that inclusion of exon 5 or 7 has a stabilizing effect on *SMN2* mRNA (Figure 8D). In agreement with the previous finding (48), depletion of SRSF3 caused massive skipping of exon 5 in both super minigene-derived transcripts and endogenous *SMN1*/*SMN2* (Figure 8C). Cloning and sequencing of bands from endogenous *SMN1/SMN2* confirmed the effect of SRSF3 depletion on both *SMN1* and *SMN2* (Supplementary Figure S17A). Transcripts generated from *SMN2^E5-96G^* did not show appreciable reduction in exon 5 skipping upon SRSF3 depletion, although, surprisingly, exon 7 skipping was reduced, as was the co-skipping of exons 5 and 7, suggesting a cross-regulation of splicing of exons 5 and 7 by SRSF3. As expected, transcripts generated from *SMN2^E7-54G^* showed complete inclusion of exon 7 upon SRSF3 knockdown, but no change in exon 5 skipping was observed (Figure 8C).

We examined the protein expression from *SMN2^Sup^*, *SMN2^E5-96G^* and *SMN2^E7-54G^* with and without depletion of SRSF3. When cells were treated with control siRNA, *SMN2^Sup^* and *SMN2^E5-96G^* produced equivalent levels of FLAG-SMN (Figure 8B, right panels). In contrast, *SMN2^E7-54G^* expressed noticeably more FLAG-SMN due to increased inclusion of exon 7. SRSF3 knockdown did not have a significant effect on expression of full-length SMN produced from endogenous *SMN1/SMN2;* however, a faint band that migrated slightly faster than full-length SMN appeared, which we attribute to SMNΔ5 produced from exon 5-skipped transcripts (Figure 8B). Notice that the intensity of the SMNΔ5 band relative to SMN is far less than expected based on the ratio of exon 5-skipped to full-length *SMN1/SMN2* transcript, suggesting that the SMNΔ5 protein is unstable (Figure 8B, C). This is consistent with our previous study, in which induction of massive exon 5 skipping was required to detect SMNΔ5 (35). Unlike the insignificant effect of SRSF3 depletion on expression of SMN from endogenous *SMN1/SMN2*, SRSF3 depletion exhibited a drastically reduced level of FLAG-SMN, along with the production of the faster migrating SMNΔ5. We attribute this discrepancy to two factors: (i) ∼90% skipping of exon 5 in transcripts generated from *SMN2^Sup^* upon SRSF3 depletion compared with ∼60% skipping of exon 5 in transcripts generated from endogenous *SMN1/SMN2;* and (ii) the presence of the residual SMN that was made before SRSF3 depletion. Although strengthening of the 5′ss of exon 5 in *SMN2^E5-96G^* did not prevent exon 5 skipping under the conditions of SRSF3 knockdown, we noticed increased FLAG-SMN levels compared with *SMN2^Sup^*, along with the loss of FLAG-SMNΔ5 (Figure 8B, C). *SMN2^E7-54G^* produced increased levels of both FLAG-SMN and FLAG-SMNΔ5, consistent with the decreased skipping of exon 7 and decreased co-skipping of exons 5 and 7 (Figure 8B). We also examined the effect of SRSF3 overexpression on *SMN2^Sup757^*. Although the effect of overexpression was much more subtle, we observed the inverse of the effect of knockdown, with slightly increased exon 7 skipping and slightly decreased exon 5 skipping in *SMN2^Sup^* (Supplementary Figure S17D-F).

The DHX9 helicase preferentially unwinds the secondary structures associated with Alu elements, although it can also regulate splicing through Alu-independent structures. Considering that *SMN2^Sup^* lacks Alu elements aside from the AluY element-containing exon 6B, it provides a unique system to evaluate the role of DHX9 in regulation of *SMN* splicing through Alu-independent secondary structures. Hence, we compared the splicing pattern of transcripts produced from *SMN2^Sup^* and endogenous *SMN* after depleting HeLa cells of DHX9 using siRNA. We confirmed the efficient depletion of DHX9 (Figure 8E). Supporting the role of Alu-associated structures in splicing of exons 3, 4 and 5, DHX9 depletion produced no effect on splicing of these exons in the context of the super minigenes (Figure 8F; Supplementary Figure S18). We also tested the biogenesis of circRNA C3-4, which is stimulated by DHX9 depletion (19). We did not observe any difference in C3-4 levels between pCI-transfected and *SMN2^Sup^-*transfected cells, confirming that Alu elements are required for C3-4 generation (Supplementary Figure S18B). However, we observed a mild DHX9 depletion-associated increase in skipping of exon 7 in transcripts generated from *SMN2^Sup^* (Figure 8F). These results underscored the role of DHX9 in regulation of exon 7 splicing through Alu-independent secondary structures within exon 7 and/or flanking intronic sequences. In order to rule out the possibility that endogenous *SMN1/SMN2* may be more susceptible to DHX9 depletion-associated skipping of exons 3 and 4 due to the presence of *SMN1*, we sequenced splice variants of endogenous *SMN1/SMN2* (data not shown). Our analysis revealed that both *SMN1* and *SMN2* pre-mRNAs are susceptible to co-skipping of these exons, confirming that the lack of intronic Alu elements is likely to be responsible for the loss of DHX9-triggered skipping of exons 3 and 4 in *SMN2^Sup^*. We also determined the effect of DHX9 depletion on levels of linear *SMN* transcripts. We detected a significant increase in the levels of linear transcripts generated from *SMN2* super minigenes (Supplementary Figure S18C). However, we did not detect a similar change in endogenous *SMN1/SMN2*.

### Super minigene as a reporter for splicing modulation of *SMN2* and mutant *SMN1*

We evaluated whether the *SMN2* super minigene could respond to a known splicing-modulating compound. For this, we co-transfected HeLa cells with the *SMN2* super minigene and 50 nM of an ASO identical to nusinersen, which is designed to stimulate *SMN2* exon 7 inclusion by targeting the negative intronic element ISS-N1 located immediately downstream of exon 7 (Supplementary Figure S19A). As a negative control, we used scrambled ASO with the same nucleotide composition but different sequence order. The ISS-N1-targeting ASO (Anti-N1) triggered almost complete inclusion of exon 7 in transcripts generated from both the super minigene and endogenous *SMN1/SMN2* (Supplementary Figure S19B, C). Consistently, FLAG-SMN protein expression was significantly increased (Supplementary Figure S19D, E). These results confirmed the suitability of *SMN2^Sup^* as a translation-competent reporter for screening splicing-modulating therapeutics of SMA.

**Figure 9.**
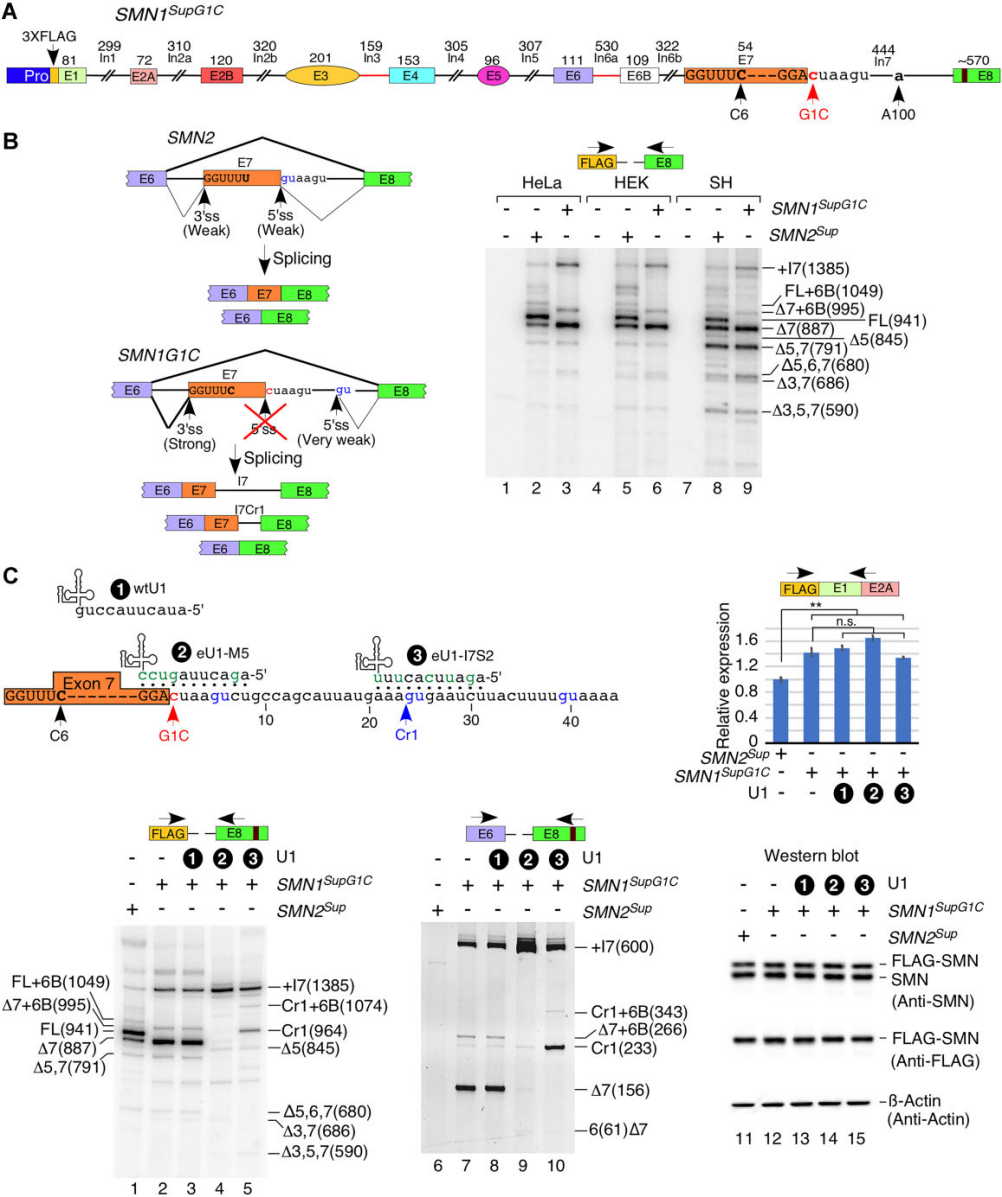
Modeling an atypical SMA case using an *SMN* super minigene. (**A**) Diagrammatic representation of the *SMN1^SupG1C^* super minigene. Exon 7 and intron 7 regions are expanded with sequence context to highlight key differences between *SMN1^SupG1C^* and wild-type *SMN2^Sup^*. C6 and A100 nucleotides that match the *SMN1* sequence are indicated with black arrows and letters in bold; the G1C mutation at the 5′ss of exon 7 is indicated with a red arrow and a letter in red. Exon 8 in *SMN1^SupG1C^* carries the same mutations as in the *SMN^2BM4^* super minigene. Other coloring and labeling are the same as in Figure 1B. (**B**) Comparison of splicing patterns of *SMN2^Sup^* and *SMN1^SupG1C^* as determined by MESDA. Left panel: overview of exon 7 splicing in *SMN2^Sup^* and *SMN1^SupG1C^*. Exons are depicted as colored boxes, and introns as lines. Key sequences of exon 7 are given, with the difference in a nucleotide at the sixth position highlighted by a letter in bold. The G1C mutation that inactivates the 5′ss of exon 7 is shown in red. The invariant GU dinucleotide of the 5′ss is indicated in blue. Splicing events are depicted with angled lines connecting the 5′ and 3′ss. Predicted splice products are shown below. Right panel: splicing patterns of *SMN2^Sup^* and *SMN1^SupG1C^* in three different cell lines, as determined by MESDA. Cell line names and the identity of the transfected plasmids are indicated at the top of the gel. Other labeling is the same as in Figure 1C. Abbreviations: HEK, HEK-293 human embryonic kidney cells; SH, SH-SY5Y neuroblastoma cells. (**C**) Splicing modulation of *SMN1^SupG1C^* using engineered U1 snRNAs. Top left panel: overview of eU1s used including their annealing to the intron 7 sequences. Mutations within eU1s are indicated in green. G1C mutation at the 5′ ss of exon 7 is shown in red. GU dinucleotides as part of the cryptic 5′ss are indicated in blue. Other labeling and coloring are the same as in Figure 6C. Top right panel: relative expression of *SMN2^Sup^* and *SMN1^SupG1C^* co-transfected with pCI-Neo or eU1s in HeLa cells as determined by qPCR. Lower left panel: splicing of *SMN2^Sup^* and *SMN1^SupG1C^* in HeLa cells in the presence of pCI-Neo or eU1s, as determined by MESDA. Labeling is the same as in Figure 1C. Lower middle panel: splicing of exon 7 in transcripts generated from *SMN1^SupG1C^*. The location of primers used for PCR is shown. Notice that one of the primers anneals to the mutated region of *SMN1^SupG1C^* exon 8 indicated by a dark red stripe. Lower right panel: representative western blot showing FLAG-tagged SMN protein expression produced from *SMN2^Sup^* and *SMN1^SupG1C^* in HeLa cells. The identities of transfected constructs are at the top of the image.

We examined whether our super minigene can recapitulate the splicing pattern of the endogenous *SMN1* carrying a pathogenic mutation associated with SMA. This mutation is located at the first position of intron 7 (G1C substitution); it causes skipping of *SMN1* exon 7, leading to severe SMA phenotype and patient death at ∼4 months of age (50). Importantly, tissue samples or cell lines bearing this and similar pathogenic mutations linked to SMA are not easily available. We constructed a super minigene, *SMN1Sup^G1C^*, by replacing *SMN2* sequences from exons 6–8 with *SMN1* sequences and inserting a G1C substitution at the first position of intron 7 (Figure 9A). Of note, sequence differences between *SMN1* and *SMN2* upstream of exon 6 are not associated with any known splicing changes (35). We examined the splicing pattern of *SMN1Sup^G1C^* in three cell lines, i.e. HeLa, HEK-293 and SH-SY5Y (Figure 9B; Supplementary Figure S20). As expected, we observed complete skipping of exon 7 as well as intron 7 retention in the super minigene-derived transcripts in all cell types tested. However, the pattern of co-skipping of exons was different in different cell lines. For example, we observed more co-skipping of exons 5 and 7 in SH-SY5Y than in other cell types. All cell types examined showed a small but detectable level of exon 6B-included transcripts. In general, the splicing pattern of *SMN1^SupG1C^* mirrored that of *SMN2^Sup^*, with the exception of exon 7-included transcripts that were absent in the case of *SMN1^SupG1C^* (Figure 9B).

Employing short minigenes, we have previously shown that eU1s targeting sequences downstream of the mutated 5′ss of *SMN1* exon 7 could activate a cryptic 5′ss (Cr1) at the 23rd intronic position (45). Here, we examined if the relative abundance of transcripts produced by *SMN1^SupG1C^* can also be altered by eU1s. As a control, we used wild-type U1 (wtU1) that had no appreciable effect on the splicing pattern of *SMN1^SupG1C^*. As expected, eU1s targeting the mutated 5′ss and Cr1 promoted intron 7 retention and Cr1 activation, respectively (Figure 9C). However, eU1 targeting Cr1 also induced small but detectable levels of inclusion of exon 6B. Considering that our super minigene is translation competent, we examined if FLAG-tagged SMN protein is made from *SMN1^SupG1C^*. Interestingly, we discovered that SMN expression from *SMN1^SupG1C^* was comparable with that generated from *SMN2^Sup^* (Figure 9C, right panel). This is not entirely surprising given the fact that intron 7-containing transcripts, exon 6B-containing transcripts and transcripts generated from Cr1 activation are translated (17,45,51). Along the expected lines, we did not capture any appreciable difference between untreated and eU1-treated samples. Further complicating the interpretations, transcripts containing intron 7 or those generated from Cr1 activation are likely to be translated with different efficiencies.

## Discussion

We report here the generation of a transcription-, splicing- and translation-competent *SMN2* super minigene, *SMN2^Sup^*, comprised of all nine canonical exonic sequences and the rare exon 6B in the context of their flanking intronic sequences. *SMN2^Sup^* encompasses the *SMN2* promoter and the entire 3′UTR sequence of *SMN2*, making it uniquely capable as a reporter system to study the role of transcription and post-transcriptional events in the production of SMN. Pre-mRNA transcribed from *SMN2^Sup^* underwent efficient splicing, recapitulating the splicing pattern of transcripts generated from endogenous *SMN1*/*SMN2* genes in all cell lines examined, namely HeLa, HEK-293, SH-SY5Y and GM03813 (Figure 1; Supplementary Figures S1, S3 and S9). Of note, *SMN2^Sup^* lacks sequences that generate two antisense transcripts, one from intron 1 and the other from downstream sequences spanning from intron 5 to the intergenic region of *SMN1/SMN2* (52,53). The absence of these sequences appears to have no impact on splicing of *SMN2* exons. Replacement of the *SMN2* promoter with the mouse *Smn* promoter did not change the transcription efficiency or the splicing pattern of *SMN2^Sup^*. However, surprisingly, the *SMN2* super minigene under the control of a strong, constitutive CMV promoter (*SMN2^Sup-CMV^*) produced transcripts with enhanced skipping of exons 3, 5 and 7 when cells were transfected with high amounts of the plasmid. *SMN2^Sup-CMV^* also triggered skipping of exon 3 in endogenous *SMN1/SMN2*, although the effect was less prominent, probably due to high background of untransfected cells. Our results suggest that uncontrolled expression of SMN protein results in skipping of the alternatively spliced exons 3, 5 and 7, possibly to serve as a feedback loop. We show that mRNA generated from the super minigene is better translated than mRNA generated from the cDNA clone, consistent with the earlier observation that exon junction complex (EJC) deposited during splicing promotes translation (54).

We examined if the regulatory sequences within the promoter region contributed towards *SMN2* transcription and/or splicing modulation of specific exons. Validating the findings of previous reports, a large deletion reducing the length of the *SMN2^Sup^* promoter from 3445 to 757 nt had a strong stimulatory effect on transcription of *SMN2^Sup^* from the super minigene (Figure 2B) (27,28). Small overlapping deletions within the 757 nt long promoter revealed a high density of positive regulatory elements spanning the 100 nt region immediately upstream of the ATG start codon. Similar results were obtained with the 370 nt long promoter (Figure 2D; Supplementary Figure S8). Our results also revealed the overlapping nature of the positive and negative *cis-*elements located between the 57th and 72nd position upstream of ATG. Potential factors binding to this core region may include, but are not limited to, ETS and SP/KLF family of proteins (Supplementary Figure S2). For instance, Δ72–63 deletion caused increased transcription, supporting the loss of a negative *cis-*element, whereas Δ68–57 deletion resulted in decreased transcription, supporting the loss of a positive *cis-*element. However, these results should be interpreted with caution because deletions and substitutions have the potential to create artificial motifs. Notably, none of the deletions in the promoter region affected the splicing pattern of transcripts generated from the super minigene (Supplementary Figure S6). These results ruled out the role of promoter-associated factors in splicing regulation of *SMN2* in HeLa cells.

The results of 5′RACE performed on transcripts generated from the *SMN2* super minigene as well as from endogenous *SMN1*/*SMN2* revealed TSS^14–17^ located between 14 and 17 nt upstream of the ATG as the primary TSS (Figure 3). A secondary TSS, TSS^48–51^, was located at 48 and 51 nt upstream of ATG. We confirmed that identical TSSs are used in all cell lines examined. Surprisingly, insertion of FLAG sequence in front of the ATG start codon shifted the TSS upward, suggesting a fine regulation of TSS by *cis-*elements in the promoter-proximal region. We investigated the promoter-proximal region using small overlapping deletions with the goal to determine how the absence of small motifs affects transcript levels, TSS selection and the translation of the processed transcripts. While many deletions upstream of TSS^14–17^ drastically reduced transcript levels, TSS^14–17^ remained as the primary site. Deletion of TSS^14–17^ or neighboring sequences activated alternative TSSs, although, to our surprise, the overall transcript levels were not affected. In some instances, deletion of TSS^14–17^ or neighboring sequences unexpectedly shifted the TSS to intron 1 in a subset of transcripts. However, transcripts with a TSS in intron 1 are likely to be poorly represented due to their potential for degradation by NMD. According to our results of RACE, the *SMN* genes have an exceptionally short 5′UTR, shorter than the 20 bases generally recognized as the minimum size for cap-dependent ribosome scanning and translation (55). Many mammalian genes with extremely short 5′UTRs such as *SMN1/SMN2* harbor a specialized version of the Kozak sequence known as a translation initiator of short 5′UTR (TISU) (56,57). The consensus sequence of TISU SAASATGGCGGC, where S represents C or G and ATG represents the start codon, is not an exact match for *SMN* (TGCTATGGCGAT); however the core six nucleotides (ATGGCG) which are invariant in all TISU elements, are present. Consistently, deletion of the GCG or removing the first ATG start codon drastically reduces translation from the *SMN2* super minigene (Figure 3F). TISU elements also play a role in transcription initiation (57). Supporting this argument, deletion of the GCG motif reduced the fidelity of transcription initiation and decreased levels of transcripts produced from the *SMN2* super minigene. The size and nature of the 5′UTR displayed a profound effect on translation of the processed transcripts. For instance, transcripts with deletion of the 5′-most bases of the 5′UTR had significantly lower protein expression.

We investigated the potential causes of enhanced skipping of endogenous exon 3 under the conditions of CMV-driven overexpression of the *SMN2* super minigene. The results of our experiments showed a rather unexpected correlation between increased levels of SMN protein and skipping of exon 3 (Figures 4 and 5). Surprisingly, overexpression of SMNΔ7 produced an even greater effect on exon 3 skipping compared with SMN. SMN is an RNA-binding protein with preference for structured RNA (58). Considering that exon 3 of *SMN1*/*SMN2* folds into an elaborate secondary structure (19), it may serve as a direct target for SMN binding. The nucleic acid-binding domain coded by exons 2A and 2B of *SMN1*/*SMN2* are retained in most of the alternatively spliced transcripts (25). Hence, overexpression of any of these splice variants is likely to trigger skipping of exon 3. EJCs deposited at neighboring exons are known to modulate removal of introns (59). Hence, it is possible that the elevated levels of SMN promote exon 3 skipping by preventing the recruitment of EJCs at the junction of exons 4 and 5. It is also likely that the overexpression of SMN adversely affects R-loop resolution, an SMN-dependent process linked to transcription-coupled splicing regulation (60,61). Supporting our findings, a recent report demonstrated perturbations of RNA metabolism upon overexpression of SMN in a mouse model of SMA (62). In contrast, another report showed a correlation between reduced SMN levels and skipping of *SMN2* exon 7 (63). However, this study did not evaluate the splicing of upstream exons. It is likely that both extreme low and high levels of SMN have adverse effects on splicing regulation of *SMN1* and *SMN2* exons. A recent report showed a multi-fold increase in expression of *SMN1*/*SMN2* transcripts in laryngeal squamous cell carcinoma (LSCC) (64). Future studies will determine if splicing of *SMN1*/*SMN2* transcripts is dysregulated in LSCC or other pathological conditions with elevated SMN levels.

The transcription elongation rate and pausing of pol II are affected by a combination of factors including chromatin structure, template DNA sequence and sequence/structure of the transcribed RNA (29). In regard to chromatin structure and its formation on plasmid DNA, it has been shown that transiently transfected plasmid DNA can be converted to the supercoiled form, indicative of nucleosome formation (65). The size of pre-mRNA generated from *SMN2^Sup^* is ∼10-fold smaller than that of the pre-mRNA transcribed from endogenous *SMN1/SMN2*. We compared how the transcription elongation inhibitor CPT influenced splicing of pre-mRNAs produced from *SMN2^Sup^* and endogenous *SMN1/SMN2* (Figure 6). We observed a similar effect of CPT on splicing of these pre-mRNAs despite a large difference in the size of these transcripts. These results independently confirmed the recent observation that *SMN2* exon 7 is a class II exon and support that the alternatively spliced exon 3 of *SMN1*/*SMN2* also belongs to the class II category (32). Our findings underscored that the effect of CPT is mostly due to pre-mRNA sequences surrounding the splice sites. Splicing of exons 3 and 7 was the most affected by CPT in both endogenous and super minigene-derived *SMN* pre-mRNA. Our results supported that CPT might interfere with the definition of the 5′ss of exon 7 since expression of eU1 targeting the 5′ss of exon 7 fully suppressed the inhibitory effect of CPT (Figure 6). However, the mechanism behind CPT-induced skipping of exon 3 appeared to be more complex as eU1 targeting the 5′ss of exon 3 failed to abrogate the inhibitory effect of CPT on splicing of this exon. It is also possible that this eU1 was less efficient. We observed CPT-induced enhanced co-skipping of exons 5 and 7 as well as exons 3 and 5, while the transcript with exon 5 skipped alone was maintained at steady levels. These results suggest that the CPT-induced skipping of exon 5 only happens in pre-mRNAs in which exon 3 or exon 7 had already been skipped. Supporting this argument, eU1 that targeted the 5′ss of exon 7 decreased the levels of co-skipping of exons 5 and 7 without affecting the levels of exon 5-skipped transcripts.

Similar to many eukaryotic genes, the first intron of *SMN1/SMN2* is the longest intron among all eight annotated introns (66). Owing to their proximity to the TSS, specific chromatin organization, presence of enhancer motifs and the recursive splicing phenomenon, first introns play a unique regulatory role (67,68). *SMN* intron 1 is ∼13.6 kb long and encompasses three cryptic exons and many repeat elements, including 22 Alu-like sequences (18,19). Shortening of intron 1 in our super minigene that resulted in the loss of all repeat elements did not produce an adverse effect on intron 1 removal or on overall splicing (Figure 1B). A G1C mutation at the first position of intron 1 activated a cryptic 5′ss, resulting in retention of a 117 nt long intronic sequence that we dubbed pIn1 (Figure 7). pIn1 retention had no adverse effect on removal of downstream introns, although pIn1-containing transcripts had increased co-skipping of exons 5 and 7 and showed reduced stability due to NMD. Transcripts with retained pIn1 could potentially maintain a large open reading frame (ORF) set up by non-canonical translation initiation codons CUG and GUG present within exon 2. Of note, CUG and GUG usage as non-canonical translation initiation codons has been shown before, albeit with low efficiency (69). However, using SMN antibody, we did not detect this N-terminal truncated SMN isoform translated from pIn1-containing transcripts. This result underscored that the above-mentioned potential start codons in exon 2 are not used for translation.

We employed the *SMN2* super minigene as a reporter to capture how mutations that strengthen the 5′ss of exon 5 or exon 7 impact splicing, transcript levels and translation. We also examined if SRSF3 (SRp20) modulates transcription, pre-mRNA splicing and translation of transcripts derived from the *SMN2* super minigene encompassing mutations with strengthened 5′ss of exons 5 or 7. The ubiquitously expressed SRSF3 is the smallest SR protein comprised of only 164 amino acids and implicated in many aspects of RNA metabolism, including transcription, splicing, polyadenylation and RNA transport (70). Validating the previously reported finding (48), depletion of SRSF3 caused massive skipping of exon 5 in transcripts generated from the *SMN2* super minigene and endogenous *SMN1*/*SMN2* (Figure 8). Our results also confirmed that the internal deletions within intronic sequences in the context of the super minigene had no bearing on splicing regulation of exon 5. A mutation that strengthened the 5′ss (E5^96G^) of exon 5 failed to prevent skipping of exon 5, suggesting that *cis-*elements outside of the 5′ss of exon 5 could be critical for regulation of its splicing. The strengthened 5′ss of exon 5 also promoted inclusion of exon 7, supporting a co-regulation of exon 5 and 7 splicing by SRSF3. We observed up-regulation of transcripts derived from the super minigene with the strengthened 5′ss of exons 5 or 7, suggesting that inclusion of these exons has a stabilizing effect on mRNA. Our super minigene system efficiently captured the effect of SRSF3 at the protein level and showed that the exon 5-skipped transcripts produce barely detectable SMNΔ5 protein, probably due to its reduced stability and/or poor translation efficiency.

The multifunctional protein DHX9, a DNA/RNA helicase, is associated with several aspects of gene regulation including DNA repair, transcription and pre-mRNA splicing (71,72). Recent reports support the role of DHX9 in suppression of circRNA generation, including generation of circRNAs from *SMN*, through preferential unwinding of secondary structures associated with inverted Alu repeats (19,20,24). We employed the *SMN2* super minigene to determine if DHX9 affects splicing of its exons in the absence of secondary structures formed by inverted Alu repeats (Figure 8). Depletion of DHX9 enhanced skipping of exon 7 in super minigene-derived transcripts, supporting the role of Alu-independent structures in DHX9-associated splicing regulation of exon 7. Previous studies on *SMN2* exon 7 splicing regulation revealed sequestration of the 5′ss of exon 7 by local secondary structures and structures formed by long-distance interactions (73). Predicted structures also support the potential looping out of exon 7 through inter-intronic interactions (74). It is possible that DHX9 promotes exon 7 inclusion through abrogation of one or more of these structures. Our finding that depletion of DHX9 increased transcription of *SMN2^Sup^* was somewhat surprising given the role of DHX9 as an enhancer of transcription of several genes associated with cancer (72). DHX9 is known to interact with promoter sequences and enhances transcription through resolution of the R-loop in the promoter-proximal region (75–77). There is also opposing evidence to support that DHX9 promotes formation of R-loops in cells with impaired RNA splicing (78). It is likely that the latter mechanism is applicable due to the negative effect of DHX9 on transcription of the *SMN2* super minigene. Alternatively, enhanced transcription under the condition of DHX9 depletion is offset by the negative effects of unresolved Alu–Alu base pairing in the context of endogenous *SMN1/SMN2*.

One of the most important applications of the super minigene could be modeling of pathogenic mutations. Once such mutations are introduced in the super minigene, their effects could be monitored at multiple levels including transcription, pre-mRNA splicing, translation and cellular localization in different cell types. The super minigene could be further utilized to screen for therapeutic compounds that enhance SMN expression in a cell-specific manner. We provide two proofs of principles validating the utility of the super minigene. First, the *SMN2* super minigene treated with the therapeutic ASO showed robust inclusion of exon 7 and consequently produced elevated levels of SMN (Supplementary Figure S19). Second, we captured cell-specific splicing of transcripts generated from a super minigene carrying a pathogenic mutation linked to SMA (G-to-C mutation at the first position of *SMN1* intron 7) (Figure 9). Our findings are significant given the fact that no information on tissue-specific splicing of this pathogenic mutation is currently available. We also show that the level of SMN expressed from the super minigene carrying this pathogenic mutation is comparable with that generated from the *SMN2* super minigene, consistent with the severe SMA cases resulting from deletions or mutations of *SMN1* genes. In summary, the super minigene we report here represents a unique, powerful and easily amenable tool for discovering regulatory elements associated with several aspects of RNA metabolism as well as for therapeutic and diagnostic applications.

## Supplementary Material

gkad1259_Supplemental_Files

## Data Availability

All major splice variants and sequences of key super minigenes reported in this study can be accessed through the NCBI; accession numbers are listed in Supplementary Tables S2 and S3.
