# Supplementary material for: A super minigene with a short promoter and truncated introns recapitulates essential features of transcription and splicing regulation of the SMN1 and SMN2 genes
Source: Nucleic Acids Res. 2024 Jan 12;52(7):3547–71. doi: 10.1093/nar/gkad1259 (PMC11040157; doi:10.1093/nar/gkad1259)

SUPPLEMENTARY DATA

Supplementary Figure legends

Supplementary Figure S1. Construction of the *SMN2* super minigene. (Relates to Figure 1) Diagrammatic representation of strategy for the construction of *SMN2*^{Sup3445} in pCI-Neo backbone. Exons are depicted as colored shapes, and introns are shown as lines. The sizes of exons and intron fragments are indicated by numbers. Restriction enzyme sites are shown as gray boxes. Each PCR fragment is labeled in red. Arrows indicate stepwise PCR, with small green arrows representing smaller fragments being incorporated into the larger fragment represented by larger blue arrows.

Supplementary Figure S2. Structure of the human *SMN2*, mouse *Smn* and CMV promoters. (Relates to Figure 1) (**A**) Diagrammatic representation of human *SMN2*, mouse *SMN*, and CMV promoters. All predicted promoter cis-elements are indicated as colored shapes. Numbering is relative to the canonical transcription start site (TSS). (**B**) Sequence and locations of relevant predicted promoter cis-elements in the region surrounding the predicted transcription start sites (TSSs) of human *SMN2*. Cis-elements are indicated by colored boxes. TSSs are indicated with arrows. The ATG start codon is marked. All numbering is relative to the ATG.

Supplementary Figure S3. Characterization of endogenous *SMN1/SMN2* in cells transfected with *SMN2* super minigenes and quantification of splice isoforms identified in Figure 1 (Relates to Figure 1) (**A**) Diagrammatic representation of *SMN2*^{Sup} with three different promoters. Exons and promoters are shown as colored shapes, introns with deletions as broken black lines, and full introns as red lines. The size of each exon and intron are indicated. Each construct is assigned a number (indicated on the left), which is then used throughout the figure. (**B**) Upper panel: Splicing pattern of endogenous *SMN1/SMN2* transcripts in HeLa cells transfected with the indicated super minigenes, as determined by MESDA. HeLa cells grown in 6-well plates were transfected with either 0.1 µg (Lo) or 1.0 µg (Hi) of the indicated plasmid. Transfections are indicated at the top of the gel. Splice isoform identities are marked at the right side of the gel. Δ indicates exon skipping. Abbreviations: UTC, untransfected control; pCI, control transfected with empty pCI-Neo vector alone. Lower panel: Estimated copies per HeLa

cell of endogenous *SMN1/SMN2* transcripts. Absolute copy numbers were estimated using a plasmid standard curve and copies/cell estimated using the assumption that each HeLa cell contains approximately 30 pg of RNA. (**C**) Upper panel: Splicing pattern of endogenous *SMN1/SMN2* transcripts in transfected GM03813 cells, as determined by MESDA. 1.0 µg of each plasmid was used for transfection. Lower panel: qPCR measuring the estimated copies per GM03813 cell of endogenous *SMN2* transcripts. (**D**) Quantification of relative amount of splice isoforms detected by MESDA (see Figure 1B and (C)). Y axis represents the percentage of total *SMN* transcripts as measured by gel densitometry.

Supplementary Figure S4. Introduction of transcriptional pause site eliminates leaky expression. (Relates to Figure 2) (**A**) Diagrammatic representation of promoter/promoterless constructs used for the experiment. Promoter sequence is depicted as a black line, all sequences after ATG are in green. ATG is marked in red. TPS is indicated with a blue circle. 1X means one TPS; 4X, four TPSs. Abbreviations: TPS, transcriptional pause site. (**B**) Left panel: Semi-quantitative PCR examining the expression and splicing of $SMN2^{Ex1\text{-}4}$ in HeLa cells transfected with promoterless constructs with different copy numbers of the upstream TPS. The expected PCR product and locations of primers for PCR are shown. Transfected constructs are indicated at the top of the gel. Splice isoforms are indicated at the right of the gel. Right panel: Semi-quantitative PCR examining the expression and splicing of endogenous *SMN* in HeLa cells transfected with the indicated constructs. Abbreviations: UTC, untransfected control; Lipo only; plasmid DNA was omitted from transfection with Lipofectamine-2000; pCI, control transfected with empty pCI-Neo vector alone. (**C**) qPCR measuring relative expression of $SMN2^{Sup}$ transcript in HeLa cells transfected with the indicated constructs. Expression is set as relative to expression from the WT 757 promoter. Fold change between different pairs of constructs is given at the right.

Supplementary Figure S5. Controlling transcript level by transfection efficiency. (Relates to Figure 2) (**A**) Diagrammatic representation of seven super minigene constructs used to determine the minimum size of the *SMN* promoter required for $SMN2^{Sup}$ transcription. The size/name of each promoter construct is noted at the left side. Each promoter is given a number (at the left side), which is used to identify that construct throughout the figure. Important sequence elements

are shown as colored boxes. Lines indicate deleted regions within each construct. Numbering is relative to the ATG. Abbreviations: TPS, transcriptional pause site. (**B**) qPCR measuring estimated copies per HeLa cell of transfected *SMN2*^{Sup} plasmid. (**C**) Estimation of transfected plasmid DNA amount relative to genomic DNA, as measured using genomic endogenous *SMN* sequences as a normalizing control. (**D**) Estimated RNA copies per transfected plasmid molecule. This was achieved by dividing RNA copies in Figure 2B by DNA copies in (B).

Supplementary Figure S6. Characterization of *SMN2*^{Sup} splicing in the presence of promoter deletions. (Relates to Figure 2) (**A**) Diagrammatic representation of seven super minigene constructs used to determine the minimum promoter length required for *SMN2*^{Sup} transcription. The size/name of each promoter construct is noted at the left side. Each promoter is given a number (at the left side), which is used to identify that construct throughout the figure. Important sequence elements are shown as colored boxes. Lines indicate deleted regions within each construct. Numbering is relative to the ATG. Abbreviations: TPS, transcriptional pause site. (**B**) Left panel: Splicing patterns of *SMN2*^{Sup} expressed in HeLa cells transfected with the indicated super minigenes, as determined by MESDA. Labeling is the same as in Figure 1C. Right panel: Quantification of isoforms identified in MESDA. Y axis represents the percentage of total *SMN* transcripts as measured by gel densitometry. Transfections are indicated below the graphs. Each splice isoform is color coded. (**C**) Diagrammatic representation of deletions made within the 271-base proximal promoter region of *SMN2*^{Sup757}. Pink shaded region indicates areas where deletions have a significant negative impact on *SMN2*^{Sup} expression. Red shaded region indicates the region with the strongest effect on expression. Numbering is relative to the ATG, which is marked in red. (**D**) Upper panel: Splicing patterns of *SMN2*^{Sup} expressed in HeLa cells transfected with the indicated super minigenes, as determined by MESDA. Lower panel: Quantification of splice isoforms identified in MESDA. Labeling is the same as in (B).

Supplementary Figure S7. Identification of exon 2B mutations that provide a unique primer binding site. (Relates to Figure 2) (**A**) Sequences of WT *SMN2* exon 2B and fourteen exon 2B mutants with a partially randomized region. The region is boxed in yellow, with mutated bases shown in red. Randomization was designed to maintain the SMN coding sequence. Base positions are numbered relative to the start of exon 2B. (**B**) Splicing patterns of

SMN2^{Sup} expressed in HeLa cells transfected with the indicated super minigenes, as determined by MESDA. Abbreviations: WT, *SMN2*$^{Sup\text{-}371T}$; E8M, *SMN2*$^{Sup\text{-}CMV}$ with a mutated primer binding site in exon 8; FL- , *SMN2*$^{Sup\text{-}371T}$ with the 3XFLAG tag removed. (**C**) QPCR results verifying that mutants cannot be amplified by primers targeting WT *SMN2* exon 2B (**D**) Comparison of amplification between a 3′ primer targeting the constant region at the first 20 bases of exon 2B (upper panel) and a 3′ primer targeting the WT version of the mutated region from nucleotides 21 to 40 of exon 2B.

Supplementary Figure S8. Comparison of 40 base promoter deletions in *SMN2*^{Sup757T} and *SMN2*^{2BM4}. (Relates to Figure 2) (**A**) Diagrammatic representation of super minigenes used in this figure. Coloring and labeling are the same as in Figures 1B and 2A. (**B**) Expression of *SMN2*^{Sup757T} containing 40 base deletions in the promoter region. Left panel: Diagrammatic representation of 40 nt deletions made in *SMN2*^{Sup757T}. The size/name of each promoter construct is noted at the left side. Each promoter is given a number (at the left side), which is used to identify that construct throughout the figure. Important sequence elements are shown as colored boxes. Lines indicate deleted regions within each construct. Numbering is relative to the ATG. Right panel: Estimated *SMN2*^{Sup} copies per HeLa cell transfected with the indicated super minigenes. (**C**) Expression of *SMN2*^{2BM4} containing 40 nt deletions in the promoter region. Left panel: Diagrammatic representation of 40 nt deletions made in *SMN2*^{2BM4}. Coloring and labeling are the same as in (B). Right panel: Estimated *SMN2*^{2BM4} transcripts per HeLa cell transfected with the indicated super minigenes.

Supplementary Figure S9. Transcription initiation and splicing in diverse cell lines. (Relates to Figure 3) (**A**) Left panel: 5′RACE PCR of *SMN2*^{2BM4} in five cell lines of diverse lineages. Primer binding locations, cell lines, and whether cells were transfected with super minigene are indicated at the top of the gel. TSS previously identified by cloning and sequencing are labeled on the right side. Abbreviations: UNIV, universal primer binding site; HEK, HEK293 human embryonic kidney cells; SH, SH-SY5Y neuroblastoma cells; GM, GM03813 SMA patient fibroblasts; NSC, NSC34 mouse motor neuron-like neuroblastoma cells. Right panel: 5′RACE PCR of endogenous *SMN1/SMN2* in the four human cell lines used in the left panel. (**B**) Splicing pattern of *SMN2*^{Sup} (upper left panel) and endogenous *SMN1/SMN2* (upper right panel) in the cell

lines depicted in (A) as determined by MESDA. Labeling is the same as in Supplementary Figure S3. Lower panels: Quantification of splice isoforms identified by MESDA.

Supplementary Figure S10. Quantification of transcripts generated by 6 base deletions near the TSS. (Relates to Figure 3) Upper panel: Sequences of all deletion mutants in the regions surrounding the TSS. Numbering is relative to the ATG start codon. TSSs identified by sequencing of 5′RACE products are shown in green. Deletions are indicated by red dashes. Predicted start codon is shown in gold. Lower panels: Relative expression of *SMN2*^{2BM4} in HeLa cells transfected with the indicated super minigenes.

Supplementary Figure S11. Detailed view of Ex3-M1 and Ex3-M2 mutations and quantification of splice isoforms identified by MESDA. (Relates to Figure 4) (**A**) PRO-seq reads throughout the *SMN* gene body delineating potential transcriptional pause sites. *SMN* exons and introns are shown at the top. Positive strand reads from PRO-seq are shown as blue bars, negative strand reads as red. Scale bar representing 1 kb is at the bottom right. (**B**) Diagrammatic representation of overexpression constructs used in this figure. Mutated portions of exon 3 are indicated with red stripes. Other coloring and labeling are the same as in Figure 1B. Sequences of WT exon 3, M1 and M2 mutants are given below with mutated nucleotides shown in red. Sequence numbering is relative to the start of exon 3. (**C**) Quantification of splice isoforms identified by MESDA in Figure 4B (upper panels). Y axis represents the percentage of total *SMN* transcripts as measured by gel densitometry. Transfections are indicated below the graphs. Color coding for each splice isoform is explained below graphs. (**D**) Quantification of exon 3 skipping/inclusion identified in Figure 4B (lower panels). Color coding for full-length (FL) and exon 3-skipped isoform (Δ3) are indicated at the right side of each graph. Other labeling is the same as in (C). (**E**) Quantification of isoforms identified by MESDA in Figure 4D. Labeling is the same as in (C)**.**

Supplementary Figure S12. Estimating proportion of *SMN1* and *SMN2* in alternatively spliced products. (Relates to Figure 4) Upper panel: Splicing pattern of endogenous *SMN1/SMN2* in HeLa cells expressing *SMN2*^{Sup} driven by the *SMN2* promoter or the strong CMV promoter as determined by MESDA. Products were amplified using a 3′ primer located

close to the end exon 8. Plasmids used and transfection amounts are indicated at the top of the gel. For all visible bands besides FL and Δ7, bands were cut and cloned for Sanger sequencing. Presence of a DdeI site unique to *SMN2* was used to distinguish *SMN1* from *SMN2.* Number of clones corresponding to *SMN1* and *SMN2* are indicated in the colored table to the right. Lower panel: Splicing pattern of $SMN2^{Sup}$ in HeLa cells transfected with the indicated super minigenes. In both panels a DNA molecular weight marker was loaded in lane 1. The sizes of marker bands are given at the left of the gel. Splice isoforms are indicated at the right of the gel. Abbreviations: UTC, untransfected control; pCI, control transfected with empty pCI-Neo vector alone.

Supplementary Figure S13. Quantification of splice isoforms identified in Figure 5. (**A**) Quantification of isoforms identified by MESDA in Figure 5B. Y axis represents the percentage of total *SMN* transcripts as measured by gel densitometry. Transfections are indicated below the graphs. Color coding of splice isoforms is given at the bottom. (**B**) Quantification of isoforms identified by MESDA in Figure 5D. Labeling is the same as in (A).

Supplementary Figure S14. Treatment of HeLa cells with CPT to induce exon skipping. (Relates to Figure 6) (**A**) Splicing pattern of $SMN2^{Sup}$ (left panel) and endogenous *SMN1/SMN2* (right panel) in HeLa cells transfected with $SMN2^{Sup757}$ and treated with different concentration of CPT, as determined by MESDA. Treatments and transfections are indicated at the top of the gels. Splice isoforms are indicated at the right side. The locations of primers used for MESDA are shown. Abbreviations: CPT, camptothecin; DMSO, dimethyl sulfoxide; pCI, empty pCI-Neo vector. (**B**) Splicing pattern of endogenous *SMN1/SMN2* in HeLa cells treated with the indicated concentration of CPT. PCR was carried out using a 3′ primer located close to the end of exon 8. Except bands corresponding to full-length (FL) and Δ7 splice isoforms, all other visible bands were cut, DNA eluted, cloned and subjected to Sanger sequencing. Presence of a DdeI site unique to *SMN2* was used to distinguish *SMN1* from *SMN2.* Number of clones corresponding to *SMN1* and *SMN2* are indicated in the colored table to the right.

Supplementary Figure S15. Quantification of splice isoforms identified by MESDA in Figure 6. (**A**) Quantification of splice isoforms identified by MESDA in Figure 6A. In the left panel, only samples transfected by $SMN2^{Sup}$ are represented. Y axis represents the percentage of

total *SMN* transcripts as measured by gel densitometry. Transfections and treatments are indicated below the graphs. Color coding for splice isoforms are indicated at the bottom. (**B**) Quantification of splice isoforms identified by MESDA in Figure 6C, left panel. Labeling is the same as in (A).

Supplementary Figure S16. Quantification of splice isoforms identified by MESDA in Figure 7 and characterization of endogenous *SMN1/SMN2* splicing. (**A**) Quantification of splice isoforms generated from wild type *SMN2*^{Sup} in Figure 7B. Y axis represents the percentage of total *SMN* transcripts as measured by gel densitometry. Treatments are indicated at the bottom of the graph. Color coding for splice isoforms are indicated at the bottom. (**B**) Quantification of splice isoforms generated from *SMN2*^{SupG1C} in Figure 7B. Labeling is the same as in (A)**.** (**C**) Upper panel: Splicing pattern of endogenous *SMN1/SMN2* in HeLa cells transfected with the indicated super minigenes and/or treatment with DMSO and CHX as determined by MESDA. Lower panel: Quantification of splice isoforms generated from endogenous *SMN*1/*SMN2*. Labeling is the same as (A).

Supplementary Figure S17. SRSF3 impacts multiple exons of *SMN1/SMN*: Additional data. (Related to Figure 8) (**A**) Splicing pattern of endogenous *SMN1/SMN2* in HeLa cells after knockdown of SRSF3 as determined by MESDA. PCR was carried out using a 3′ primer located close to the end of exon 8. Except bands corresponding to full-length (FL) splice isoform, other bands were cut, DNA eluted, cloned and subjected to Sanger sequencing. Presence of a DdeI site unique to *SMN2* was used to distinguish *SMN1* from *SMN2.* Number of clones corresponding to *SMN1* and *SMN2* are presented in the colored table to the right. (**B**) Quantification of *SMN2*^{Sup} splice isoforms identified by MESDA portrayed in Figure 8C (left panel). Super minigene and siRNA transfections are indicated at the bottom of the graph. Color coding of splice isoforms is given at the bottom. (**C**) Quantification of endogenous *SMN1/SMN2* splice isoforms identified by MESDA portrayed in Figure 9C (right panel). Labeling is the same as in (B). (**D**) Overexpression of MYC-tagged SRSF3 protein. Left panel: Representative western blots showing expression level of FLAG-tagged SMN protein produced from *SMN2*^{Sup} in HeLa cells simultaneously overexpressing SRSF3. Protein bands identities and antibodies used for membrane probing are indicated. Right panels: Western blot quantifications (**E**) Results of MESDA examining splicing

of *SMN2*^{Sup} (left panel) and endogenous *SMN1/SMN2* (right panel) in the presence of overexpressed SRSF. (**F**) qPCR measuring transcript levels *SMN2*^{Sup} (left panel) and endogenous *SMN1/SMN2* (right panel) in the presence of overexpressed SRSF3. For all western blot and qPCR quantifications, n = 3. Error bars represent SEM. * - $p < 0.05$

Supplementary Figure S18. Effects of DHX9 depletion on *SMN2*^{Sup}: Additional data. (Related to Figure 8) (**A**) Quantification of spliceisoforms identified by MESDA in Figure 8F. siRNA treatments and transfected plasmids are indicated at the bottom of each graph. Color coding of splice isoforms are indicated at the right side (left panel) or below the graph (right panel). (**B**) qPCR measurement of levels of circRNA derived from exons 3 and 4 of *SMN*. Locations of primers used for qPCR are shown in the diagram on the left. **: $p < 0.01$, n.s. – no significant difference. (**C**) qPCR measurement of *SMN2*^{Sup} (left panel) and endogenous *SMN* (right panel) transcript levels in HeLa cells transfected with siRNA against DHX9. *: $p < 0.05$, **: $p < 0.01$.

Supplementary Figure S19. SMN2 super minigene serves as a sensitive reporter for splice modulating antisense oligonucleotides. (Related to Figure 9) (**A**) Brief overview of the mechanism of antisense oligonucleotide (ASO)-mediated correction of *SMN2* exon 7 splicing. Exons are shown as colored shapes, introns as lines/broken lines. The sequence of the first 36 bases of intron 7 are given. The sequence of Anti-N1 ASO is shown in a blue box, base pairing with intronic sequences is shown as black lines. (**B**) Splicing pattern of endogenous *SMN1/SMN2* (left panel) and *SMN2*^{Sup} (right panel) in HeLa cells transfected with *SMN2*^{Sup757} with and without co-transfection of Anti-N1, as determined by MESDA. Labeling is the same as Figure 1C. Abbreviations: Scr. ASO, scrambled ASO; pCI, empty pCI-Neo. (**C**) Quantification of splice isoforms identified by MESDA in (B). Transfected plasmids and ASOs, and color-coded splice isoforms are specified at the bottom of the graphs. (**D**) Representative western blots showing expression levels of endogenous SMN and 3XFLAG-taged SMN derived from *SMN2*^{Sup}. Transfected plasmids and ASOs are indicated at the top. Antibodies used to probe membrane are indicated at the left side, protein band identities are indicated at the right. Abbreviations: UTC, untransfected control; pCI, control transfected with empty pCI-Neo vector alone; SCR, control transfected with scrambled ASO; a-N1, sample transfected with Anti-N1

ASO. (**E**) Quantification of western blot from (D). Values are calculated by dividing signal for 3XFLAG-taged SMN by endogenous SMN using anti-SMN antibody. Error bars represent the standard error of the mean. n = 3. **: $p < 0.01$.

Supplementary Figure S20. Quantification of splice isoforms identified in Figure 9. (**A**) Quantification of splice isoforms identified by MESDA in Figure 9B. Super minigenes, cell types, and color-coded splice isoforms are indicated at the bottom of the graph. Location of primers used is shown at the top of the graph. (**B**) Quantification of splice isoforms identified by MESDA in Figure 9C. Labeling is the same as in (A).

Supplementary Figure S1.

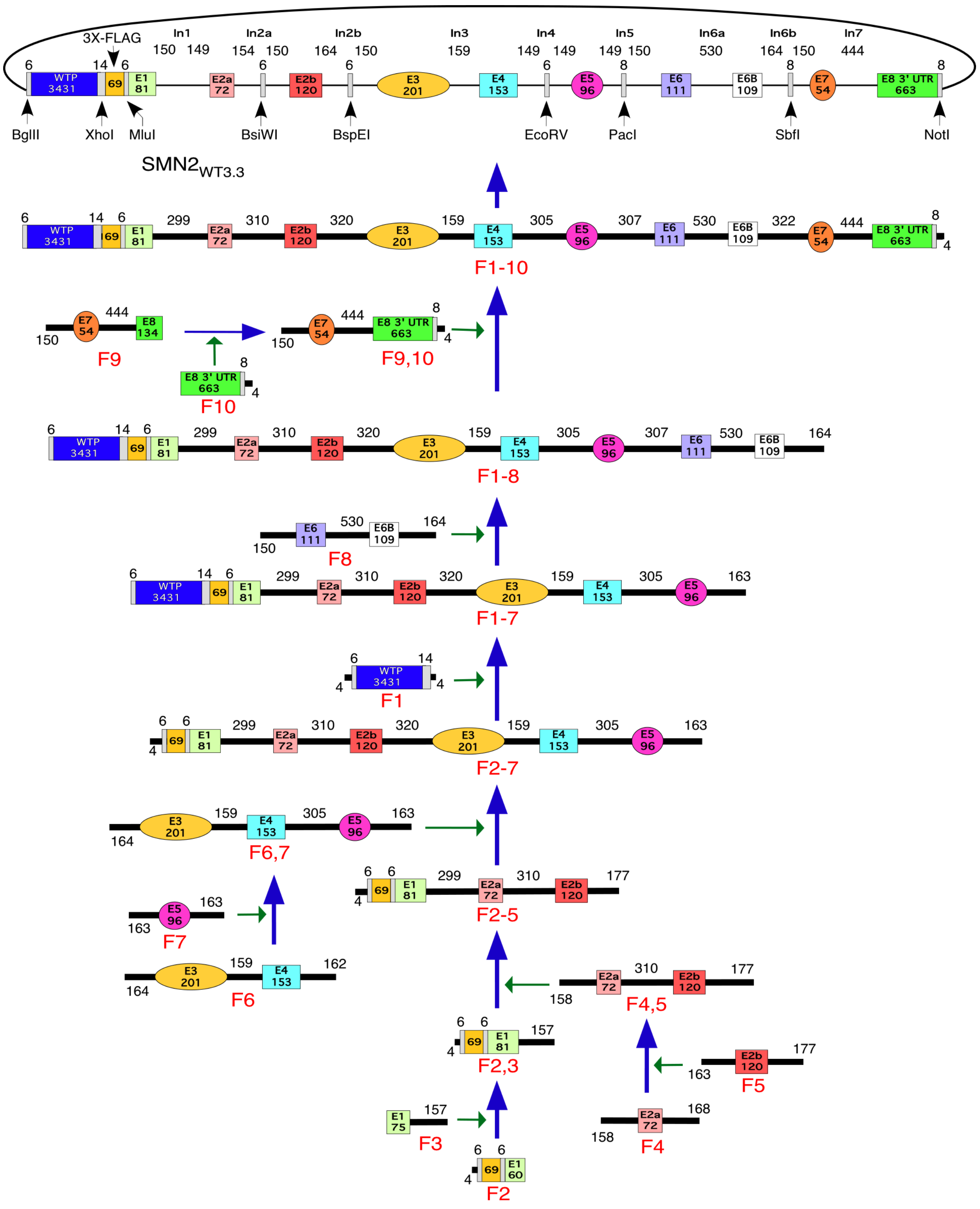

Supplementary Figure S2.

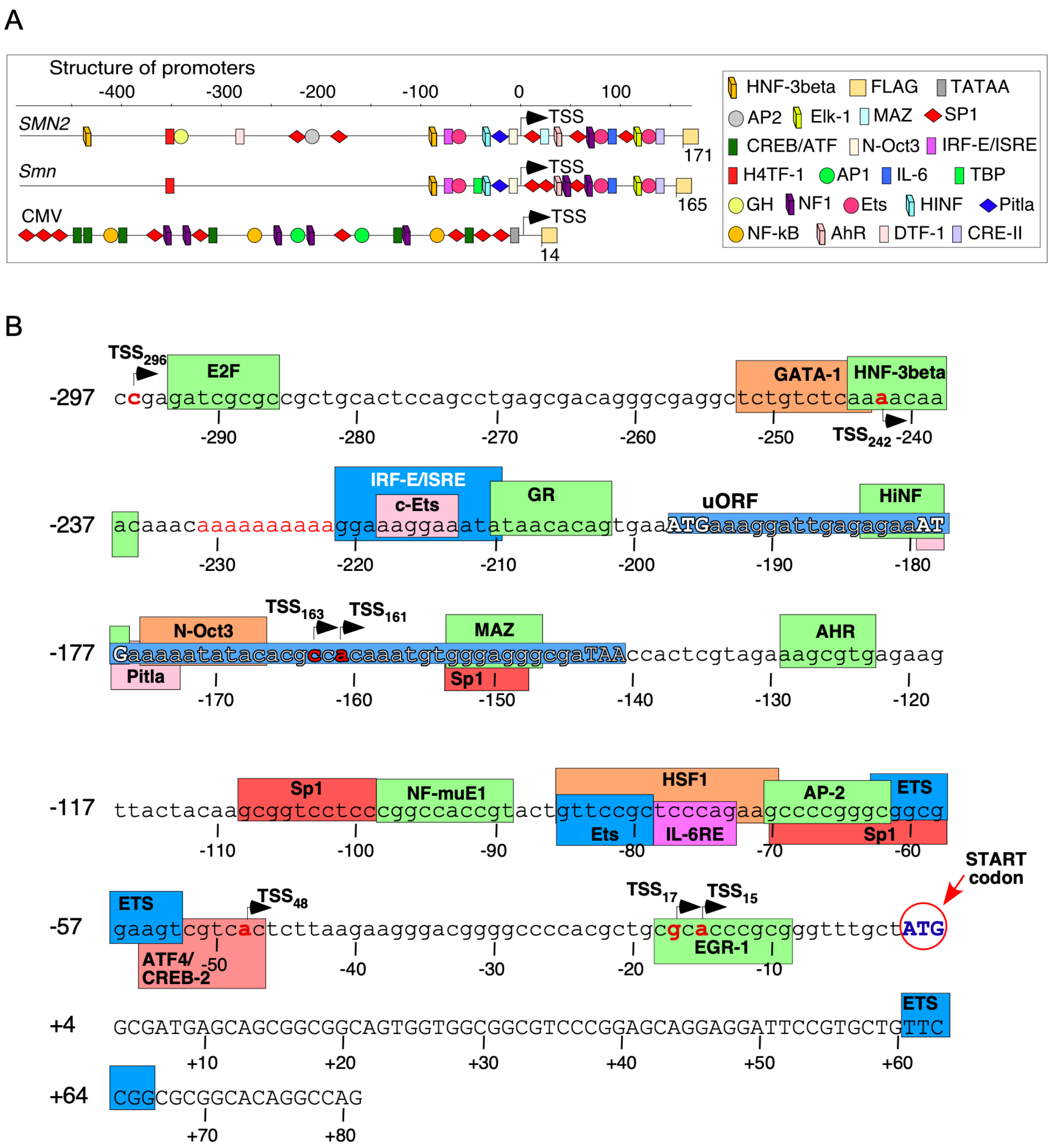

Supplementary Figure S3.

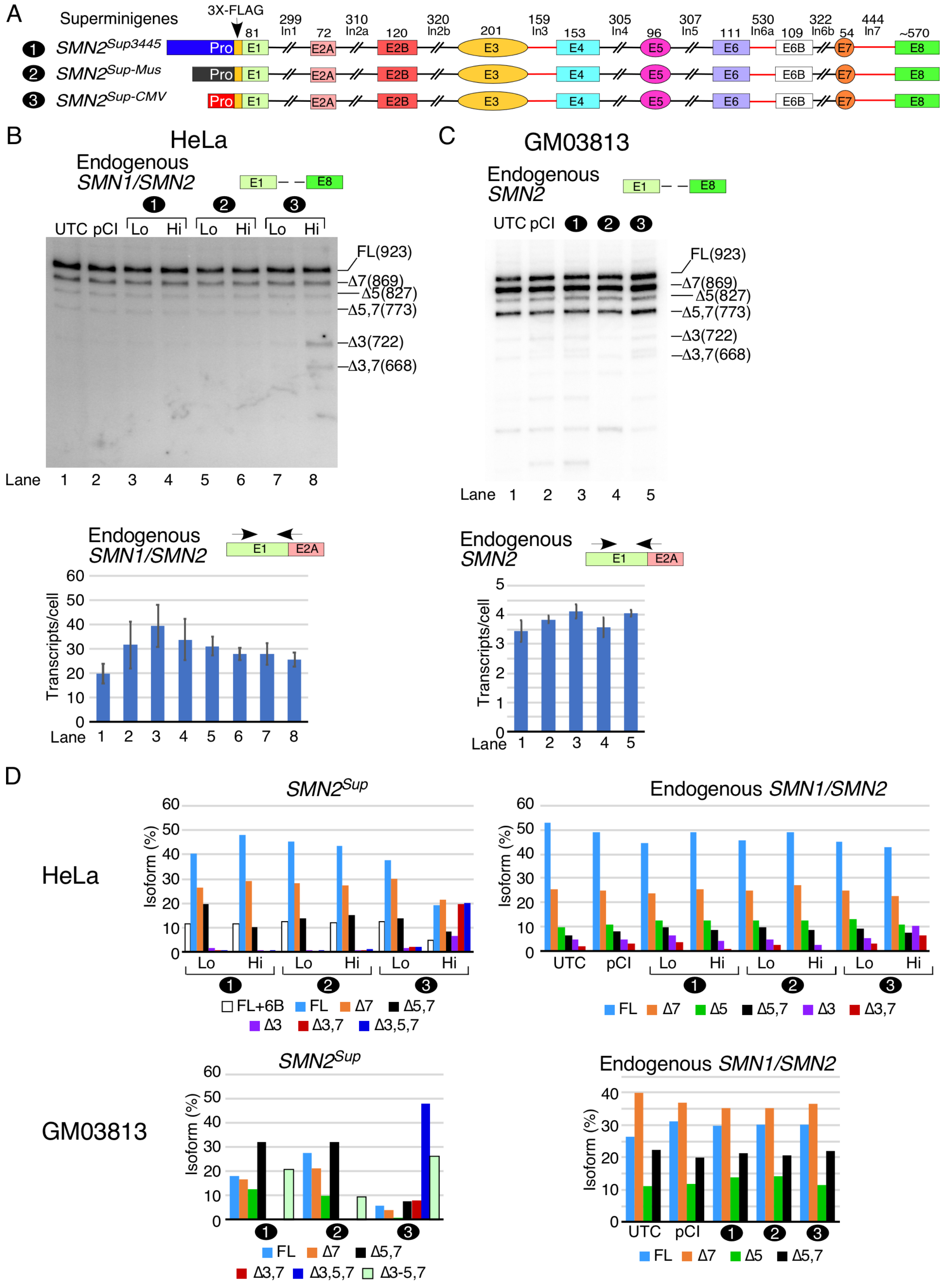

Supplementary Figure S4.

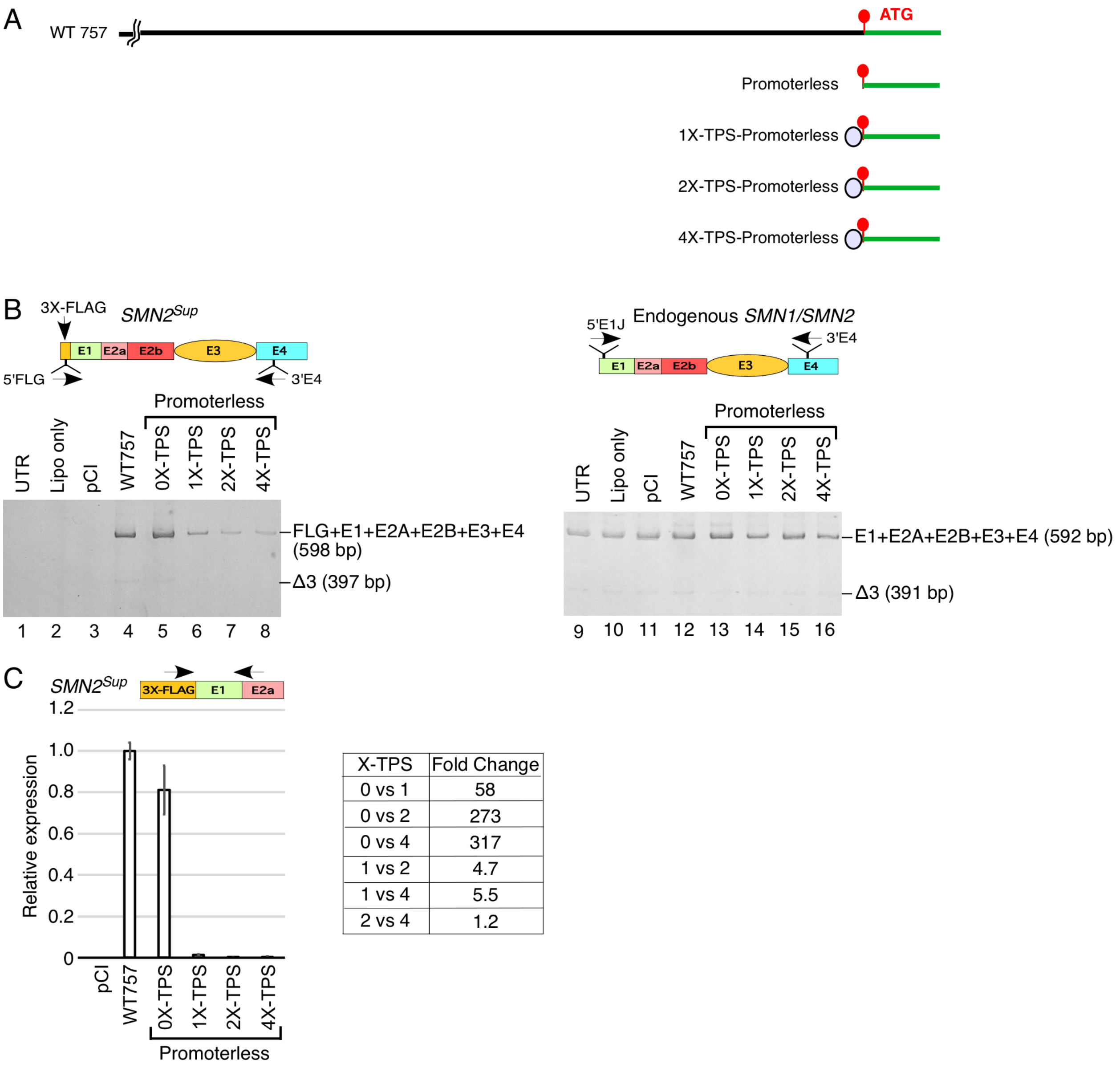

X-TPS	Fold Change
0 vs 1	58
0 vs 2	273
0 vs 4	317
1 vs 2	4.7
1 vs 4	5.5
2 vs 4	1.2

Supplementary Figure S5.

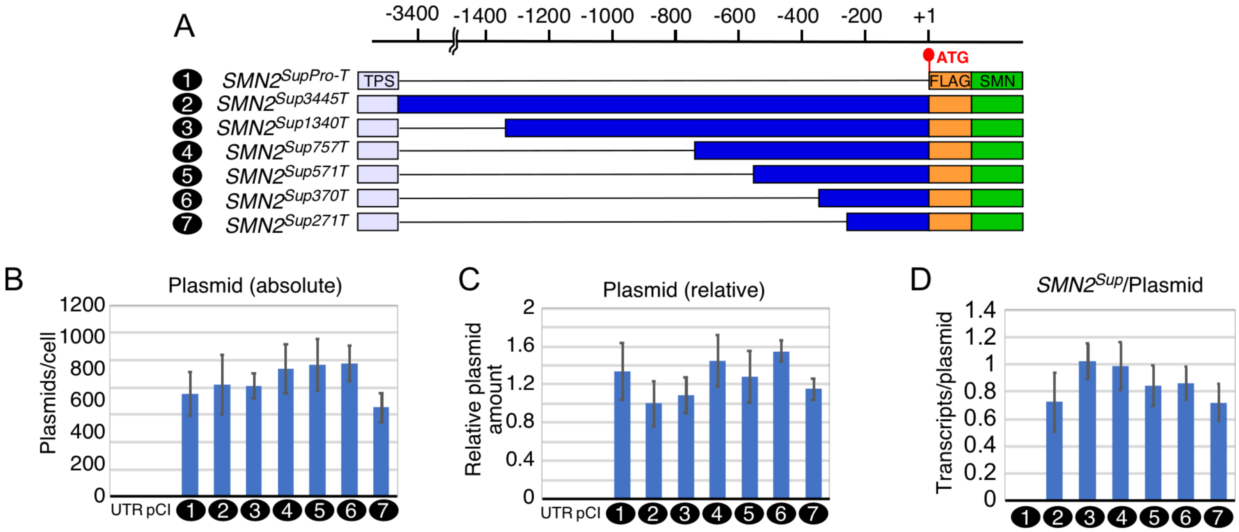

Supplementary Figure S6.

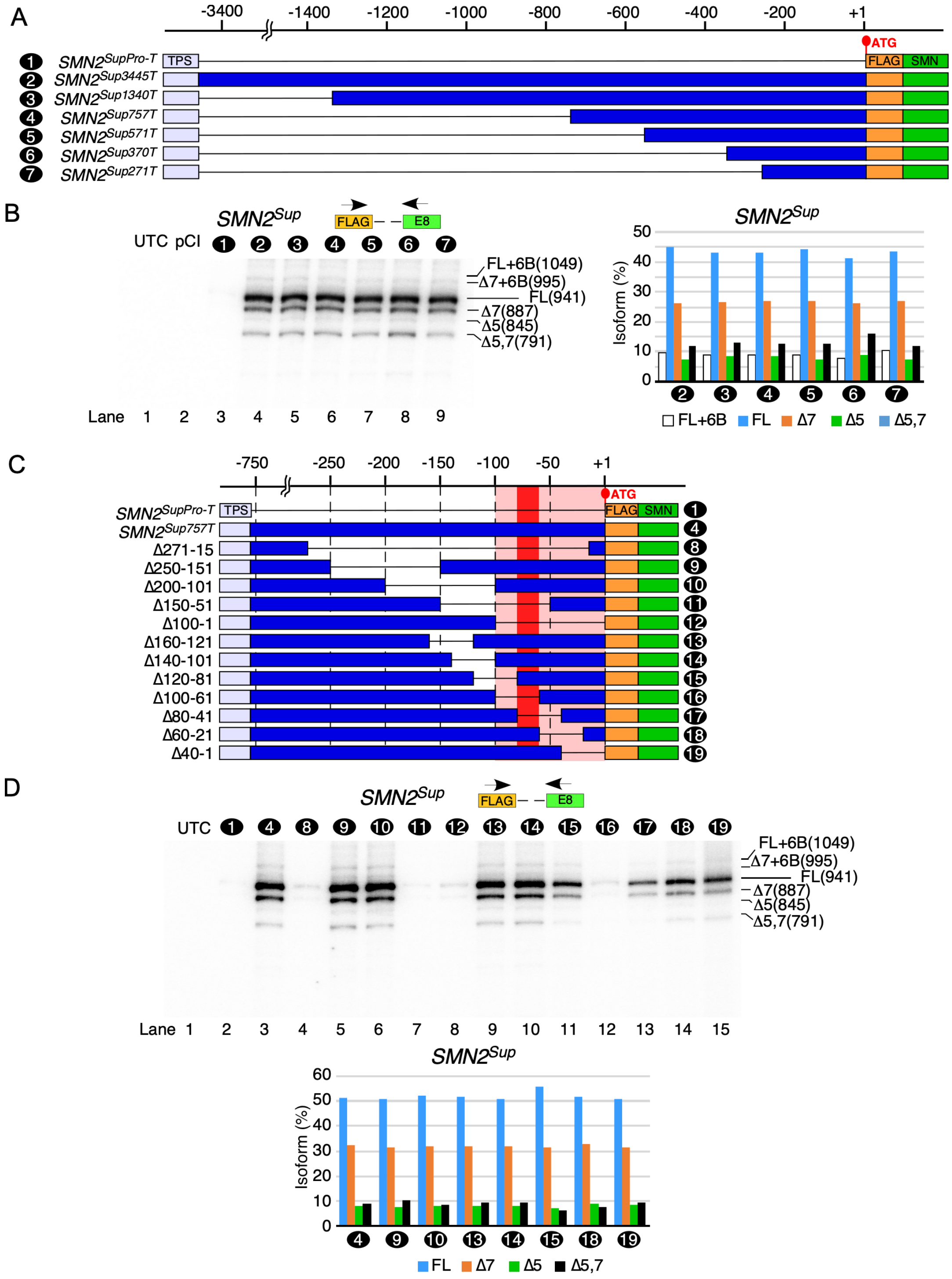

Supplementary Figure S7.

A

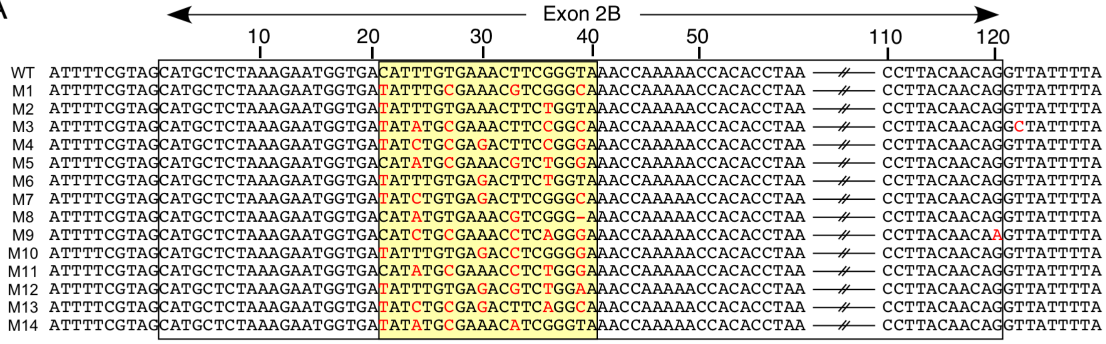

B

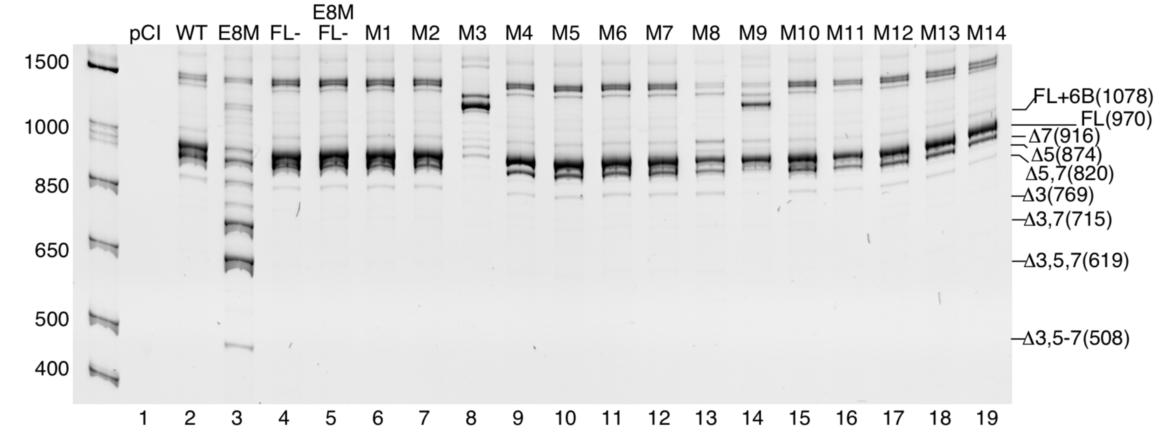

C

	Average Ct	
Sample	Constant	Mutated
WT	16.71	15.85
M1	16.91	N/A
M2	16.64	34.13
M4	16.59	N/A
M5	16.83	N/A
M6	16.65	38.28
M7	17.06	N/A
M10	16.86	N/A
M11	16.60	N/A
M12	16.60	N/A
M13	16.75	39.00
M14	16.63	N/A

D

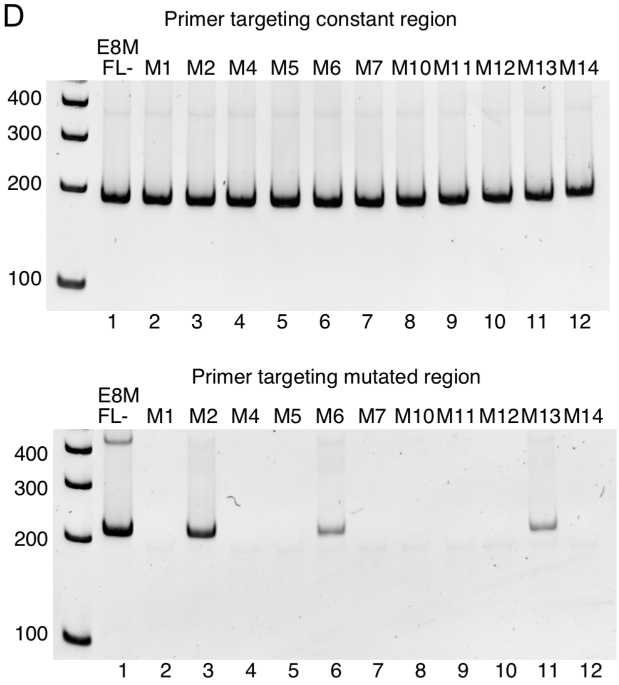

Supplementary Figure S8.

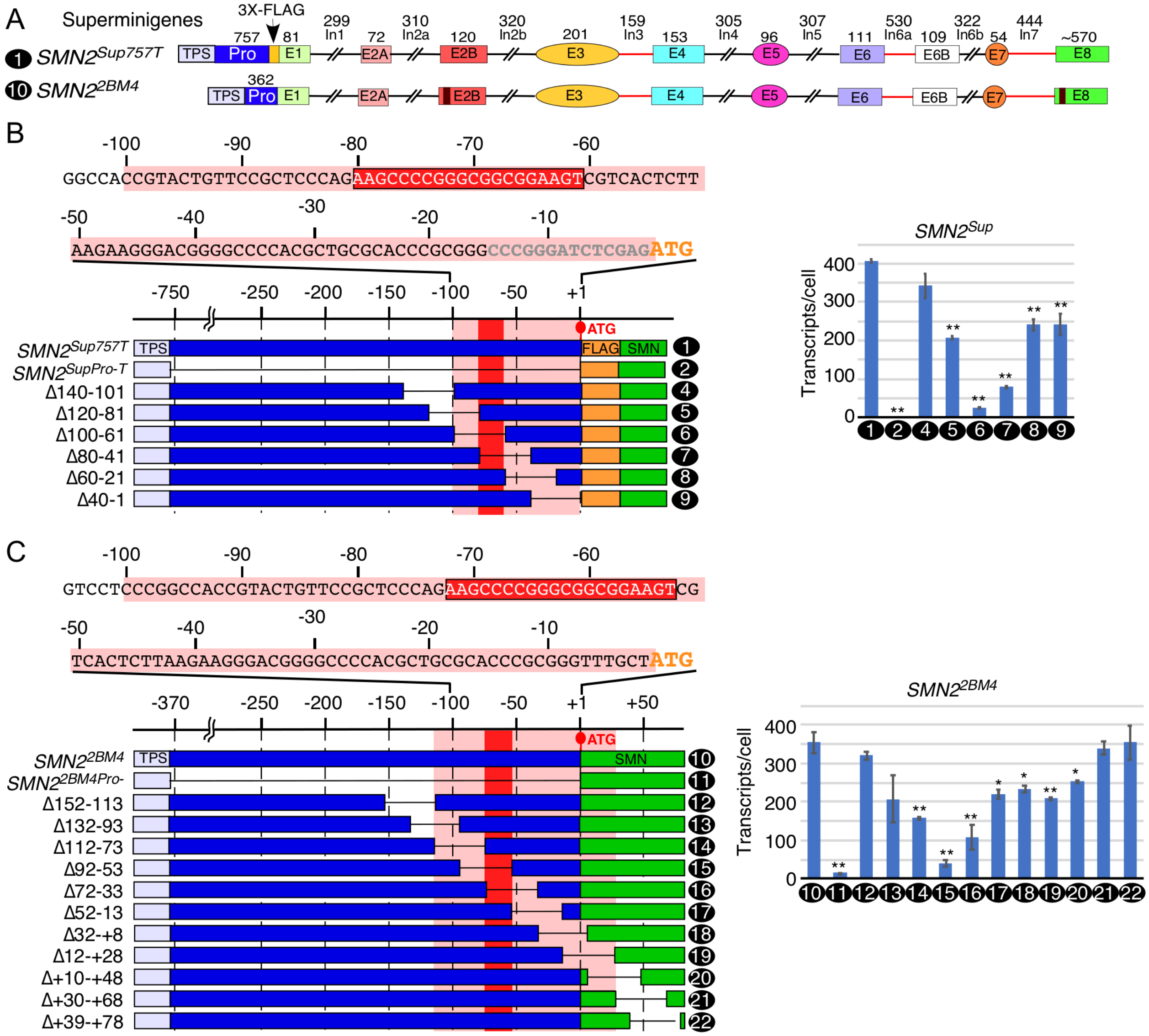

Supplementary Figure S9.

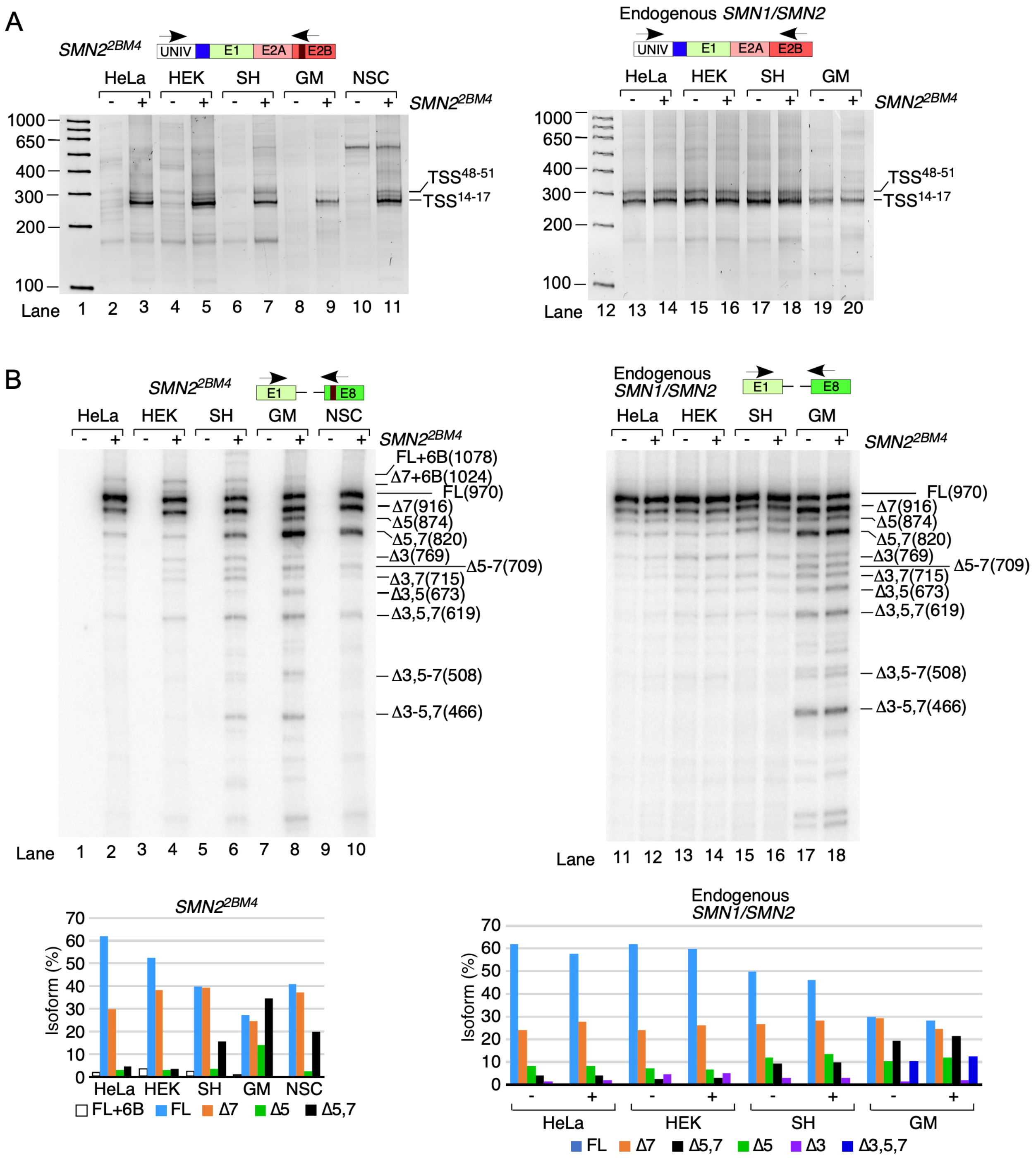

Supplementary Figure S10.

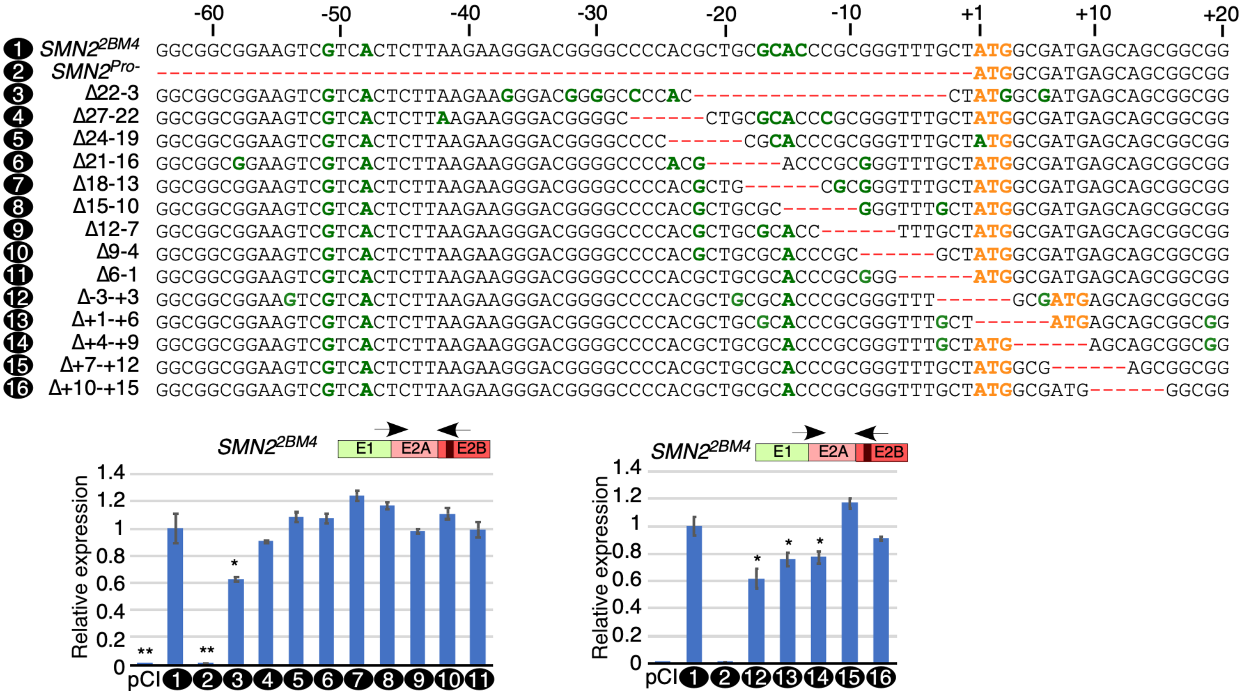

Supplementary Figure S11.

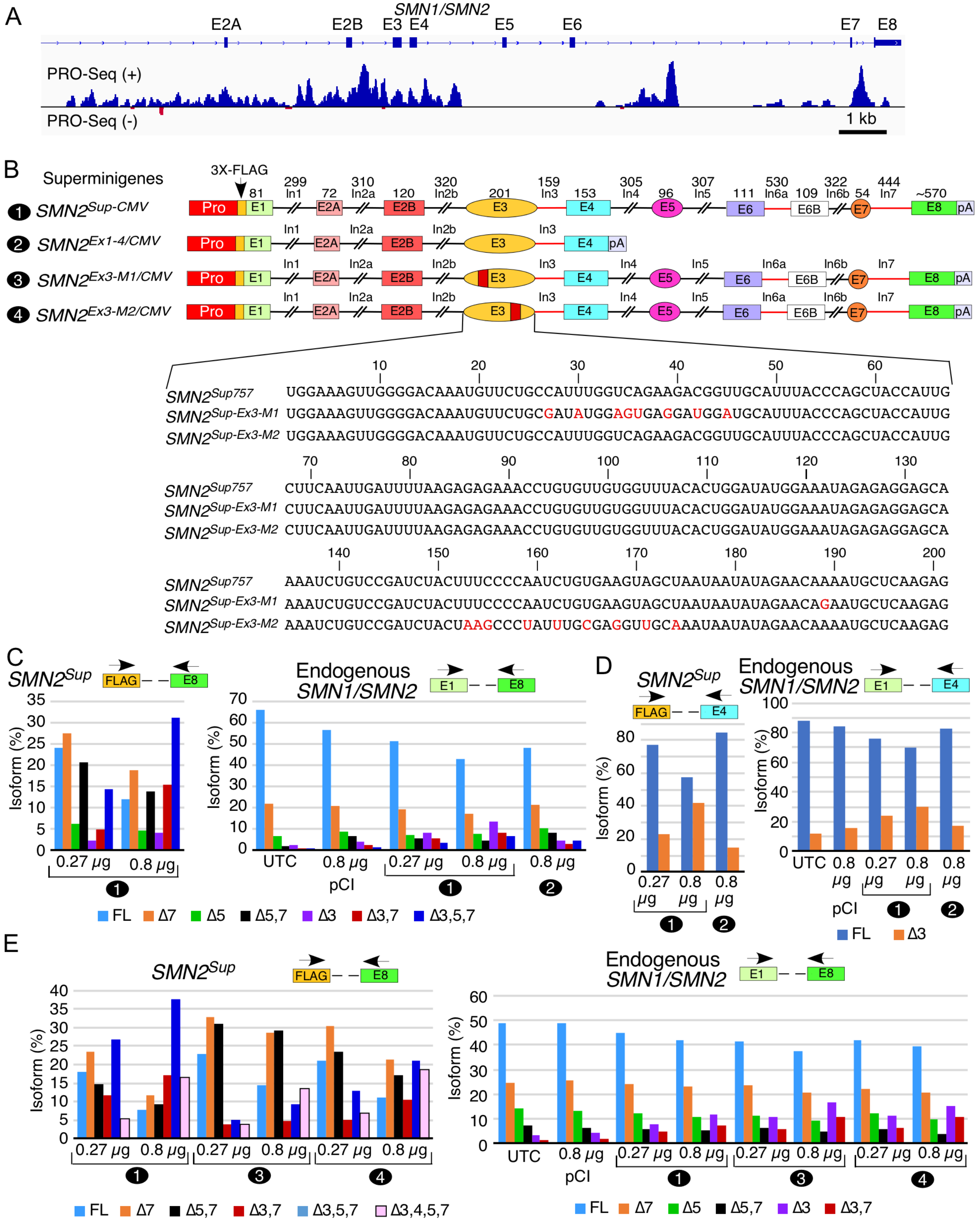

Supplementary Figure S12.

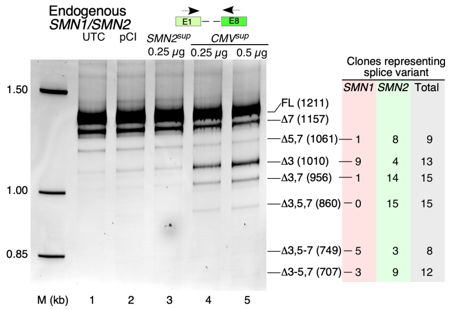

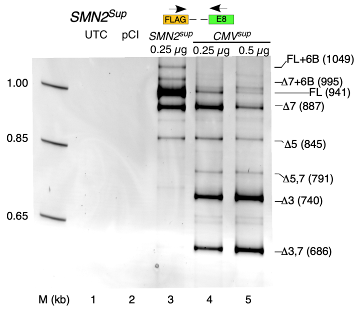

Supplementary Figure S13.

A

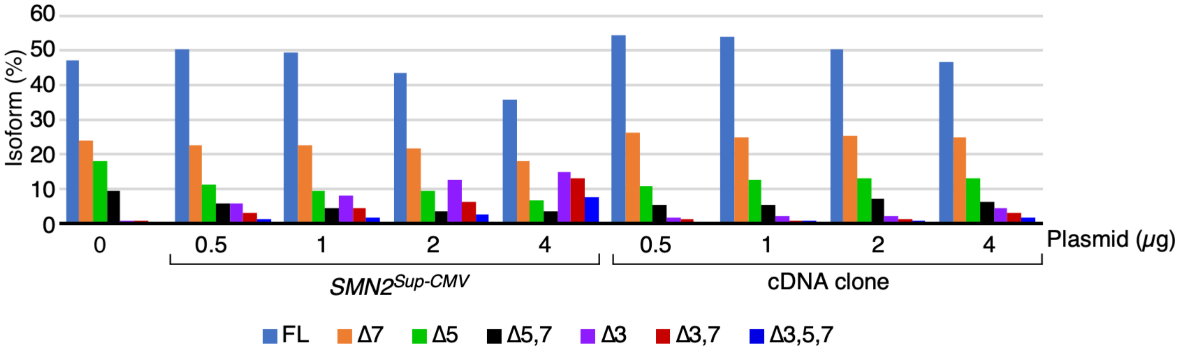

B

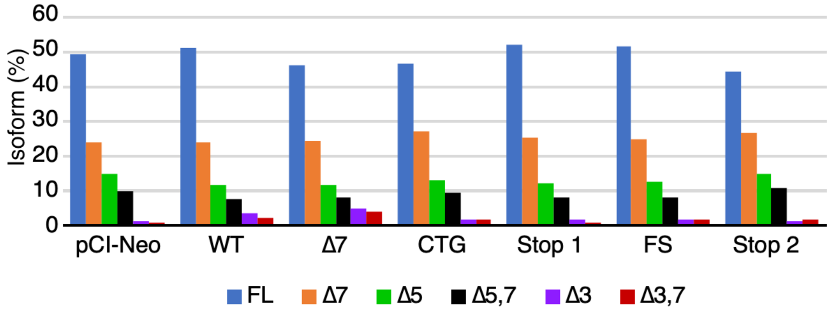

Supplementary Figure S14.

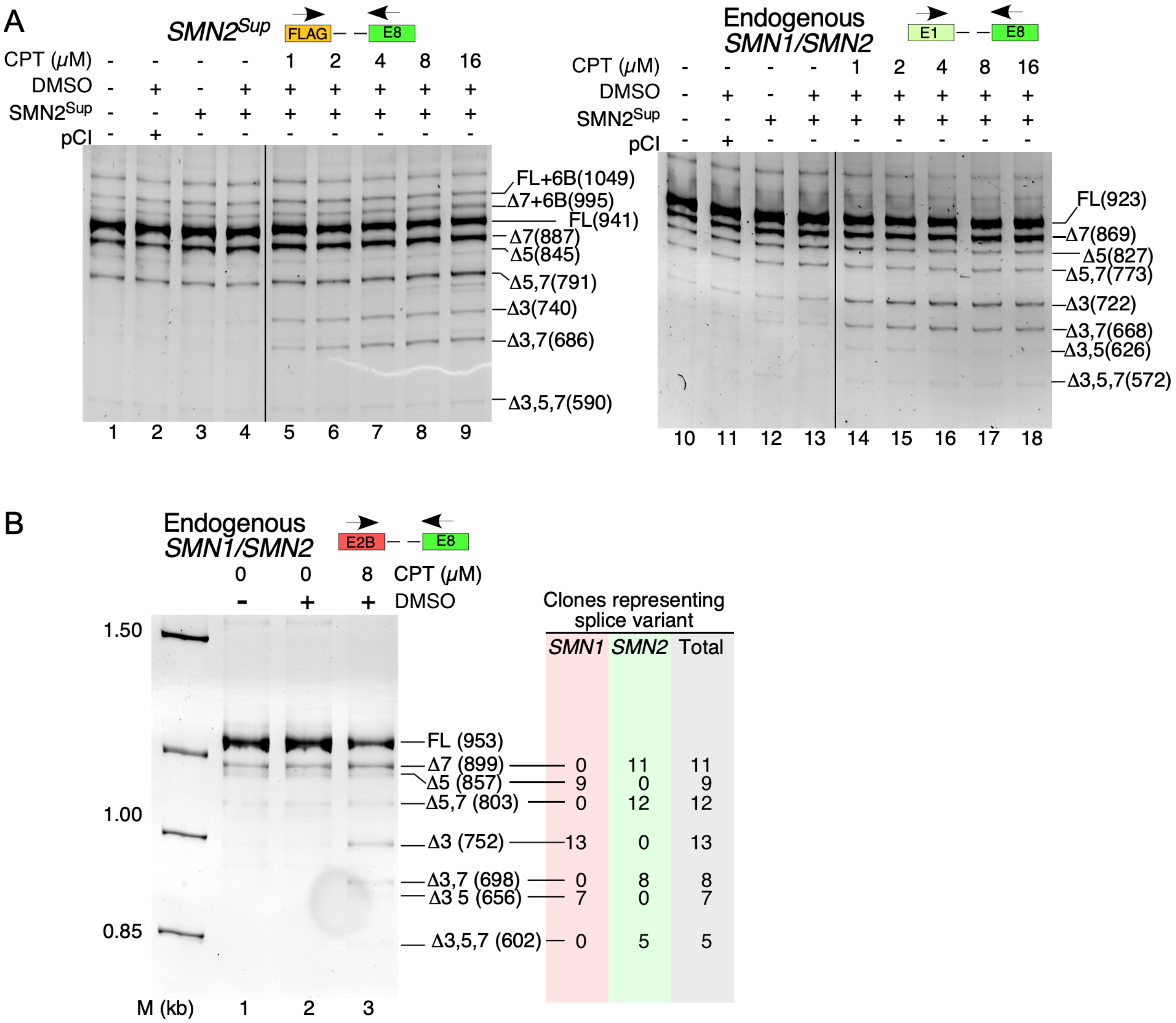

Supplementary Figure S15.

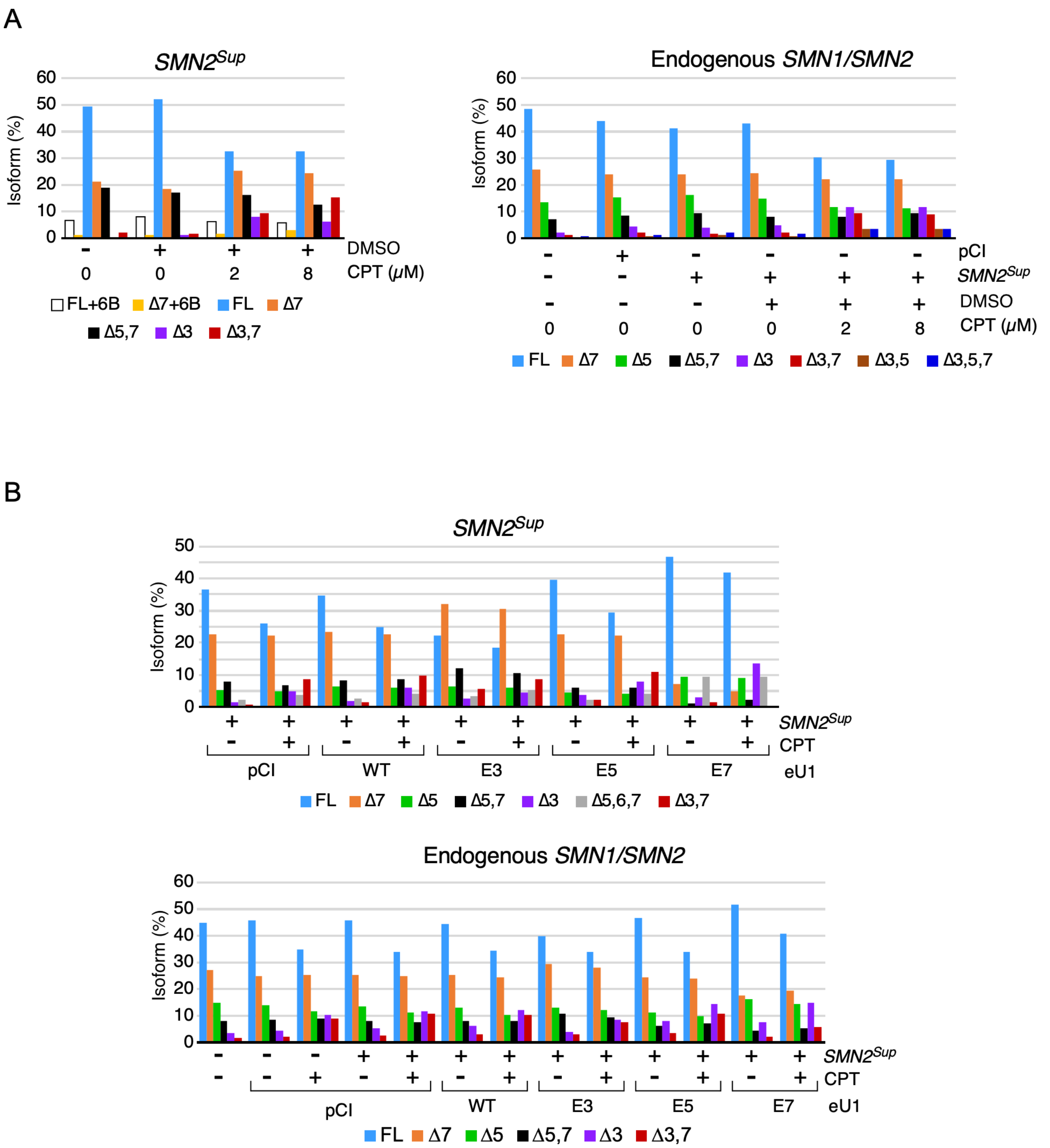

Supplementary Figure S16.

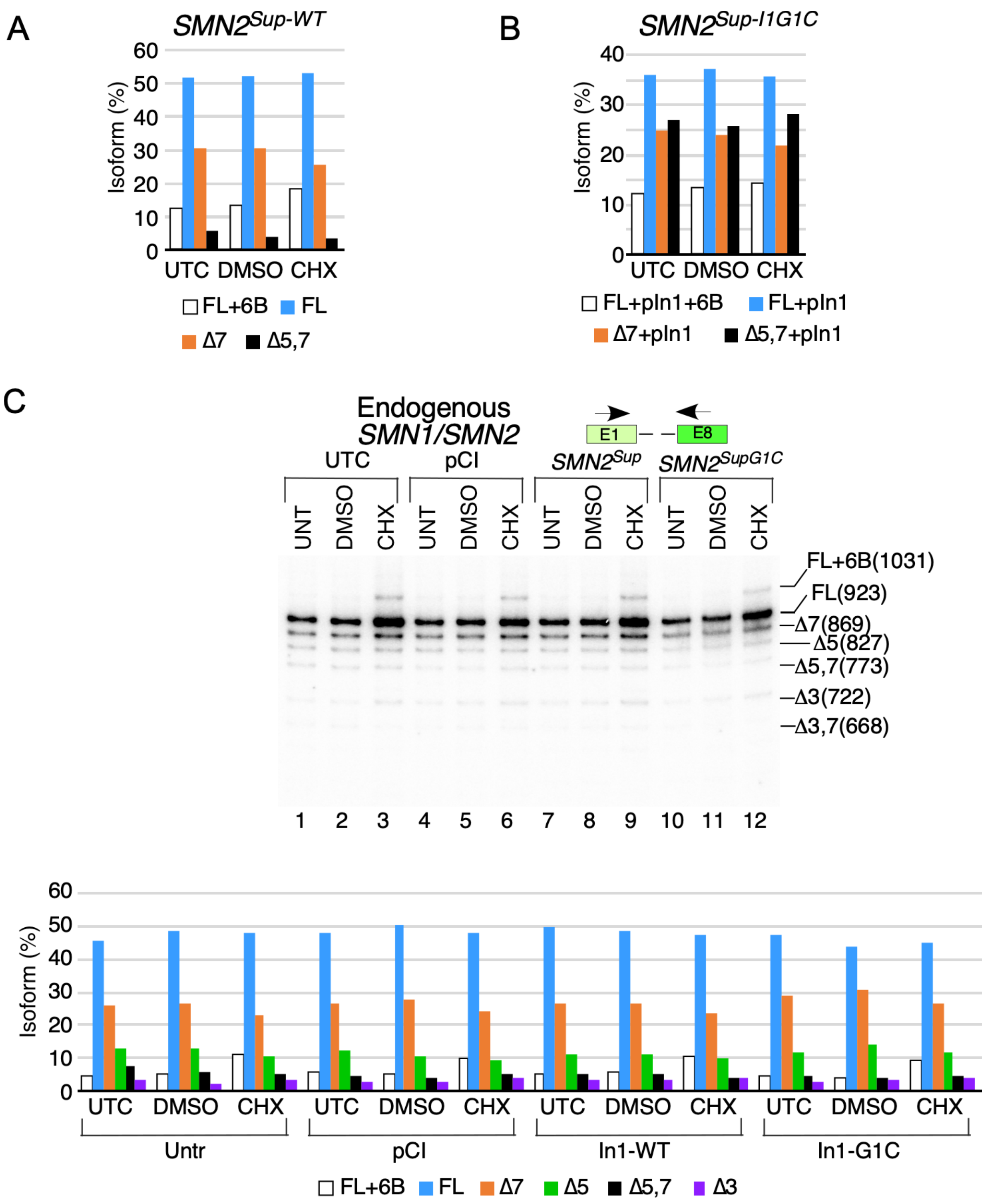

Supplementary Figure S17.

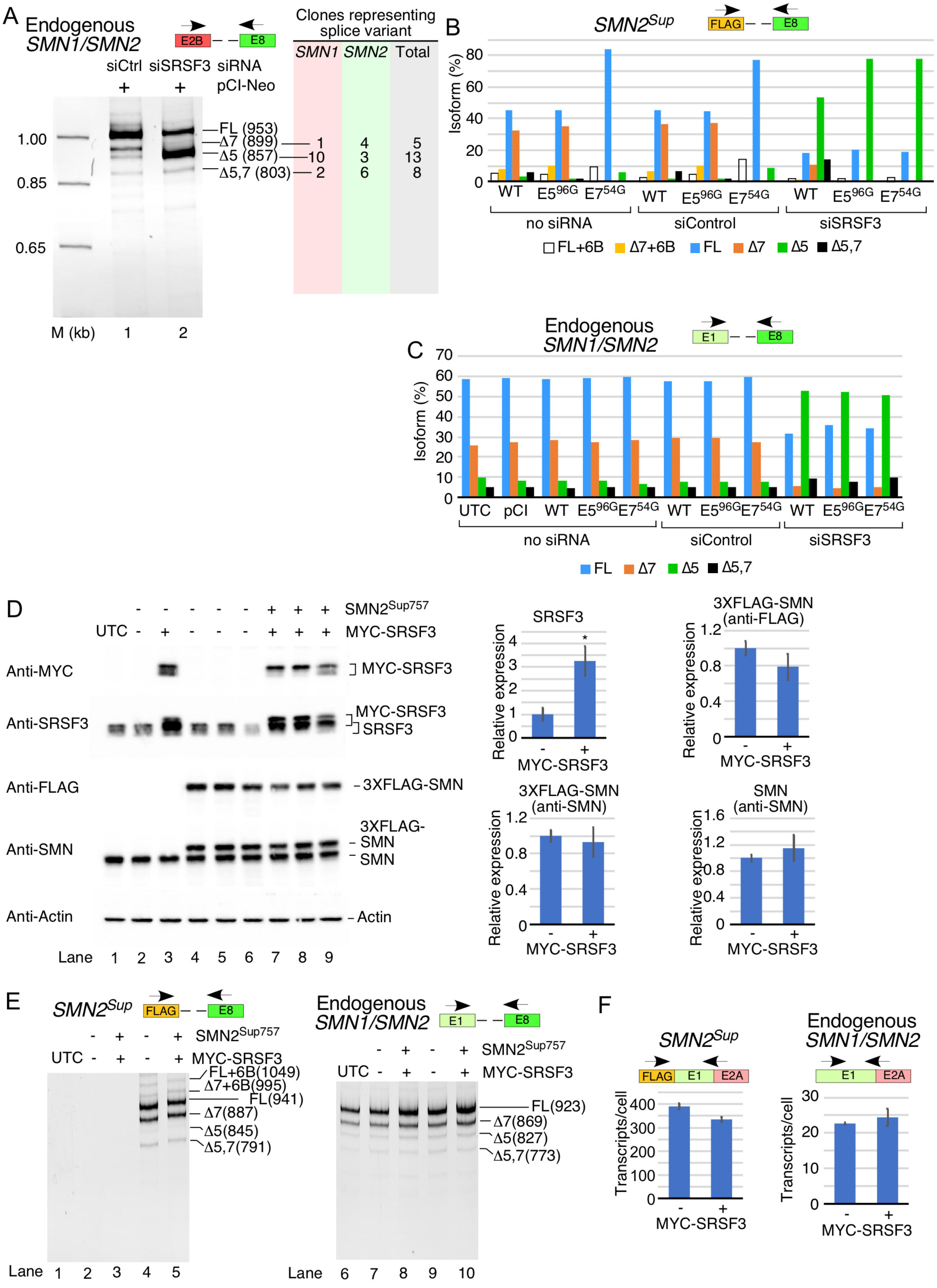

Supplementary Figure S18.

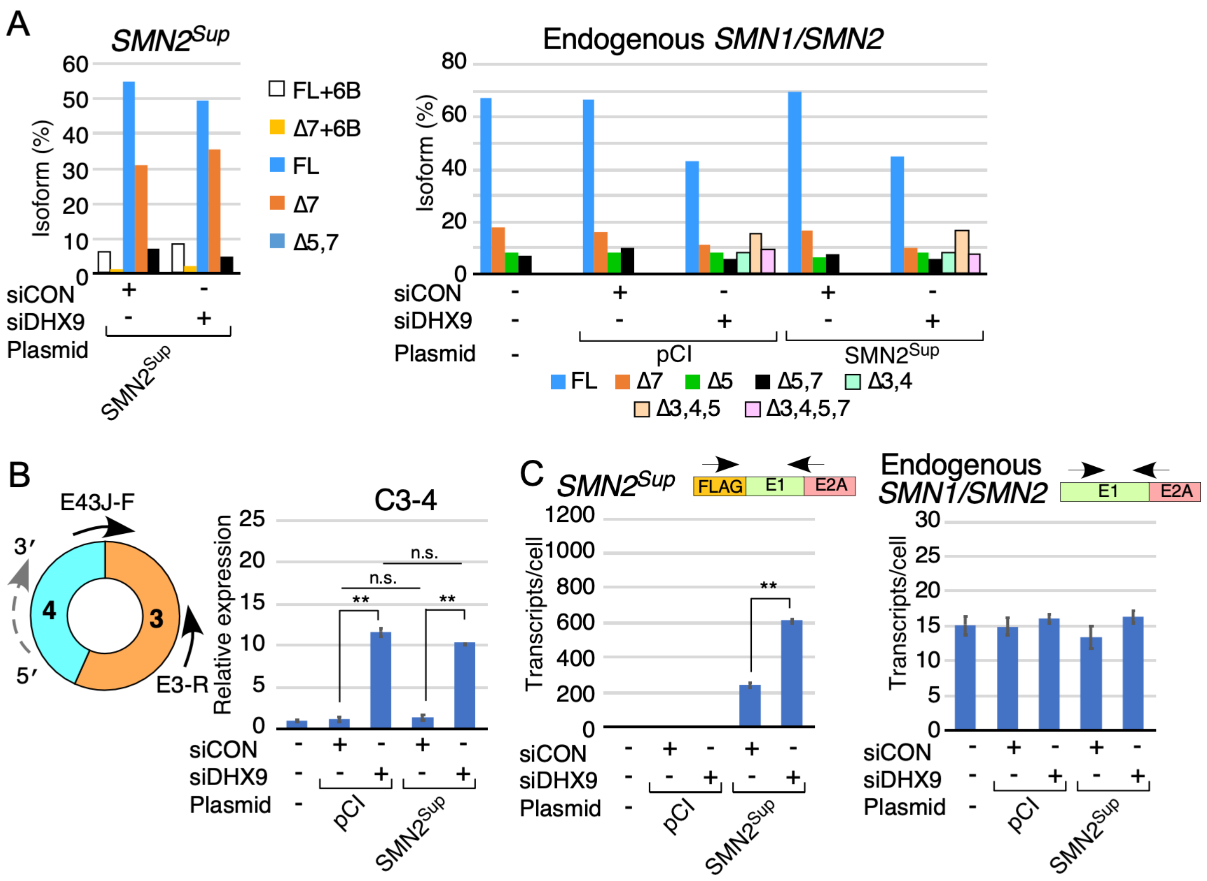

Supplementary Figure S19.

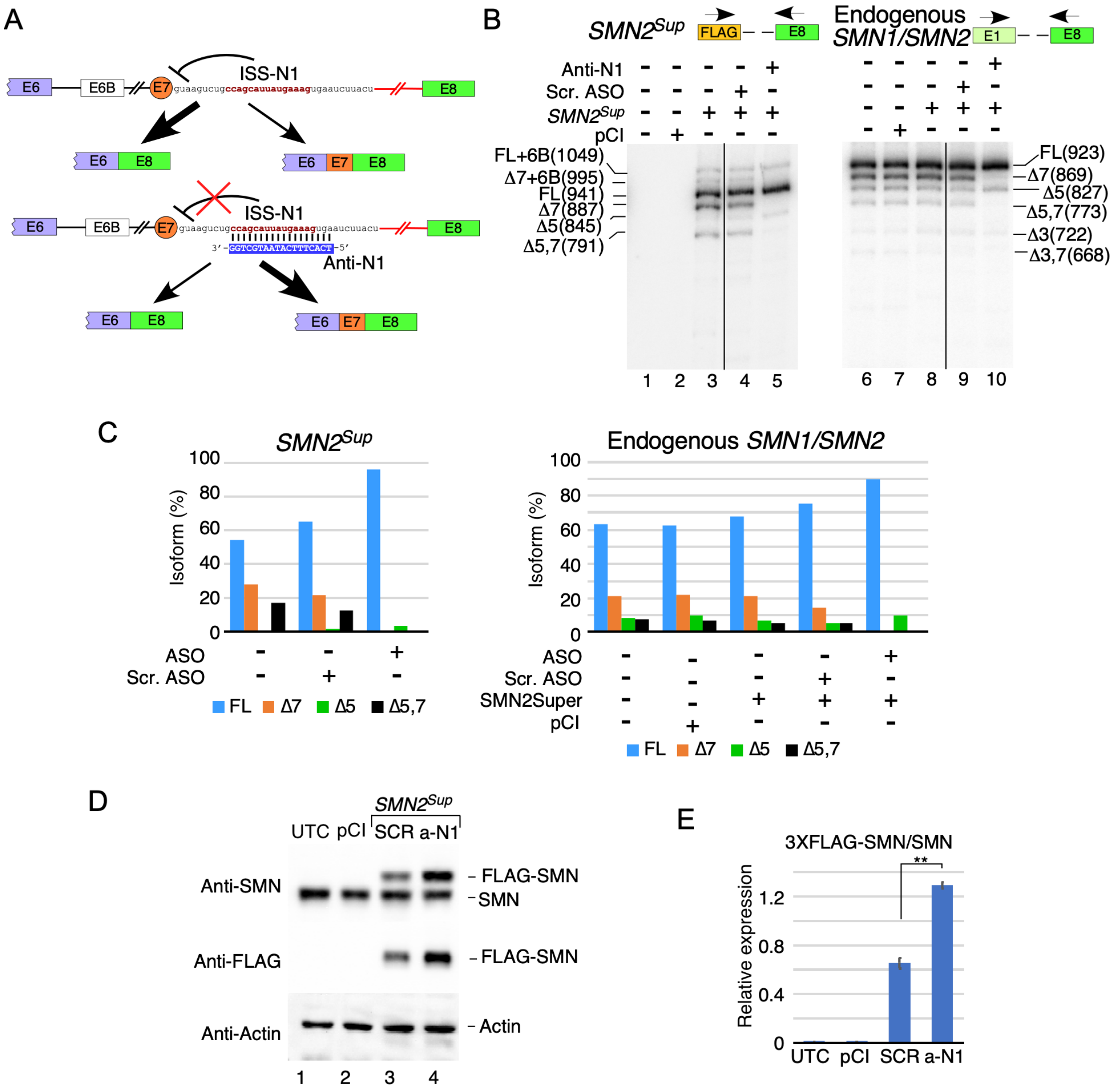

Supplementary Figure S20.

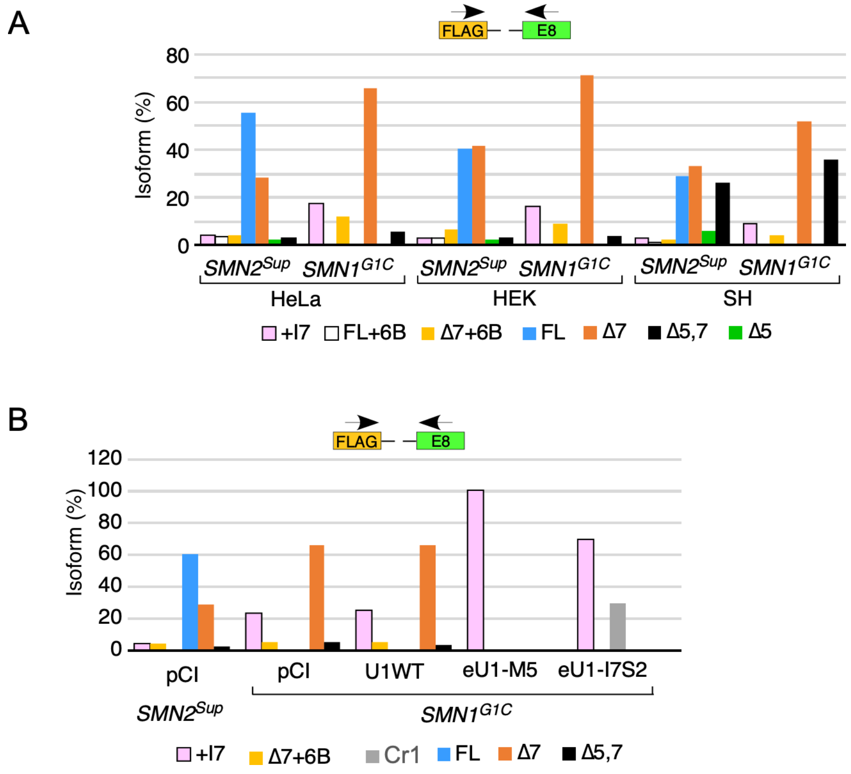

Supplement: gkad1259_Supplemental_Files [file gkad1259_supplemental_files.zip › Supplementary Data 12-19-2023.pdf]
